# Celastrol and Its Derivatives in Breast, Ovarian, and Cervical Cancer: Molecular Targets, Signalling Networks, and Nanocarrier-Based Delivery

**DOI:** 10.3390/molecules31203641

**Published:** 2026-10-12

**Authors:** Anna Merecz-Sadowska, Maciej Kowalski, Tomasz Kowalczyk, Maksymilian Trzciński, Martyna Muskała, Janusz Piekarski, Bartosz Tylkowski, Laurent Picot, Filippo Maggi, Przemysław Sitarek

**Affiliations:** 1 Department of Economic and Medical Informatics, University of Lodz, 90-214 Lodz, Poland; 2 Students Research Group, Department of Medical Biology, Medical University of Lodz, 90-151 Lodz, Poland; maciej.kowalski1@student.umed.lodz.pl (M.K.); martyna.muskala@stud.umed.lodz.pl (M.M.); 3 Department of Molecular Biotechnology and Genetics, Faculty of Biology and Environmental Protection, University of Lodz, Banacha 12/16, 90-237 Lodz, Poland; tomasz.kowalczyk@biol.uni.lodz.pl; 4 Students Research Club NANO, Department of Chemistry, Lodz University of Technology, Stefanowskiego 16, 90-537 Lodz, Poland; 256160@edu.p.lodz.pl; 5 Department of Surgical Oncology, Medical University of Lodz, 251 Pomorska St., 93-513 Lodz, Poland; janusz.piekarski@umed.lodz.pl; 6 Ludwik Rydygier Collegium Medicum in Bydgoszcz, Nicolaus Copernicus University in Toruń, Jagiellońska 13-15, 85-067 Bydgoszcz, Poland; bartosz.tylkowski@eurecat.org; 7 Eurecat, Centre Tecnològic de Catalunya, C/Marcel·lí Domingo 2, 43007 Tarragona, Spain; 8 Littoral Environnement et Sociétés UMRi CNRS 7266 LIENSs, La Rochelle Université, 17042 La Rochelle, France; laurent.picot@univ-lr.fr; 9 Chemistry Interdisciplinary Project (ChIP) Research Center, School of Pharmacy, University of Camerino, Via Madonna delle Carceri, 62032 Camerino, Italy; filippo.maggi@unicam.it; 10 Department of Medical Biology, Medical University of Lodz, Muszyńskiego 1, 90-151 Lodz, Poland

**Keywords:** celastrol, tripterine, *Tripterygium wilfordii*, breast cancer, ovarian cancer, cervical cancer, quinone methide, regulated cell death, nanomedicine, pharmacoeconomics

## Abstract

Celastrol, a quinone methide pentacyclic triterpenoid obtained mainly from the roots of *Tripterygium wilfordii* Hook. f., forms covalent, often reversible, adducts with cysteine thiols in regulatory proteins, which underlies its multi-targeted action. This review summarises the preclinical evidence on celastrol and its derivatives in breast, ovarian, and cervical cancer and the nanocarriers designed to overcome its pharmacological limitations. Chemoproteomics has defined covalent targets at three distinguishable levels of evidence: residue-mapped and confirmed by mutagenesis or by genetic validation for IKKβ, PRDX1, PKM2, HMGB1, and COMMD3; binding measured without mapping of the residue for the Hsp90–Cdc37 complex, the proteasome, further peroxiredoxins, and mitochondrial isocitrate dehydrogenases; and inferred from functional or degradation readouts for the remainder. Almost all of this work was performed outside the three tumour types, so the target catalogue applies to them by extrapolation. Across in vitro and in vivo models it induces cell-cycle arrest, apoptosis, and oxidative and mitochondrial stress, modulates NF-κB, PI3K/Akt/mTOR, JAK/STAT3, MAPK, Wnt/β-catenin, and Hippo signalling, and limits migration, invasion, and angiogenesis. It also engages paraptosis, endoplasmic reticulum stress, pyroptosis, ferroptosis, cuproptosis, and immunogenic cell death, and remodels the tumour immune microenvironment. Modification at the C-20 carboxyl group and the A/B ring system, including proteolysis-targeting chimeras, has improved potency and selectivity. Because solubility, oral bioavailability, and the therapeutic window remain limiting, liposomal, micellar, carrier-free, and biomimetic nanocarriers have been developed to improve tumour selectivity and safety. Translation will depend on optimised derivatives, advanced delivery, sustainable production, rigorous pharmacokinetic and toxicological evaluation, and the pharmacoeconomic considerations examined here.

## 1. Introduction

For decades, natural products have been one of the most important sources of compounds used in the development of new anticancer drugs. It is estimated that over 60% of currently used anticancer drugs are derived directly or indirectly from natural compounds or their derivatives [1,2]. Examples include vincristine, vinblastine, paclitaxel, and irinotecan, which are successfully used in oncological therapies [3,4,5]. Among the bioactive secondary metabolites of plants, celastrol, also known as tripterine, is particularly noteworthy, having become the subject of intensive research in recent years due to its multifaceted biological effects [6].

Celastrol is a natural pentacyclic triterpenoid of the quinone methide type, isolated primarily from the roots of *Tripterygium wilfordii* Hook. f., a plant of the Celastraceae family that has been used for centuries in traditional Chinese medicine to treat inflammatory, rheumatic, and autoimmune diseases [6,7,8]. This compound also occurs in other members of the *Tripterygium* and *Celastrus* genera, as well as in the genus *Maytenus*. Over the past two decades, it has attracted intensive preclinical interest owing to its broad spectrum of biological activity and its ability to modulate numerous cellular processes associated with the development of chronic diseases, including cancer [9]. Chemically, celastrol belongs to the group of pentacyclic triterpenoids with a friedelane skeleton and the molecular formula C_29_H_38_O_4_. A characteristic structural element is a highly reactive quinone methide system, which is responsible for the electrophilic properties of the molecule and for its ability to interact with the thiol groups of cysteine residues in numerous regulatory proteins [6,10]. Owing to this structure, celastrol can modulate the activity of signalling pathways involved in cell proliferation, the inflammatory response, oxidative stress, and cancer cell survival. The carboxyl group at C-20 also permits chemical modification aimed at improving the pharmacokinetic properties of the compound and at developing derivatives with greater therapeutic efficacy and reduced toxicity [10].

The growing interest in celastrol stems from its pleiotropic biological effects. In vitro and in vivo studies have demonstrated that this compound possesses anti-inflammatory [9], antioxidant [11], antibacterial [12], antiviral [13,14], neuroprotective [15], and anticancer [16] properties. In experimental models, celastrol has been shown to inhibit cancer cell proliferation, induce apoptosis and autophagy, limit angiogenesis, and suppress migration and invasion. These effects result from the simultaneous modulation of numerous signalling pathways, such as nuclear factor κB (NF-κB), PI3K/Akt/mTOR, JAK/STAT3, MAPK, Wnt/β-catenin, hypoxia-inducible factor 1α (HIF-1α), and Nrf2/Keap1, which play a key role in the development and progression of cancer [6,14,17,18].

Particularly consistent results have been obtained in cancers in women. Numerous experimental studies have shown that celastrol inhibits the growth of ovarian [19,20], cervical [21], and breast cancer cells [22] by arresting the cell cycle [19,20], activating apoptotic pathways, and limiting migration and invasion [19,20,21,22]. Related activity has been reported in benign gynaecological disease, in which celastrol induces apoptosis and autophagy in human endometriotic cells [23]. The compound has also been shown to protect normal ovarian tissue from cisplatin-induced toxicity in vivo [24]. Accumulating evidence further indicates its potential to overcome multidrug resistance (MDR), which remains one of the main causes of therapeutic failure in patients with advanced cancers [25].

Despite these favourable biological properties, the clinical application of celastrol remains limited. This compound is characterised by very low aqueous solubility, high lipophilicity, low oral bioavailability, and a relatively narrow therapeutic window [26]. Preclinical studies have also demonstrated the potential for adverse effects, including hepatotoxicity, nephrotoxicity, and reproductive toxicity at higher doses [26,27,28]. These limitations significantly hinder the translation of laboratory findings into clinical practice and constitute one of the main obstacles to the development of celastrol-based therapies. In response to these challenges, nanotechnology-based drug delivery systems are being developed intensively. Nanocarriers such as liposomes, polymeric micelles, lipid nanoparticles, polymeric nanoparticles, and exosome-based systems increase the solubility and stability of celastrol, improve its bioavailability, and allow more selective delivery to tumour tissue [28,29,30]. This approach reduces systemic toxicity and increases the compound’s therapeutic efficacy, making nanotechnology a leading direction in this field [29,30].

This article presents a comprehensive overview of the current state of knowledge regarding the biological properties and molecular mechanisms of action of celastrol, with particular emphasis on its anticancer potential in cancers in women, namely breast, ovarian, and cervical cancer. Furthermore, the latest advances in nanotechnology-based celastrol delivery systems, which aim to increase bioavailability, reduce toxicity, and improve therapeutic efficacy, are discussed.

## 2. Study Design

**Figure 1:**
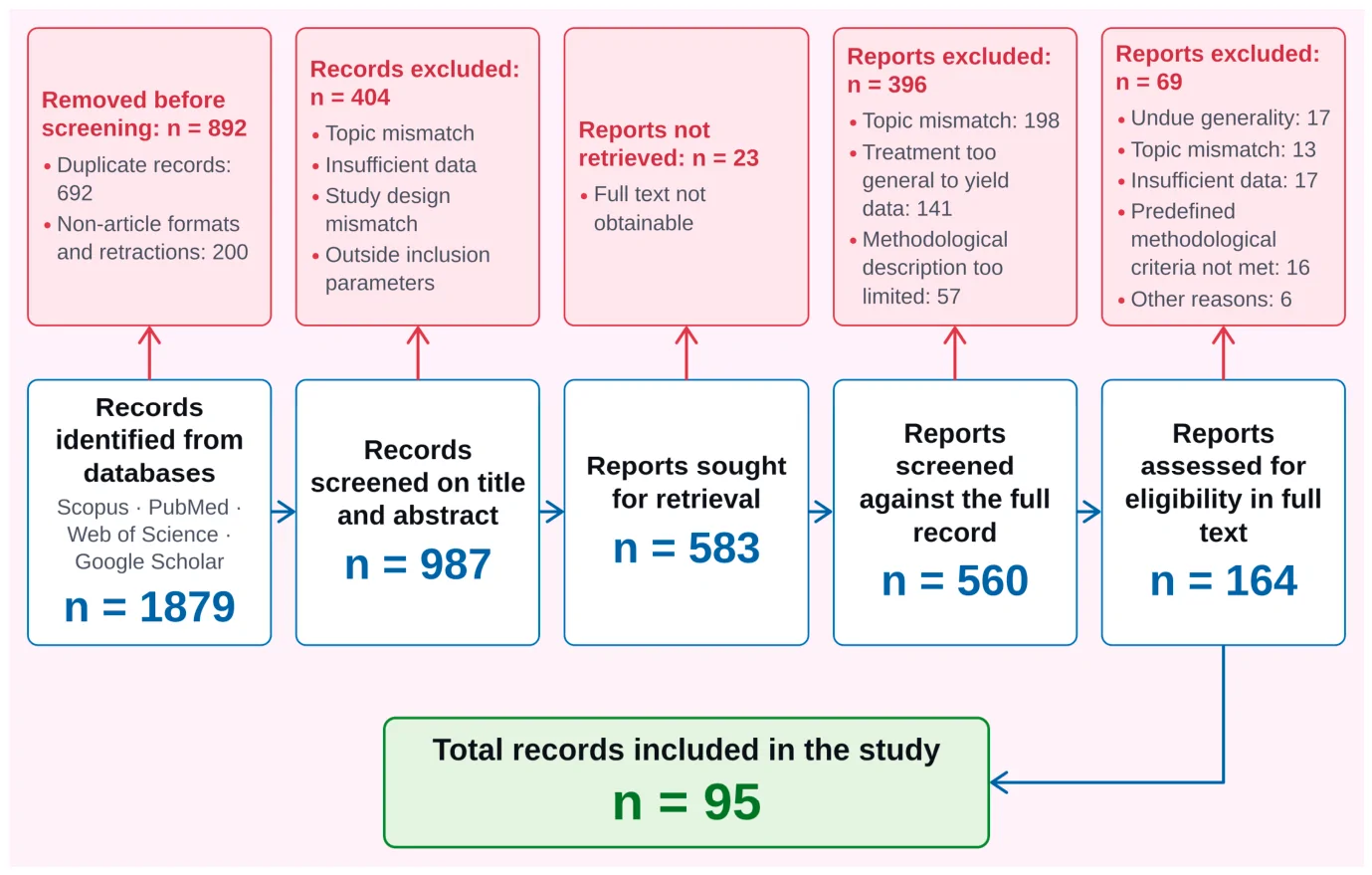
Flow diagram of the literature search, screening, and study selection process, with the reasons for exclusion at each stage. The figure was developed using Python 3.14.

This work was designed as a narrative review based on a systematic literature search, summarising the current preclinical evidence on the anticancer activity of celastrol and its derivatives in breast, ovarian, and cervical cancer, together with the nanotechnology-based delivery systems developed to improve its pharmacological performance. Relevant literature was retrieved from the PubMed, Scopus, Web of Science, and Google Scholar databases. The search was built from three concept blocks combined with the Boolean operator AND: the compound, the tumour type, and the pharmaceutical dimension. The strings executed were, in PubMed, (“celastrol”[All Fields] OR “tripterine”[All Fields] OR “*Tripterygium wilfordii*”[All Fields] OR “Tripterygium”[MeSH Terms]) AND ((“breast neoplasms”[MeSH Terms] OR “breast cancer”[All Fields] OR “ovarian neoplasms”[MeSH Terms] OR “ovarian cancer”[All Fields] OR “uterine cervical neoplasms”[MeSH Terms] OR “cervical cancer”[All Fields]) OR (“derivative*”[All Fields] OR “analogue*”[All Fields] OR “nanoparticle*”[All Fields] OR “nanocarrier*”[All Fields] OR “liposome*”[All Fields] OR “micelle*”[All Fields] OR “drug delivery”[All Fields])); in Scopus, TITLE-ABS-KEY (celastrol OR tripterine OR “*Tripterygium wilfordii*”) AND TITLE-ABS-KEY (“breast cancer” OR “breast neoplasm*” OR “ovarian cancer” OR “ovarian neoplasm*” OR “cervical cancer” OR “uterine cervical neoplasm*” OR derivative* OR analogue* OR nanoparticle* OR nanocarrier* OR liposome* OR micelle* OR “drug delivery”); and in Web of Science, the same expression with TS = in place of TITLE-ABS-KEY. In each of these three databases the compound block was additionally run on its own, as (celastrol OR tripterine OR “*Tripterygium wilfordii*”), so that target-identification and mechanistic studies performed in other models were retrieved; this is the reason that the catalogue of molecular targets draws substantially on work carried out outside the three cancers. Google Scholar does not support field tags, truncation, or nested Boolean logic in the form accepted by the other three databases, so the search was run there in the simplified form celastrol OR tripterine together with “breast cancer” OR “ovarian cancer” OR “cervical cancer” OR nanoparticle OR nanocarrier OR liposome OR micelle OR “drug delivery”, and the first 500 records, sorted by relevance, were screened. The reference lists of the retrieved articles were also screened for additional relevant sources. The searches were last executed in August 2026, and no restriction on publication date or on language of publication was applied. Eligible document types were peer-reviewed reports of primary research. Reviews and book chapters were not eligible as included studies, although such sources are cited where they supply background; conference abstracts, patents, editorials, dissertations, and preprints that had not completed peer review were likewise not eligible. Records flagged in PubMed as retracted publications were excluded. A total of 1879 records were identified. These were imported into Zotero, in which duplicates were removed in two passes, first automatically by matching the digital object identifier and then automatically by matching title, first author, and year of publication; the residual overlap was removed by manual inspection of the remaining titles. Before screening, 692 duplicates and 200 records in non-article formats or subsequently retracted were removed, leaving 987 records that were screened on title and abstract. Of these, 404 were excluded and 583 reports were sought for retrieval. The full text could not be obtained for 23 reports, which are recorded as not retrieved. The remaining 560 reports were examined in a second screening pass against the full record, at which 396 were excluded for the following reasons: the report did not concern celastrol, or did not bear on oncological mechanism, pharmacology, or formulation (*n* = 198); the treatment of the subject was too general to yield extractable data (*n* = 141); and the methodological description was too limited to establish what had been done (*n* = 57). The remaining 164 reports were assessed for eligibility in full text against five predefined methodological criteria, all of which had to be satisfied: celastrol had to be chemically defined, rather than administered as a crude extract or as a component of a multi-herb preparation; the concentration or the dose had to be stated; an untreated or vehicle control had to be present; the biological readout had to be defined and attributable to celastrol rather than to a co-administered agent; and the reported values had to be internally consistent between text, figures, and tables. On this basis 69 reports were excluded, because of undue generality (*n* = 17), topic mismatch (*n* = 13), data insufficient to populate the uniform set of descriptors used in the tables (*n* = 17), failure to meet the five methodological criteria just listed (*n* = 16), or other reasons (*n* = 6). These criteria concern the completeness of reporting and the interpretability of the experiment. They are deliberately not described as an assessment of risk of bias, because no validated instrument was applied and the judgement therefore does not carry the technical meaning that the term has in systematic reviews. Ninety-five studies met all criteria and constitute the evidence base of this review (Figure 1). Screening and eligibility assessment were performed by two of the authors, who assessed the records in duplicate against those criteria; disagreements were resolved by consensus.

Records whose methodological description was too limited to yield the descriptors used in the tables were excluded, and that exclusion will not have operated randomly: it acts against small, early, and briefly reported studies, among which negative results are over-represented. The 95 included studies should therefore be read as the subset of the literature that reports in sufficient detail to be compared on uniform descriptors, and not as the complete body of work on celastrol in these three cancers.

Reports describing molecular mechanisms, cellular targets, concentration or dose ranges, or formulation characteristics were considered eligible. Priority was given to reports providing quantitative pharmacological data, such as half-maximal inhibitory concentration (IC_50_) values, administered doses, and clearly defined molecular endpoints. Information on the phytochemical origin (Table 1) and chemical structure of celastrol, its structural derivatives, its biological activity in women’s cancers, and its incorporation into nanotechnological delivery platforms was extracted and organised into three thematic areas. A uniform set of descriptors was applied across the tables, so that studies could be compared, covering the tested compound, the study model and cell line, the targeted effect, the applied concentration or dose and IC_50_ where available, and the observed activity, mechanism, or benefit. Of the 95 included studies, 24 are listed in Table 2 as direct protein targets, distributed over 25 rows because one study identified three separate targets and one row combines two studies; 39 appear in Table 3 as in vitro and in vivo investigations and 25 in Table 4 as nanotechnological formulations, and a further nine are described individually. Two studies are entered in both Table 2 and Table 3, because they identified a molecular target and characterised the cellular phenotype in the same model, which is why the sum of the table entries exceeds the number of distinct studies. The reference list is larger than this figure because it also contains review, phytochemical, epidemiological, and methodological sources that were not counted as included studies.

## 3. Phytochemical Background

### 3.1. Botanical and Phytochemical Origin

Celastrol is a natural quinone methide triterpenoid belonging to the pentacyclic triterpene family. It was first isolated by Chou and Mei in 1936 from the root bark of *Tripterygium wilfordii* Hook. f. [31]. The species grows in mixed forests, forest margins, woodlands, and scrub at altitudes of 100 to 3500 m, and its native range covers sixteen Chinese provinces, from Jilin and Liaoning in the north to Yunnan, Guangdong, and Guangxi in the south, together with northern Taiwan, Japan, Korea, and northeastern Myanmar [32]. In traditional Chinese medicine, the plant is known as Lei Gong Teng, the Thunder God Vine, and has been used for more than two thousand years, together with other plants of the Celastraceae family, to treat inflammatory and autoimmune diseases such as oedema, gout, joint pain, rheumatoid arthritis, systemic lupus erythematosus, and psoriasis [33,34,35,36]. Although celastrol is also present in other members of the family, *T. wilfordii* remains the most important natural source used in scientific research.

*Tripterygium wilfordii* is a deciduous subshrub or a scrambling, sometimes semiwoody vine 2 to 6 m in height and occasionally reaching 10 m, with slender distal branching that is minutely pubescent when young and becomes glabrous with age. The leaves are usually ovate or rounded-ovate, sometimes oblong or elliptic-ovate, measure 8.6 to 12.5 cm in length and 5.7 to 8.9 cm in width, and have an entire or, less often, crenulate margin and a short to long acuminate apex. Flowering occurs from May to October and fruiting from August to November. The flowers are small, 4 to 6 mm across, whitish, greenish, or yellow-green, and are borne in large thyrses composed of several to dozens of thyrselets that measure 12.5 to 23.6 cm and may reach 38 cm in length. The fruit is a three-winged samara 1.3 to 1.9 cm long, green or greenish brown and sometimes pink to pinkish purple at maturity, containing a compressed, three-angled seed about 5 mm long [32]. The roots and root bark are of greatest pharmacological importance, constituting a rich source of biologically active secondary metabolites, including celastrol, whereas the compound occurs in much smaller quantities in the above-ground parts. More than 500 metabolites have been identified in *T. wilfordii*, including sesquiterpenes, diterpenes, triterpenes, alkaloids, flavonoids, lignans, and glycosides, among them quinone methide triterpenoids such as celastrol [33,36].

Beyond *T. wilfordii*, celastrol has been reported in several other species of the Celastraceae family. These include *Tripterygium regelii* Sprague & Takeda, *Celastrus orbiculatus* Thunb. [37,38], *C. hypoleucus* (Oliv.) Warb. ex Loes. [39], *C. paniculatus* Willd. and *C. scandens* L., as well as *Maytenus truncata* (Nees) Reissek (syn. *M. ilicifolia* Mart. ex Reissek) and *M. macrocarpa* (Ruiz & Pav.) Briq. [40,41,42,43,44,45,46,47]. Roots and root bark are the most frequent sites of accumulation, but celastrol has also been obtained from stem bark and from stem bark exudate [40,43,44]. *Maytenus senegalensis* is sometimes listed among celastrol-containing species, but a recent review cataloguing all 118 metabolites reported from that plant identifies pristimerin, and not celastrol, as its quinone methide triterpene [48]. Names and authorities for these taxa were verified in Plants of the World Online [49] and the International Plant Names Index [50]. Molecular analysis of Chinese *Celastrus* and *Tripterygium* confirms the close relationship between the two genera and the resulting overlap in their secondary metabolite profiles [51]. This distribution across genera provides alternative botanical sources, although the yield and availability of celastrol from plant material remain limited. Examples of plant species reported to contain celastrol, together with the plant part in which it accumulates, are summarised in Table 1.

**Table 1:** Examples of plant species reported to contain celastrol and the plant part in which it accumulates.

Species	Genus	Occurrence of Celastrol	References
*Tripterygium wilfordii* Hook. f. (syn. *T. hypoglaucum* (H. Lev.) Hutch.)	*Tripterygium*	root bark, roots (richest source)	[35,52]
*Celastrus orbiculatus* Thunb.	*Celastrus*	roots, root bark	[37,38]
*Celastrus hypoleucus* (Oliv.) Warb. ex Loes.	*Celastrus*	roots (f. *argutior* Loes.)	[39]
*Maytenus truncata* (Nees) Reissek (syn. *M. ilicifolia* Mart. ex Reissek)	*Maytenus*	root bark (detected in 2 of 5 morphological types)	[42]
*Maytenus macrocarpa* (Ruiz & Pav.) Briq.	*Maytenus*	stem bark, stem bark exudate	[40,41,43]
*Tripterygium regelii* Sprague & Takeda	*Tripterygium*	stem bark	[44]
*Celastrus paniculatus* Willd.	*Celastrus*	roots (in vitro adventitious and hairy root cultures)	[45]
*Celastrus scandens* L.	*Celastrus*	plant part not specified	[46]

### 3.2. Chemical Structure

Celastrol belongs to the pentacyclic triterpenoid group and, more specifically, to the quinone methide triterpenoid subclass. Triterpenes are a large group of natural organic compounds composed of thirty carbon atoms and are widely distributed in plants. Chemically, celastrol is a derivative of the friedelane skeleton composed of five fused carbon rings, designated A to E, and is characterised by a highly reactive quinone methide system spanning rings A and B, which is responsible for a significant part of its biological activity [52,53]. The full structure was assigned only in 1962, on the basis of combined chemical and nuclear magnetic resonance evidence [31].

Celastrol has the molecular formula C_29_H_38_O_4_ and a molar mass of 450.61 g/mol, and it is a red powder with a melting point of 219 to 230 °C [54]. Its systematic name according to IUPAC nomenclature is (2R,4aS,6aR,6aS,14aS,14bR)-10-hydroxy-2,4a,6a,6a,9,14a-hexamethyl-11-oxo-1,3,4,5,6,13,14,14b-octahydropicene-2-carboxylic acid [55]. The compound absorbs in the ultraviolet and visible range with maxima at 253 and 424 nm [54], and the visible band at 424 nm in ethanol is routinely used to quantify celastrol in formulation studies [56]. It is sparingly soluble in water and soluble in dimethyl sulfoxide and ethanol [54]. The molecule contains a carboxyl group at C-20 and six methyl groups distributed within the triterpene skeleton, and a hydroxyl group at C-3 influences its physicochemical properties. The carboxyl carbon is itself C-29 and is attached to C-20, so that the same group appears in the literature as the C-20 carboxyl group and as the C-29 carboxylic acid; this review uses the former throughout. The A ring carries a conjugated system of double bonds and carbonyl groups that confers electrophilic properties, in particular at C-2 of the A ring and C-6 of the B ring, both of which are susceptible to nucleophilic attack [53].

**Figure 2:**
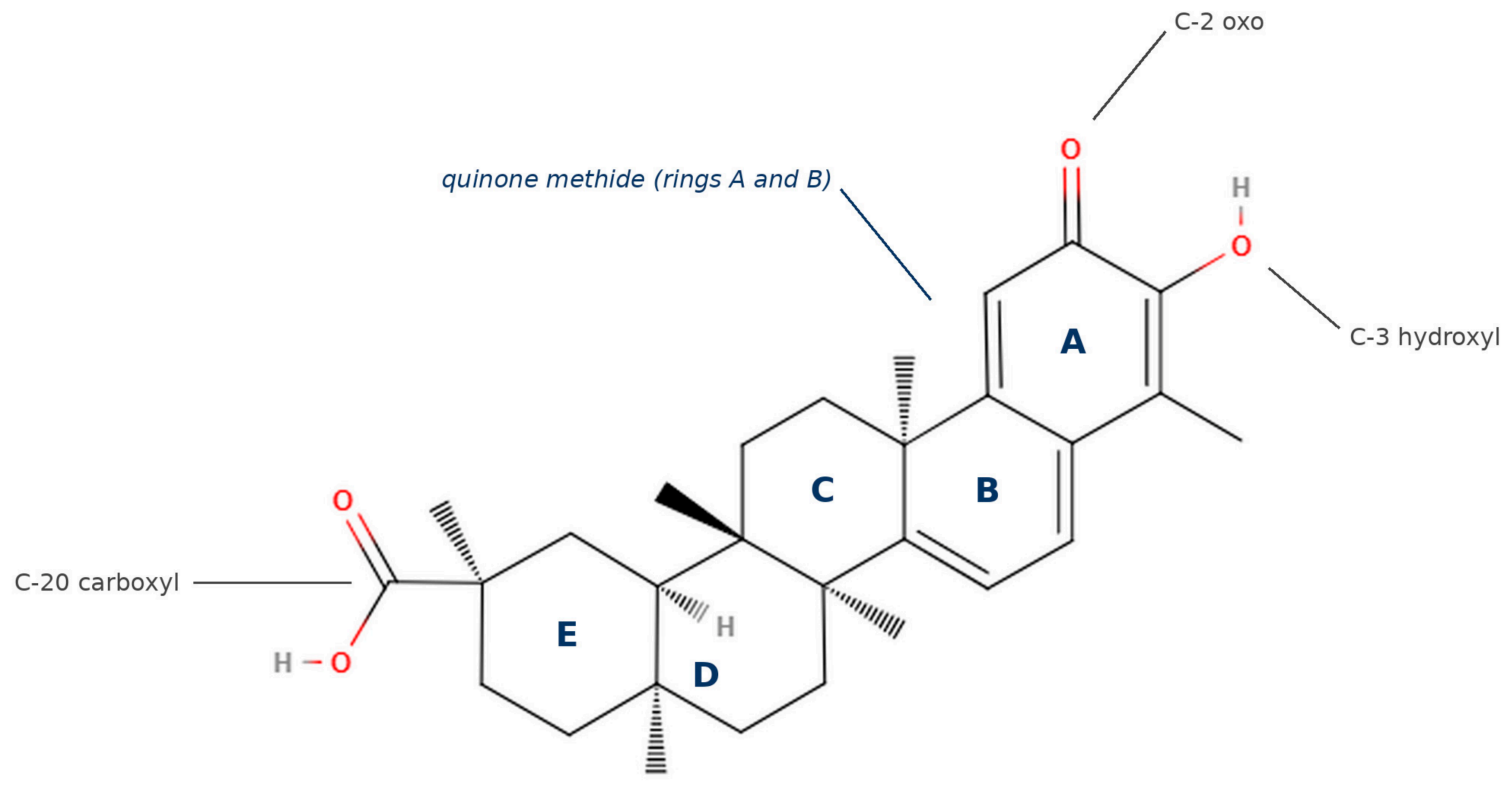
Chemical structure of celastrol showing the friedelane skeleton with rings lettered A to E, the quinone methide moiety spanning rings A and B, the C-2 oxo and C-3 hydroxyl groups that carry the electrophilic centres, and the C-20 carboxyl group at which most semisynthetic modification has been performed. The structure was developed using the MolView software (https://molview.com/, accessed on 6 August 2026). Oxygen atoms are shown in red and hydrogen atoms in grey.

The electrophilic quinone methide fragment is the key structural element responsible for the pharmacological activity of celastrol. It enables the formation of reversible or irreversible covalent adducts, known as Michael adducts, with the thiol groups of cysteine residues of many regulatory proteins. This mechanism leads to modulation of numerous signalling pathways, including NF-κB [53,57], the heat shock proteins Hsp90 and Hsp70 as well as the transcription factor HSF1 [53], the PI3K/Akt/mTOR axis [10,58], and the transcription factor Nrf2 [59], which together govern inflammatory processes, oxidative stress, and cell proliferation. The chemical structure of celastrol, with the A to E ring lettering and the principal functional groups indicated, is presented in Figure 2.

### 3.3. Derivatives of Celastrol

Pharmacological interest in celastrol is accompanied by well-recognised liabilities, namely very low aqueous solubility, limited bioavailability, and a narrow therapeutic window, which have motivated extensive medicinal chemistry efforts to obtain semisynthetic derivatives with improved drug-like properties [10,60]. Two positions of the molecule are particularly amenable to modification: the carboxyl group at C-20, which has been derivatised with amino acids, amines, and triazoles to give series of compounds screened against a panel that included HeLa cervical cancer cells [61], and the electrophilic A and B ring system that carries the quinone methide moiety. Modifications at these positions have been used to modulate solubility, potency, selectivity for tumour cells over normal cells, and, in some cases, the ability to engage specific molecular targets [10].

In breast cancer models, structural optimisation has produced analogues that outperform the parent compound. A celastrol pyrazine derivative bearing a C-20 urea moiety was more potent than celastrol against MCF-7 cells, with an IC_50_ of 1.31 μM compared with 2.23 μM for the parent compound, and it induced autophagy through the regulation of LC3 A/B, p62, and Beclin-1 while showing favourable selectivity over a normal cell line [62]. Proteolysis-targeting chimeras built on the celastrol scaffold have extended this approach to targeted protein degradation. A celastrol-based degrader simultaneously promoted the degradation of GRP94, CDK1, and CDK4 and was more potent than celastrol against triple-negative breast cancer cells and in a 4T1 model [59], whereas the rigid-linker derivative YX-112 degraded CHEK1 and PIK3R2, downregulated CDK4 and phosphorylated AKT, and achieved an IC_50_ of 0.32 μM against MDA-MB-231 cells, roughly three-fold lower than that of celastrol, together with improved selectivity [58].

In ovarian cancer, modifications of the C-20 carboxyl group have been especially productive. Cinnamamide-substituted analogues, in particular the 3,4,5-trimethoxycinnamamide derivative [63], and piperazine-substituted analogues [64] and the piperazine conjugate Cel-N [65], were consistently more potent than celastrol, with submicromolar IC_50_ values against SKOV3, A2780, and OVCAR3 cells and greater selectivity over normal ovarian epithelial cells. These derivatives suppressed ovarian cancer stem cell markers, inhibited spheroid formation, induced apoptosis through caspase activation and mitochondrial membrane depolarisation, and attenuated STAT3 signalling, whereas the effect on the upstream kinases JAK1 and JAK2 was only marginal. Cel-N additionally showed marked antitumour activity with low systemic toxicity in a xenograft model [65]. Two further series of C-20 esters and carbamates point in the same direction and, unlike most of the work above, were profiled against breast as well as ovarian lines. In the ester series, compound **24** was twice as potent as celastrol against SKOV3 cells, with a half-maximal inhibitory concentration of 0.56 μmol/L compared with 1.16 μmol/L, was about six times more active in SKOV3 cells than in non-tumour BJ fibroblasts, and retained activity against SK-BR-3 and MDA-MB-231 cells at 0.78 and 1.15 μmol/L [66]. In the companion carbamate series, compound **18** reached 0.54 μmol/L against SKOV3 cells with an approximately seven-fold margin over BJ fibroblasts and weakened colony formation at twice that concentration, whereas compound **9** was more potent across the panel but lost the selectivity margin [67]. Cervical cancer is represented by a series of C-20 amino acid esters in which the two most active compounds lowered the half-maximal inhibitory concentration against HeLa cells from 0.947 μmol/L for celastrol to 0.371 and 0.237 μmol/L, with a comparable gain against A549 cells, while aromatic amino acid substitution abolished the improvement [68].

## 4. Molecular Targets and Signalling Networks of Celastrol

### 4.1. The Quinone Methide as a Cysteine-Directed Electrophile

The pharmacological behaviour of celastrol is governed by a single structural feature. The quinone methide moiety spanning rings A and B is a Michael acceptor, and it reacts with the thiolate form of cysteine residues in proteins. Every attempt to define the pharmacophore has converged on this conclusion. In a systematic study of which positions of the molecule tolerate modification, conversion of the C-20 carboxylic acid into amides and long-chain analogues preserved the capacity to induce the heat shock response and the antioxidant response and to inhibit Hsp90 activity. The A ring enol position, by contrast, proved refractory to synthetic modification and was judged unsuitable for probe attachment [69]. The same work produced the first biotinylated celastrol probes and used them in extracts of human Panc-1 cells to retrieve annexin II, eEF1A, and β-tubulin, providing early evidence that the compound engages a set of proteins rather than a single receptor [69]. Functional confirmation of the electrophilic requirement came from work in glioblastoma cells, in which 6-N-acetylcysteinyldihydrocelastrol, the Michael adduct formed between celastrol and N-acetylcysteine, had no effect on p62 or on Hsp72 expression, indicating a thiol-dependent mechanism [70].

Covalent reactivity alone would predict indiscriminate labelling of the accessible cysteine proteome, but that is not what is observed. Work using Nur77 as a model target showed that celastrol is first positioned by specific noncovalent contacts that orient the quinone methide next to the reactive cysteine, and that the resulting adduct is reversible rather than permanent [71]. Two consequences follow, and both are relevant to the therapeutic prospects of the compound. First, the polypharmacology of celastrol is an intrinsic property of its chemistry rather than an artefact of incomplete mechanistic study, and selectivity therefore has to be engineered through the noncovalent recognition element rather than through the reactive warhead. Second, because adduct formation is reversible, target engagement is governed by residence time rather than by irreversible inactivation. Celastrol therefore occupies a pharmacological category distinct from classical covalent inhibitors, a difference that helps to explain why the compound behaves so differently across concentration ranges. Reviews of the druggability of celastrol have approached the same problem from the perspective of its physicochemical liabilities, describing low aqueous solubility, low oral bioavailability, and dose-dependent toxicity affecting the liver, kidney, heart, and reproductive system [72,73]. The combination of a narrow therapeutic window with severe adverse effects has been identified as the principal obstacle to development [74].

### 4.2. Direct Protein Targets Identified by Chemical Proteomics

The catalogue of direct celastrol targets has grown from isolated observations into a body of evidence generated by activity-based protein profiling, thermal proteome profiling, and structural biology. The strength of the underlying evidence varies considerably between targets, and the discussion below is ordered accordingly. Table 2 summarises these proteins, grouped into three evidence categories, and states for each the residue involved where it has been mapped, the method of demonstration, the model system in which the identification was performed, and the cellular consequence.

Because the term direct target is applied loosely in this literature, the evidence behind every assignment made in this review is graded on a single explicit scale. The weakest level is observation or correlation, where a protein, a transcript, a metabolite, a reactive oxygen species signal, or a measure of mitochondrial function is seen to change after exposure to celastrol. A change of that kind identifies a consequence of treatment and establishes neither physical engagement nor causal necessity. The second level is pharmacological perturbation, in which an inhibitor or an activator of the candidate reproduces or abolishes the effect of celastrol; this raises the level of evidence but inherits the selectivity limitations of the tool compound. The third is genetic validation, by knockdown, knockout, overexpression, or expression of a drug-resistant mutant, which tests whether the protein is required for the effect. The fourth is biochemical target validation, in which celastrol is shown to inhibit or to modify the purified protein. The fifth and highest is direct binding evidence, from a structure of the covalent adduct, from a biophysical measurement of affinity, or from mapping of the modified residue together with mutagenesis of that residue. In what follows, the term direct target is reserved for proteins supported at the fifth level, the term functional target for those supported at the third or fourth, and the term putative target for those supported only at the first or second; where a claim rests on a lower level than the vocabulary might suggest, this is stated at the point at which the claim is made.

The earliest molecular target to be assigned was the proteasome. Celastrol preferentially inhibits the chymotrypsin-like activity of the purified 20S proteasome, with a half-maximal inhibitory concentration of 2.5 μmol/L, and of the cellular 26S proteasome at 1 to 5 μmol/L. In prostate cancer cells the inhibition is followed by accumulation of ubiquitinated proteins and of the natural proteasome substrates IκBα, Bax, and p27, by suppression of androgen receptor expression, and by apoptosis [75]. Administration of 1 to 3 mg/kg per day intraperitoneally for up to 31 days inhibited the growth of PC-3 xenografts by 65% to 93%, with proteasome inhibition confirmed in the tumour tissue itself [75]. A second study using computational prediction followed by enzymatic assay with recombinant protein identified the catalytic β1 subunit as a direct point of attack. Of the three catalytic subunits, only the β1-mediated peptidyl-glutamyl activity was significantly inhibited, at a concentration as low as 0.2 μmol/L [76]. The two reports therefore assign different subunit preferences, and the question of which catalytic activity is engaged first has not been settled.

The IκB kinase complex provides the clearest example of residue-level target definition. Celastrol inhibits IKK activity and the constitutively active form of IKKβ in a dose-dependent manner, blocks the phosphorylation and degradation of IκBα induced by lipopolysaccharide, tumour necrosis factor α, or phorbol ester. These effects occur without any change in the DNA binding activity of AP-1 and without suppression of NF-κB activation driven by RelA overexpression [77]. Mutation of cysteine 179 in the activation loop of IKKβ abolishes sensitivity to the compound. This residue is therefore the site of covalent modification and the point at which celastrol enters the NF-κB network [77].

The chaperone machinery is engaged through a mechanism unlike that of classical Hsp90 inhibitors. Celastrol disrupts formation of the Hsp90 and Cdc37 complex, an effect not produced by geldanamycin, and it inhibits the ATPase activity of Hsp90 without blocking ATP binding. Proteolytic fingerprinting localised the interaction to the C-terminal domain, indicating allosteric rather than competitive regulation [78]. Solution nuclear magnetic resonance analysis of the human complex, which forms with a dissociation constant of 1.2 μmol/L, placed the binding event on Cdc37 itself and attributed disruption to cysteine-dependent inactivation of the cochaperone [79]. A discrete binding pocket within the Hsp90 dimer was later defined by molecular docking and validated by isothermal titration calorimetry; mutation of tyrosine 493 and lysine 546 abolished celastrol binding and simultaneously weakened the association between Hsp90 and Cdc37 [80]. These findings are not mutually exclusive and together indicate that the complex is destabilised from more than one direction. In parallel with disrupting the chaperone complex, celastrol activates heat shock factor 1, inducing DNA binding and hyperphosphorylation of the factor and expression of Hsp70, Hsp40, and Hsp27, with kinetics resembling those of heat stress [81].

Peroxiredoxins have emerged as a target family rather than as a single protein, and the three independent lines of evidence are mutually reinforcing. A high-resolution crystal structure revealed the covalent binding mode of celastrol to peroxiredoxin 1 in colorectal cancer, and structure-guided design yielded the derivative 19-048 with improved potency against PRDX1 and selectivity over PRDX2 to PRDX6. Antitumour efficacy was significantly diminished in xenografts established from PRDX1 knockdown cells, although the same knockdown sensitised the cells in vitro [82]. Independently, proteome microarray screening combined with surface plasmon resonance, isothermal titration calorimetry, and molecular simulation identified peroxiredoxin 2 as a direct binding partner in gastric cancer. There, inhibition of its enzymatic activity, rather than of its expression, raised intracellular reactive oxygen species and produced endoplasmic reticulum stress, mitochondrial dysfunction, and apoptosis in vitro, as well as tumour growth inhibition in mice [83]. Activity-based protein profiling with a clickable celastrol probe, followed by cellular thermal shift assay, subsequently mapped adducts to the catalytic cysteines of PRDX1, PRDX2, PRDX4, and PRDX6, and additionally identified haem oxygenase 1, with ferroptosis as the functional readout. This work was performed in activated hepatic stellate cells in a model of hepatic fibrosis rather than in a tumour [84]. Peroxiredoxin 2 was also recovered in an unbiased screen combining cellular thermal shift assay with pulse proteolysis in colorectal cancer cells; the screen nominated eight binding proteins in total, namely EEF2, STIP1, GAPDH, FLNA, SETSIP, GANAB, TXNDC17, and PRDX2 [85].

Metabolic enzymes constitute a further group. Activity-based protein profiling in models of endotoxaemia identified pyruvate kinase M2 as a covalent target adducted at cysteine 424, with inhibition of pyruvate kinase activity and suppression of aerobic glycolysis; the same study mapped adduct formation at cysteine 106 of high mobility group box 1 protein and at cysteine residues of lactate dehydrogenase A [86]. The single most relevant study for the present review applied the same technology in breast cancer. Celastrol inhibited the proliferation of MDA-MB-231 and MCF-7 cells with half-maximal inhibitory concentrations of 2.15 and 2.29 μmol/L, respectively, and suppressed tumour growth in syngeneic allograft and cell-line-derived xenograft models at 5, 2, and 2 mg/kg given intraperitoneally after tumour formation [87]. Activity-based protein profiling identified the mitochondrial isocitrate dehydrogenases IDH2 and IDH3A as direct targets. Their inhibition caused accumulation of α-ketoglutarate, enhanced the interaction between DPYSL2 and IDH3A, and promoted mitochondrial accumulation of DPYSL2, which in turn inactivated JAK/STAT signalling and culminated in ferroptosis and apoptosis [87].

Two recent studies extended the target list using strategies that assess target engagement in a different way. Label-free thermal proteome profiling in chronic myeloid leukaemia carrying the BCR-ABL T315I mutation nominated twelve high-confidence targets and supported engagement of YY1 and HMCES by in situ probe labelling and molecular docking, with biolayer interferometry performed for YY1 [88]. In a degradation-based profiling strategy, celastrol was converted into proteolysis-targeting chimeras and the resulting degradation events were read out by quantitative proteomics and immunoprecipitation coupled to mass spectrometry. The approach confirmed IKKβ, phosphoinositide 3-kinase α, and CIP2A as targets and identified checkpoint kinase 1, O-GlcNAcase, and ERCC6L as new ones [89]. The method reports degradation rather than binding; target engagement is therefore inferred from the consequence of chimera-mediated recruitment. The strategy is of particular interest here because it connects the mechanistic account directly to the celastrol-based proteolysis-targeting chimeras described above [58,59,89]. A separate line of evidence concerns transcription factors themselves. Celastrol and a set of celastrol-inspired triterpenoids disrupt c-Myc and Max heterodimers directly, with half-maximal inhibitory concentrations of 10 to 15 μmol/L for the parent compound in electrophoretic mobility shift assays and four- to six-fold greater potency for the best analogues, together with selective inhibition of a Myc-responsive promoter in HeLa cells; nuclear magnetic resonance showed no chemical shift perturbation in either protein alone at concentrations up to 125 μmol/L, and large perturbations once the heterodimer had been assembled, so that the dimer rather than either subunit provides the binding site. The recombinant proteins used in that work carried no free cysteines, which means that this particular engagement does not proceed through the cysteine-directed chemistry that governs the remainder of this section [90].

Finally, several targets relevant to immune function have been defined at residue level. Celastrol binds covalently to cysteine 170 of COMMD3 and dissociates the COMMD3 and COMMD8 complex that serves as a signalling adaptor for chemoattractant receptors, with a half-maximal inhibitory concentration for complex dissociation of 0.74 μmol/L measured by bioluminescence resonance energy transfer. Mice expressing a celastrol-resistant mutant of the complex were refractory to the compound, the most rigorous genetic validation available for any celastrol target [91]. In melanoma, assays combining molecular docking, surface plasmon resonance, and small interfering RNA nominated the tyrosine phosphatase SHP2 as the target through which celastrol restores major histocompatibility complex class I expression. The measured affinity was weak, however, with a dissociation constant of 37.93 μmol/L [92].

Three qualifications should be attached to this catalogue. Mapping to a numbered cysteine residue exists for only a minority of the proteins listed, namely cysteine 179 of IKKβ, cysteine 173 of PRDX1, cysteine 424 of PKM2, cysteine 106 of HMGB1, and cysteine 170 of COMMD3. Many of the remaining assignments rest on a single study without orthogonal validation. Most importantly, with the exception of the mitochondrial isocitrate dehydrogenases identified in breast cancer [87], of HSDL2 in MDA-MB-231 cells [93], and of the competitive target profiling performed in HeLa cells [94], target identification has been carried out in tumour types outside the scope of this review. Extrapolation to breast, ovarian, and cervical cancer therefore remains an assumption rather than a demonstrated fact.

**Table 2:** Protein targets of celastrol, grouped into three categories according to the kind and the strength of the evidence on which each assignment rests. Category 1 comprises directly validated molecular targets, for which the modified residue has been mapped and the assignment confirmed by site-directed mutagenesis, by genetic validation in vivo, or by a structure of the covalent adduct. Category 2 comprises strongly supported functional targets, for which physical binding or direct inhibition of the purified protein has been measured, but the modified residue has not been mapped or no independent genetic or mutational test has been performed. Category 3 comprises putative or inferred targets, nominated by a functional or degradation readout, by an unbiased thermal-shift or pulse-proteolysis screen, or by computational docking, without a direct measurement of binding to the individual protein; entries in this category should not be read as established targets. The evidence-context column states the model system in which the identification was performed and whether that system lies within the three cancers reviewed here. The categories grade the evidence for target engagement and not the biological importance of the protein.

Protein or Complex	Residue Modified	Method of Demonstration	Evidence Context	Cellular Consequence	Ref.
Category 1. Directly validated molecular targets. The modified cysteine has been mapped to a numbered residue and the assignment confirmed by site-directed mutagenesis of that residue, by genetic validation in vivo, or by a crystal structure of the covalent adduct.					
PRDX1	Cys173	X-ray crystal structure of the covalent adduct; structure-guided derivative 19-048; efficacy diminished in PRDX1 knockdown xenografts	Colorectal cancer; outside the three cancers reviewed here	Loss of peroxidase activity, elevated ROS, suppression of colorectal tumour growth	[82]
IKKβ	Cys179 (activation loop)	Cys179Ala mutant abolishes sensitivity; kinase assays with constitutively active IKKβ	Cell-free kinase assays and inflammatory cell models; outside	Block of IκBα phosphorylation and degradation, shutdown of NF-κB signalling	[77]
COMMD3 (COMMD3/8 complex)	Cys170	Covalent binding; IC_50_ 0.74 μmol/L for complex dissociation by BRET; knock-in mice expressing a celastrol-resistant mutant	Murine B cells and autoimmunity models; outside; the only target validated genetically in vivo	Dissociation of the complex, impaired chemoattractant receptor signalling, reduced B cell migration	[91]
PKM2	Cys424	Activity-based protein profiling with clickable probe, CETSA, SPR, point mutagenesis	RAW264.7 macrophages and mouse models of endotoxaemia and sepsis; non-neoplastic	Inhibition of pyruvate kinase activity, suppression of aerobic glycolysis	[86]
HMGB1	Cys106	Activity-based protein profiling, point mutagenesis	RAW264.7 macrophages and mouse models of endotoxaemia and sepsis; non-neoplastic	Reduced secretion of interleukin 1 beta	[86]
Category 2. Strongly supported functional targets. Physical binding, or direct inhibition of the purified protein, has been measured by structural, biophysical, or affinity-based methods, but the modified residue has not been mapped, or no independent genetic or mutational test of the assignment has been performed.					
Lactate dehydrogenase A	cysteine residues, not numbered	Activity-based protein profiling in models of endotoxaemia, in the same experiment that mapped PKM2 and HMGB1	RAW264.7 macrophages and mouse models of endotoxaemia and sepsis; non-neoplastic	Adduct formation on cysteine residues, consistent with the suppression of aerobic glycolysis reported in the same model	[86]
Hsp90 (dimer interface)	Tyr493, Lys546 (binding pocket, not a cysteine)	Molecular docking with isothermal titration calorimetry on point mutants	Recombinant protein and point mutants; cell-free	Loss of celastrol binding and weakened association with Cdc37	[80]
Hsp90–Cdc37 complex	not mapped	GST pull-down, ELISA, ATPase assay, proteolytic fingerprinting of the C-terminal domain	Recombinant proteins; cell-free	Allosteric inhibition of ATPase activity without blocking ATP binding; destabilisation of kinase clients	[78]
Cdc37	cysteine-dependent	1H,15N-HSQC solution NMR of the human complex	Recombinant human complex; cell-free	Inactivation of the cochaperone and disruption of the complex	[79]
PRDX2	catalytic cysteine	Proteome microarray, surface plasmon resonance, isothermal titration calorimetry, molecular simulation	Gastric cancer; outside	Inhibition of enzymatic activity, ROS accumulation, ER stress and mitochondrial dysfunction	[83]
PRDX1, PRDX2, PRDX4, PRDX6 and HO-1	catalytic cysteines	Activity-based protein profiling with clickable probe and LC-MS/MS, CETSA; hepatic stellate cells, not a tumour model	Colorectal cancer; outside the three cancers reviewed here	Lipid peroxidation and ferroptosis	[84]
Mitochondrial IDH2 and IDH3A	not reported	Activity-based protein profiling in MDA-MB-231 and MCF-7 breast cancer cells	Breast cancer (MDA-MB-231, MCF-7); within the cancers reviewed here	α-Ketoglutarate accumulation, DPYSL2 sequestration, JAK/STAT inactivation, ferroptosis and apoptosis	[87]
HSDL2	not mapped	Activity-based profiling with a clickable celastrol probe and mass-spectrometric identification, competitive pull-down in which excess celastrol displaced the probe, and CETSA, in MDA-MB-231 triple-negative breast cancer cells	Breast cancer (MDA-MB-231); within the cancers reviewed here	Promotion of apoptosis in triple-negative breast cancer cells; together with the mitochondrial isocitrate dehydrogenases, one of only two direct targets identified in a breast cancer model	[93]
20S and 26S proteasome; catalytic subunit β1	not mapped	Enzymatic assay with purified 20S proteasome (chymotrypsin-like activity preferred) and with recombinant β1 subunit (peptidyl-glutamyl activity preferred); accumulation of ubiquitinated proteins	Purified enzyme, recombinant β1 subunit, and prostate cancer cells and xenografts; outside	Stabilisation of IκBα, Bax and p27, cell cycle arrest, proteotoxic stress	[75,76]
Nur77 (NR4A1)	reactive cysteine in the ligand pocket	Model system for reversible covalent binding preceded by specific noncovalent contacts	Model system for the binding mode; no cellular phenotype established	Demonstration of the binding mode rather than of a phenotype	[71]
SHP2 (PTPN11)	not mapped	Molecular docking, surface plasmon resonance (KD 37.93 μmol/L), small interfering RNA	Melanoma; outside; affinity weak relative to the concentrations used in cellular work	Restoration of MHC class I expression through JAK/STAT1, enhanced CD8+ T cell killing	[92]
Interleukin 2	not mapped	Screen for inhibitors of the IL-2 and CD25 interaction; binding to IL-2 and not to CD25	B16F10 melanoma in immunocompetent mice, with loss of effect in T cell-deficient mice; outside	Impaired IL-2-dependent STAT5 phosphorylation, increased intratumoural CD8+ T cells	[95]
YY1 and HMCES	not mapped	Label-free thermal proteome profiling (MS-CETSA), in situ probe labelling and docking; biolayer interferometry for YY1 only	Chronic myeloid leukaemia carrying BCR-ABL T315I; outside; binding measured for YY1 only	DNA damage and cell death in BCR-ABL T315I-mutant leukaemia	[88]
Hsp90 as scaffold for NLRP3	not mapped	Molecular docking, biolayer interferometry (Octet RED96), coimmunoprecipitation	Fibroblast-like synoviocytes and collagen-induced arthritis in mice; non-neoplastic	Disruption of the Hsp90 and NLRP3 interaction and inflammasome inhibition	[96]
c-Myc–Max heterodimer	Not mapped	Electrophoretic mobility shift assay with purified proteins, NMR showing that celastrol binds the assembled heterodimer and neither protein alone, and a Myc-responsive reporter in HeLa cells	Purified proteins and a reporter assay in HeLa cervical cancer cells; partly within; binding requires the assembled heterodimer	Direct disruption of c-Myc–Max heterodimers and inhibition of Myc-driven transcription; IC_50_ 10 to 15 μmol/L for celastrol, four- to six-fold lower for the most potent celastrol-inspired analogues	[90]
Category 3. Putative or inferred targets. The protein was nominated by a functional or degradation readout, by an unbiased thermal-shift or pulse-proteolysis screen, or by computational docking, without a direct measurement of binding to the individual protein. Entries in this category should not be read as established targets of celastrol.					
EEF2, STIP1, GAPDH, FLNA, SETSIP, GANAB, TXNDC17, PRDX2	not mapped	Cellular thermal shift assay with pulse proteolysis, two-dimensional electrophoresis and MALDI-TOF	Colorectal cancer; outside; consequences not established for the individual proteins	Not individually established	[85]
Target set nominated by competitive profiling in HeLa cells	not mapped	Quantitative competitive chemical proteomics in HeLa cervical cancer cells	Cervical cancer (HeLa); within; the only target identification performed in a cervical cancer model	The only target identification carried out in a cervical cancer model; consequences of engagement not established for the individual proteins nominated	[94]
Annexin II, eEF1A, β-tubulin	not mapped	Affinity capture with biotinylated celastrol probes in Panc-1 extracts	Pancreatic cancer cell extracts; outside; early probe-capture experiment without orthogonal validation	Not individually established	[69]
HSF1 (activation, not inhibition)	not applicable	HSF1 DNA binding and hyperphosphorylation; induction of Hsp70, Hsp40 and Hsp27	HeLa cervical and MCF7 and BT474 breast cancer lines among others; a functional readout of the heat shock response rather than a target identification	Heat shock response with cytoprotective potential	[81]
IKKβ, PI3Kα, CIP2A, CHK1, OGA, ERCC6L	not mapped	Degradation-based protein profiling using celastrol-derived PROTACs with quantitative proteomics and IP-MS; reports degradation rather than binding	Cell-free kinase assays and inflammatory cell models; outside	Targeted degradation of the corresponding proteins	[89]

### 4.3. Signalling Networks Engaged in Breast, Ovarian, and Cervical Cancer

The evidence presented in this section stands on a different footing from the assignment of direct molecular targets, and the distinction is maintained throughout rather than left to the reader. With two exceptions, namely the NF-κB axis, where the upstream covalent event has been localised to cysteine 179 of IKKβ, and the JAK and STAT axis, where the mitochondrial isocitrate dehydrogenases supply a mapped upstream target in breast cancer, the evidence in this section consists of changes in the abundance, the phosphorylation state, or the subcellular localisation of pathway components following exposure to celastrol. Observations of this kind establish that a pathway responds to the compound. They do not establish that the pathway is engaged directly, and they do not establish that it is required for the cellular outcome, unless the study in question also perturbed the pathway independently by pharmacological or genetic means. Where such a causal test was performed, it is identified explicitly; where the evidence is associative, the text says so.

#### 4.3.1. NF-κB and Inflammatory Signalling

The suppression of NF-κB signalling is the most consistently reported effect of celastrol across tumour types, and its molecular basis is the covalent modification of cysteine 179 of IKKβ described above [77]. Downstream consequences have been documented in all three cancers considered here. Celastrol potentiates tumour necrosis factor-induced apoptosis and suppresses invasion by inhibiting NF-κB-regulated gene products and TAK1-mediated NF-κB activation [57], inhibits breast cancer cell invasion through suppression of NF-κB-mediated matrix metalloproteinase 9 expression [22], and blocks migration and invasion of ovarian cancer cells through the same pathway [97]. In triple-negative breast cancer, the inflammatory arm of this axis has been dissected further, with attenuation of interleukin 1β expression [98] and interference with immune escape [99]. The pathway is also a point of convergence with other networks, because Notch1 is an upstream regulator of NF-κB family members. In U937 cells, celastrol inhibited growth with a half-maximal inhibitory concentration of 6.21 ± 0.242 μmol/L at 24 h, arrested cells in G0/G1, downregulated Notch1 at both messenger RNA and protein levels, and redistributed the p65 subunit from the nucleus to the cytoplasm [100].

#### 4.3.2. PI3K/Akt/mTOR and the AMPK Axis

Inhibition of PI3K and Akt signalling accompanies celastrol treatment in essentially every model examined, and in triple-negative breast cancer it occurs together with oxidative stress and mitochondrial dysfunction [18,101]. Two mechanistic refinements deserve emphasis. The first is that celastrol does not merely inhibit the kinase cascade but removes one of its components: it induces ubiquitin-dependent degradation of mTOR in breast cancer cells [102]. That observation is best understood in the context of the proteostatic mechanism developed below. The second is that phosphoinositide 3-kinase α was independently recovered as a candidate target in degradation-based profiling [89]. That method reports degradation rather than binding, so the assignment remains an inference, but it offers a proximal explanation for effects previously interpreted as indirect. In hormone-responsive disease, the pathway is engaged through leptin: celastrol inhibits proliferation and migration of MCF-7 cells through the leptin-triggered PI3K/Akt axis [103]. This finding acquires additional significance in the light of the adipocyte and tumour crosstalk documented at the obesity and cancer interface [104]. In the same cell line, celastrol suppresses viability through activation of AMP-activated protein kinase [105], and AMPK reappears below as the intermediary linking heat shock factor 1 to the Hippo effector YAP [106].

#### 4.3.3. JAK and STAT Signalling

STAT3 is the principal node linking celastrol to cancer stemness in gynaecological tumours, and semisynthetic derivatives have been rationally optimised against it for anti-ovarian cancer stem cell activity [64,65]. In breast cancer, the connection has now been given a direct molecular basis: inhibition of the mitochondrial isocitrate dehydrogenases leads through DPYSL2 sequestration to inactivation of JAK and STAT signalling [87]. STAT3 additionally controls the endothelial compartment. Celastrol suppresses the mitochondrial fusion protein OPA1 at the messenger RNA level by inhibiting STAT3 phosphorylation, and the STAT3 inhibitor Stattic reproduces both the suppression of OPA1 and the antiangiogenic effect [107]. Network pharmacology combined with transcriptomic data, machine learning, molecular dynamics simulation, and single-cell RNA sequencing has independently prioritised CTNNB1 and STAT3 as core celastrol targets, although in a tumour type outside the present scope [108]. One observation runs in the opposite direction and should not be smoothed over. In melanoma, celastrol upregulated major histocompatibility complex class I through activation of the JAK and STAT1 pathway [92]. The compound therefore suppresses STAT3-dependent survival signalling while enhancing STAT1-dependent antigen presentation. The two effects are not contradictory, because the STAT proteins serve opposing functions in tumour immunity, but they illustrate that a description of celastrol as a generic inhibitor of JAK and STAT signalling would be inaccurate.

#### 4.3.4. MAPK, ERK, JNK, and p38

The mitogen-activated protein kinase cascades are engaged in a manner that depends on the branch considered. Reactive oxygen species-mediated activation of JNK, together with suppression of PI3K and Akt signalling, has been identified as a principal route to celastrol-induced apoptosis, although the demonstration was made in multiple myeloma rather than in the tumour types considered here [101]. In triple-negative breast cancer, attenuation of amphiregulin expression proceeds through inhibition of MAPK signalling [109]. The MEK and p38 branches are required for the paraptotic phenotype described below, since inhibitors of these pathways prevent the formation of cytoplasmic vacuoles in HeLa cells [110]. MAPK1 has also been nominated as the node through which celastrol simultaneously induces immunogenic cell death and downregulates programmed death ligand 1 [111]. MAPK signalling has been proposed, on the basis of bioinformatic analysis rather than direct pathway manipulation, to mediate the reprogramming of macrophage polarity produced by celastrol acting as a histone deacetylase inhibitor [112].

#### 4.3.5. Wnt, Hippo, and Notch Signalling

This group of pathways is the least developed part of the current literature on celastrol in the cancers considered here, yet it contains some of the most direct evidence available. A NanoLuc biosensor designed to quantify the interaction between YAP or TAZ and TEAD was used in a high-throughput screen, which identified celastrol as an inhibitor of this protein–protein interaction. The activity was examined further in MDA-MB-231 cells, where the compound inhibited proliferation, viability, and migration; colony formation was assessed in H1299 lung cancer cells [113]. No clinically available drug targets the YAP and TEAD interface, and celastrol therefore represents one of a small group of chemical starting points for Hippo pathway inhibition. The Hippo and Wnt pathways are connected by celastrol through a defined cascade. The compound increases transcriptional activation of liver kinase B1 partly through heat shock factor 1, LKB1 activates AMP-activated protein kinase α, AMPKα phosphorylates YAP, and phosphorylated YAP promotes degradation of β-catenin. Deficiency of LKB1 attenuated the inhibitory effect of celastrol both in vitro and in vivo [106]. The reciprocal experiment is equally informative. Inducible overexpression of TAZ partially rescued cells from celastrol-induced apoptosis, increased Bcl-2, decreased Bax, induced the target genes ANKRD, CYR61, and CTGF, and activated PI3K and Akt signalling, although this was demonstrated in T-Rex-293 and HEK293 embryonic kidney cells rather than in a tumour line [114]. Taken together, these studies suggest that the activity of the Hippo effectors is a determinant of sensitivity to celastrol and therefore a candidate predictive biomarker.

Direct evidence in a mammary model comes from an in vivo study in mice bearing Ehrlich solid tumours. Celastrol at 2 mg/kg given intraperitoneally for 21 days downregulated β-catenin and its downstream targets cyclin D1 and survivin, increased p53 and caspase-3, raised glutathione content, lowered malondialdehyde, interleukin 6, and interleukin 1β, and inhibited vascular endothelial growth factor (VEGF)-mediated angiogenesis. The authors propose on this basis that combination with doxorubicin merits investigation [115].

The Notch pathway remains the weakest link. In triple-negative breast cancer, celastrol and triptolide reduced Notch-1 activation along with the canonical target genes HES-1 and HEY-1, decreased Jagged-1 and the Notch intracellular domain NICD1, and suppressed mammosphere formation and the stem cell markers DCLK1, ALDH1, and CD133. Ectopic expression of NICD1 partially rescued proliferation and mammosphere formation, which establishes the effect as canonical rather than correlative [116]. The leukaemia study cited above documented downregulation of Notch1 with cytoplasmic retention of p65 [100]. The most informative recent work concerns a gynaecological tumour, although not one of the three considered here. In uterine corpus endometrial carcinoma, celastrol inhibited viability in a dose-dependent manner, downregulated CDK2 and cyclin E, upregulated p21, and produced DNA damage and apoptosis evidenced by TUNEL positivity, caspase-3 activity, and expression of cleaved caspase-9, cleaved poly(ADP-ribose) polymerase 1 (PARP1), and γ-H_2_AX. Tumour growth was repressed in xenograft-bearing nude mice [117]. Mechanistically, the lysine acetyltransferase KAT2B was identified as the mediator: it was upregulated by celastrol, its overexpression reproduced the phenotype, and its knockdown abolished the effect. Coimmunoprecipitation showed that KAT2B binds the Notch effector RBPJ and increases its acetylation, and RBPJ in turn binds the MCM4 promoter and represses its transcription [117]. A recent study in nonalcoholic steatohepatitis provides the only contemporary readout at the level of the Notch intracellular domain, reporting suppression of Notch and osteopontin signalling, although in a nonneoplastic setting [118]. Canonical Notch signalling has therefore been examined in triple-negative breast cancer only; no primary study has addressed it in ovarian or cervical cancer.

#### 4.3.6. Hormone Receptor and Growth Factor Signalling

This block is specific to breast cancer and is the best populated part of the existing literature. Celastrol shows anticancer activity in combination with ErbB2-targeted therapeutics in ErbB2-overexpressing disease [119], and it downregulates HER2 and alters its subcellular distribution in MDA-MB-453 cells [120]. This effect is readily interpreted as the loss of a client protein following disruption of the Hsp90 and Cdc37 complex. In oestrogen receptor-positive disease, celastrol inhibits growth through modulation of receptor signalling [121], and a protein-fragment complementation assay demonstrated that celastrol and gambogic acid suppress mutant forms of the oestrogen receptor α [122]. Such mutations underlie acquired resistance to endocrine therapy, which makes this observation of practical interest. Consistent with this, celastrol enhances tamoxifen sensitivity in triple-negative breast cancer through a mitochondrial mechanism [123] and acts synergistically with tamoxifen on apoptosis and autophagy in MCF-7 cells [124]. The SGK3, STAT3, and ZMIZ2 axis has been described in oestrogen receptor-positive proliferation [125], and epidermal growth factor receptor signalling is implicated in the restoration of anoikis sensitivity in triple-negative disease [126].

#### 4.3.7. Hypoxia, Angiogenesis, and the Invasive Phenotype

The reference study for this activity showed that celastrol suppresses angiogenesis-mediated tumour growth. At 2 mg/kg per day, the compound reduced the volume and weight of PC-3 xenografts and decreased tumour vascularity. It also inhibited VEGF-induced proliferation, migration, invasion, and capillary-like structure formation by human umbilical vein endothelial cells, abrogated VEGF-induced sprouting from aortic rings, and blocked vascular formation in the Matrigel plug assay, with suppression of VEGFR2 activation and of downstream AKT, mTOR, and p70S6K signalling [127]. Two subsequent studies added mechanisms that lie outside the canonical VEGF axis. Celastrol suppressed glioma vasculogenic mimicry and angiogenesis through blockade of PI3K, Akt, and mTOR signalling, lowering VEGFA, VEGFR2, angiopoietin 2, and HIF-1α as well as the vasculogenic mimicry proteins EphA2 and VE-cadherin. Periodic acid–Schiff and CD31 double staining confirmed suppression of both true vessels and mimicry channels [128]. Independently, the antiangiogenic effect was traced to mitochondrial dynamics, because celastrol suppressed OPA1-dependent mitochondrial fusion in endothelial cells, impaired mitochondrial energy metabolism in the Seahorse assay, and triggered mitochondrial fragmentation, acting through the STAT3, OPA1, and p65 axis [107].

The behaviour of HIF-1α illustrates a genuine inconsistency in the literature that a review of this kind should state rather than resolve prematurely. In hepatoma cells under hypoxia, celastrol markedly decreased the accumulation of HIF-1α protein in a dose-dependent manner without affecting HIF-1β or topoisomerase I, and prevented induction of the target genes encoding VEGF and erythropoietin. The effect was exerted by inhibiting HIF-1α protein synthesis through dephosphorylation of mTOR, p70S6K, and eIF4E and of ERK1/2, without any change in HIF-1α messenger RNA or in protein degradation [129]. In the same year, and in multiple cancer cell lines, celastrol was reported to induce HIF-1α protein accumulation in an oxygen-independent manner, to promote its nuclear entry, and to increase transcription of VEGF and GLUT1. These effects were attributed to the generation of reactive oxygen species and to activation of Akt and p70S6K signalling, which enhanced HIF-1α translation [130]. The same study showed that Akt activation was transient and was followed, with longer exposure, by depletion of Akt protein consequent on inhibition of Hsp90 chaperone function. The authors noted explicitly that the concentration required to induce HIF-1α accumulation lay below the cytotoxic threshold [130]. The two reports are therefore best reconciled as a description of the same system sampled at different doses and exposure times, with a low-dose, early phase dominated by reactive oxygen species-driven Akt activation and a higher-dose, later phase dominated by loss of Hsp90 client proteins. This interpretation has direct practical consequences for the design of sustained-release nanoformulations, since the exposure profile achieved by a carrier will determine which of the two regimes predominates.

In breast cancer specifically, celastrol inhibits angiogenesis and the migratory and invasive behaviour of MDA-MB-231 cells by downregulating degenerative spermatocyte homolog 1 and thereby reducing production of sphingosine 1-phosphate. Knockdown and overexpression of DEGS1 reproduced and reversed these effects. Coculture experiments showed that endothelial cells promote proliferation, migration, and invasion of MDA-MB-231 cells through sphingosine 1-phosphate and its receptors, a process that celastrol interrupted [131]. Broader lipidomic analysis, performed in 3T3-L1 adipocytes and in wild-type and Fxr-null mice rather than in a tumour model, has shown that celastrol modifies the metabolism of lysophosphatidylcholines, phosphatidylcholines, sphingomyelins, and phosphatidylethanolamines, providing a wider metabolic context for the sphingolipid effect [132]. The invasive phenotype is additionally restrained through suppression of matrix metalloproteinase 9 [22,133] and of integrin signalling in triple-negative disease [134], and celastrol enhances the antitumour effect of docetaxel while inhibiting migration and invasion [135].

### 4.4. Collapse of Proteostasis as a Unifying Mechanism

The individual observations collected above are frequently presented as evidence that celastrol acts on an unusually large number of unrelated pathways. A more economical reading is available, and it accounts for a substantial part of the phenotype. Celastrol inhibits the catalytic activity of the proteasome [75,76] and destabilises the Hsp90 and Cdc37 complex that loads protein kinases onto the chaperone [78,79,80]. These two activities converge on a single cellular outcome, namely the loss of the capacity to fold, triage, and degrade client proteins. The activation of heat shock factor 1 that accompanies them [81] belongs to the same event but acts in the opposite direction: the heat shock response is induced because proteostasis is failing, and by restoring chaperone capacity it limits the damage, which is one reason why cell death is only partially dependent on this axis.

The consequences observed in cancer cells follow directly from this. Celastrol promotes proteotoxic stress in glioblastoma cells, evidenced by accumulation of polyubiquitinated aggregates and of the autophagy substrate p62 and by induction of Hsp72 and Hsp90, with cell death only partially dependent on PARP1 and caspase 3 and therefore not reducible to classical apoptosis. The same study demonstrated synergy with a pharmacological Hsp90 inhibitor [70]. In hepatocellular carcinoma, celastrol activated the PERK and IRE1α arms of the unfolded protein response and caused G2/M arrest and intrinsic apoptosis, with tumour growth inhibited in a syngeneic model [136]. The observation that tumour necrosis factor α sensitises breast cancer cells to natural products with proteasome-inhibitory activity points in the same direction [137].

**Figure 3:**
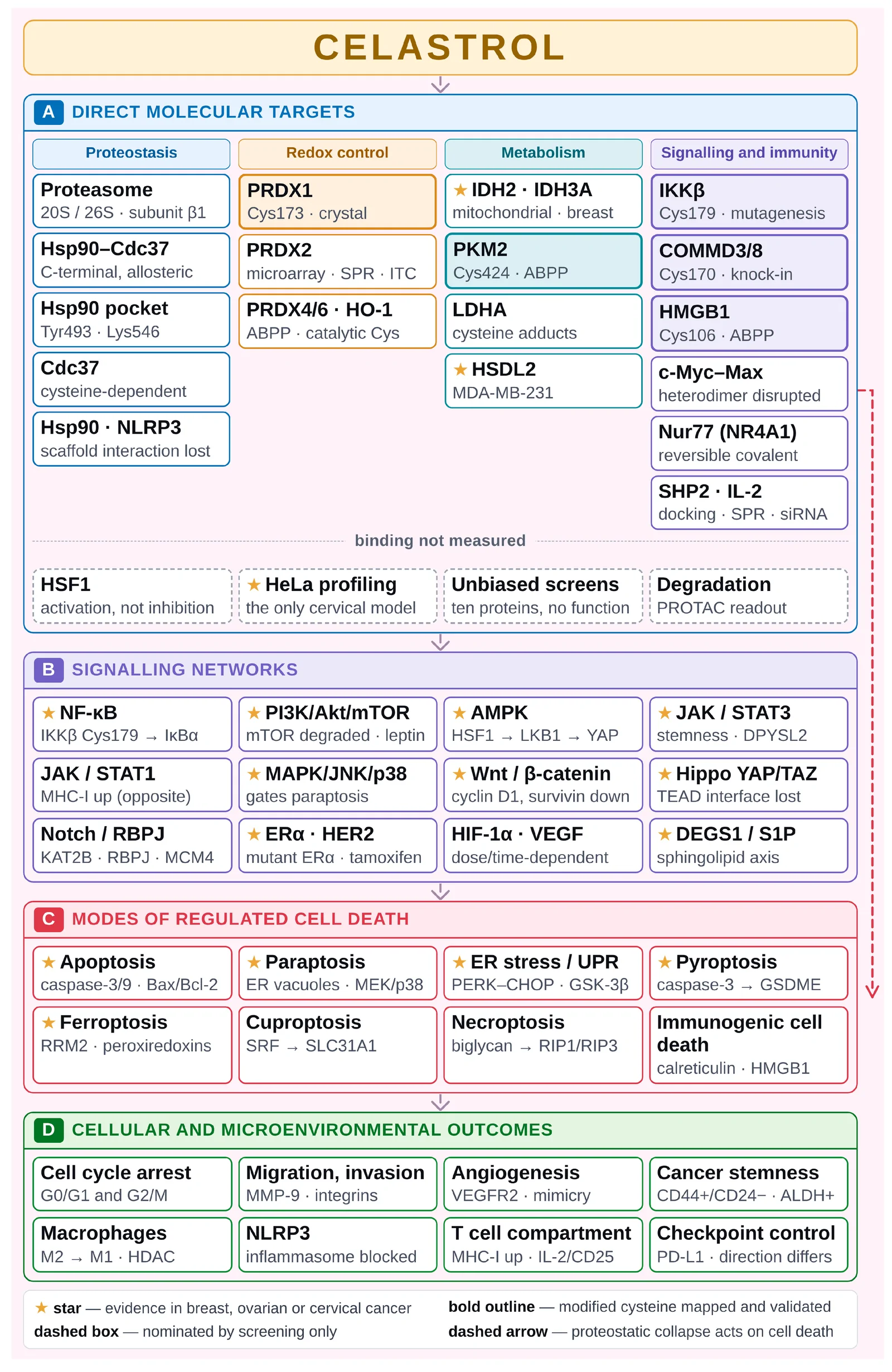
Molecular targets and signalling networks of celastrol. The direct molecular targets (**A**) are grouped by cellular function and feed into the signalling networks (**B**), the modes of regulated cell death (**C**), and the cellular and microenvironmental outcomes (**D**). Entries below the dashed rule in (**A**) were nominated by a functional readout, by unbiased screening, or by degradation-based profiling, and physical binding was not measured for them. A star marks relationships for which evidence exists in breast, ovarian, or cervical cancer; a bold outline marks the five targets for which the modified cysteine has been mapped and functionally validated. The dashed arrow indicates the route by which collapse of protein quality control acts on cell death independently of the intermediate signalling layer. Arrows in (**B**–**D**) indicate reported relationships and are not claims of causal necessity. The figure was developed using Python 3.14.

The value of this framework is that it explains observations that would otherwise require separate mechanisms. The ubiquitin-dependent degradation of mTOR in breast cancer cells [102], the downregulation and mislocalisation of HER2 in MDA-MB-453 cells [120], the loss of Akt protein following prolonged exposure [130], and the reduction in cyclin D1 and survivin in mammary tumours [115] are all consistent with the destabilisation of a set of proteins that share dependence on the Hsp90 and Cdc37 system rather than membership of a common signalling pathway. Under this interpretation the apparent promiscuity of celastrol at the level of pathways is in part a downstream reflection of a focused action at the level of protein quality control. The organisation of direct targets, signalling networks, death modalities, and phenotypic outcomes, with an indication of which relationships have been established in breast, ovarian, or cervical cancer, is summarised in Figure 3.

### 4.5. Modes of Regulated Cell Death

Apoptosis is the mode of death most frequently reported for celastrol in breast, ovarian, and cervical cancer. Its dependence on p53 is partial rather than absolute, as shown in a study using osteosarcoma lines of defined p53 status. Combination of celastrol with 5-fluorouracil reduced half-maximal inhibitory concentrations by 3.7- to 11.9-fold and enhanced apoptosis, through p53-dependent mechanisms in wild-type cells and p53-independent mechanisms in p53-null and p53-mutant cells. In each case Bax and caspase 9 were upregulated and Bcl-2 downregulated, with additional inhibition of P-glycoprotein efflux [138]. The mechanistic literature of the past decade has established that apoptosis is neither the only nor, under some conditions, the dominant outcome, and the alternative modalities are directly relevant to the nanoformulations considered later in this review and to the combination strategies that exploit them.

Paraptosis was the first non-apoptotic modality to be characterised. Celastrol induced paraptosis-like cytoplasmic vacuolisation in HeLa, A549, and PC-3 cells. In HeLa cells, the vacuoles derived from dilation of the endoplasmic reticulum, and markers of autophagy were present, including conversion of LC3-I to LC3-II and formation of LC3 puncta. Inhibitors of autophagy did not interrupt vacuolisation but enhanced it, whereas inhibitors of the MEK and p38 pathways prevented it. G2/M arrest and apoptosis occurred in parallel [110]. Endoplasmic reticulum stress was subsequently shown to be the proximate trigger of apoptosis in the same cell line. In that work the catalytic β1 subunit of the proteasome was confirmed as a direct target, endoplasmic reticulum protein 29 and the mitochondrial import receptor TOM22 were identified by two-dimensional electrophoresis, and glycogen synthase kinase 3β acted as the central node of the network linking endoplasmic reticulum stress to mitochondrial apoptosis [76].

Pyroptosis has been added more recently and, importantly for the present review, also in cervical cancer cells. Celastrol induced cell swelling with large bubbles consistent with a pyroptotic phenotype and cleaved gasdermin E; the morphology was abolished by CRISPR/Cas9-mediated knockout of GSDME in HeLa cells. Cleavage of GSDME was attenuated by a specific caspase 3 inhibitor, and endoplasmic reticulum stress and autophagy were shown to lie upstream [139]. Necroptosis has been documented in gastric cancer, where celastrol downregulated biglycan, activated receptor-interacting protein kinases 1 and 3, and promoted translocation of mixed-lineage kinase domain-like protein to the plasma membrane, with the effect reversed by overexpression of biglycan and accompanied by suppression of tumour necrosis factor α and interleukin 8 release [140].

Ferroptosis is now supported by several independent lines of evidence. In hepatocellular carcinoma, celastrol raised reactive oxygen species and malondialdehyde and depleted glutathione. Bioinformatic analysis of TCGA and FerrDb data combined with molecular docking identified ribonucleotide reductase M2 as the mediator; its inhibition reduced mTOR phosphorylation and its overexpression opposed the effect [141]. In non-small cell lung cancer, the ferroptosis inducer erastin synergised with celastrol at otherwise nontoxic concentrations through activation of a reactive oxygen species, mitochondrial fission, and mitophagy axis, although that study did not establish that celastrol alone is ferroptotic in this model [142]. Activity-based protein profiling linked adduct formation on the catalytic cysteines of peroxiredoxins and on haem oxygenase 1 to a ferroptotic outcome, although in hepatic stellate cells rather than in a tumour [84]. The most directly relevant evidence is that ferroptosis was one of the two terminal events of the mitochondrial isocitrate dehydrogenase mechanism defined in breast cancer [87].

Cuproptosis is the most recently described modality and is of particular relevance because carrier-free celastrol and copper nanomedicines are predicated on it without any account of the underlying biology. In non-small cell lung cancer, the copper chelator tetrathiomolybdate rescued cells from celastrol-induced death more effectively than other inhibitors. RNA sequencing showed marked upregulation of the copper importer SLC31A1 downstream of serum response factor, and the cells displayed the canonical features of cuproptosis, namely intracellular copper accumulation, glutathione depletion, loss of the iron-sulfur cluster proteins FDX1, SDHB, and POLD1, induction of Hsp70, and oligomerisation of dihydrolipoamide S-acetyltransferase [143].

Immunogenic cell death occupies a special position because it is invoked repeatedly in the nanomedicine literature yet rests on a narrow experimental base for the free compound. In clear cell renal cell carcinoma, bioinformatic analysis combined with experimental validation identified EGFR, IKBKB, PRKCQ, and MAPK1 as the targets underlying celastrol-induced immunogenic cell death. The same work showed that the compound triggered autophagy, increased high mobility group box 1 and calreticulin, and downregulated programmed death ligand 1 through inhibition of MAPK1 [111]. A subsequent study in a hepatocellular carcinoma model with malignant ascites compared free celastrol with a hydrogel formulation; activation of dendritic cells and infiltration of cytotoxic T cells were attributed to the formulated compound. The increase in tumour PD-L1 expression, which provided the rationale for combination with an anti-PD-1 antibody, was attributed to celastrol itself [144]. The direction of the effect on PD-L1 therefore differs between the two studies, and because one of them used a formulated compound, the discrepancy cannot presently be resolved.

Finally, calcium handling links several of these modalities. Release of calcium from the endoplasmic reticulum followed by influx into mitochondria has been identified as a trigger of celastrol-induced death [145], and mitochondrial calcium overload has been placed downstream of the mTORC2 and Akt axis [146]. Since the endoplasmic reticulum is also the site of the vacuolisation seen in paraptosis and the origin of the unfolded protein response, calcium dysregulation may represent the common intermediate between proteostatic collapse and the choice of death modality.

### 4.6. The Tumour Immune Microenvironment

Celastrol modifies the tumour immune microenvironment through at least three distinguishable mechanisms, and the resulting picture is not uniformly favourable to antitumour immunity.

The first concerns macrophages. Celastrol inhibits metastasis by suppressing M2-like polarisation [147], and the underlying mechanism has since been attributed to an unexpected pharmacological property: celastrol acts as a histone deacetylase inhibitor and reverses macrophage polarity from M2 to M1. This effect was demonstrated in macrophages exposed to conditioned medium from colorectal cancer cells and confirmed in APCmin/+ mice, with the involvement of MAPK signalling inferred from bioinformatic analysis [112]. This is the only report placing celastrol among epigenetic modulators, and it warrants independent confirmation.

The second concerns the NLRP3 inflammasome. Celastrol abolishes NLRP3 inflammasome activation, inhibits caspase 1 activation and interleukin 1β secretion in vitro and in vivo, and does so through interruption of ASC oligomerisation and activation of autophagy [148]. The effect is selective for NLRP3 and does not extend to the AIM2 or NLRC4 inflammasomes [149], which distinguishes it from generic anti-inflammatory activity. A recent molecular explanation connects this activity to the chaperone mechanism: celastrol binds Hsp90 directly and blocks its interaction with NLRP3, as shown by molecular docking, biolayer interferometry, and coimmunoprecipitation [96]. In a tumour context, inhibition of the NLRP3 inflammasome in macrophages of the microenvironment suppressed the metastatic potential of melanoma cells [150]. In diethylnitrosamine-induced hepatocarcinogenesis, the combination of celastrol at 2 mg/kg with metformin at 200 mg/kg improved liver function and survival while downregulating NLRP3, suppressing caspase 1 activity, and reducing the mature forms of interleukin 1β and interleukin 18. Pyroptotic activity was thereby reduced and apoptotic potential enhanced [151].

The third concerns lymphocytes, and here two studies define direct protein targets. A screen for inhibitors of the interleukin 2 and CD25 interaction identified celastrol, which binds interleukin 2 itself rather than CD25, suppresses the proliferation and signalling of interleukin 2-dependent murine T cells, and interferes with interleukin 2-responsive STAT5 phosphorylation. In C57BL/6 mice bearing B16F10 tumours, the compound inhibited tumour growth and increased CD8 positive T cells, and it produced no effect in T cell-deficient nude mice. The antitumour activity in this model is therefore immune-dependent [95]. More recently, celastrol was shown in vitro to upregulate major histocompatibility complex class I on B16F10 melanoma cells through activation of JAK and STAT1 signalling, with SHP2 nominated as the target by docking, surface plasmon resonance, and small interfering RNA. Pretreated tumour cells enhanced CD8 positive T cell activation and the secretion of interferon γ and tumour necrosis factor α. In vivo, the compound inhibited tumour growth at 1 and 2 mg/kg but not at 0.5 mg/kg, and it synergised with immune checkpoint inhibitors [92].

These findings must be read alongside evidence that celastrol is also immunosuppressive. Dissociation of the COMMD3 and COMMD8 complex inhibits B cell migration and reduces antibody responses, an effect reproduced by genetic deficiency of the complex and abolished in mice carrying a celastrol-resistant mutant [91]. Suppression of interleukin 1β maturation, while advantageous where the cytokine drives invasion, removes a signal that contributes to dendritic cell-dependent antitumour immunity in other settings. The net immunological effect of celastrol in a given tumour is therefore likely to depend on which of these arms predominates, and this is not currently predictable from the available data.

### 4.7. Critical Appraisal and Gaps

Several limitations of the evidence reviewed above should be stated explicitly.

The first is the imbalance between the breadth of the target list and the depth of validation behind individual entries. Residue-level mapping of the covalent adduct is available for five proteins only, and genetic validation using a drug-resistant mutant has been performed for one. These five proteins constitute Category 1 of Table 2. Of the remaining entries, the larger part falls into Category 2, in which binding or direct enzymatic inhibition has been measured but the modified residue has not been mapped, and a substantial minority into Category 3, in which no measurement of binding to the individual protein exists at all. A considerable number of the remaining assignments derive from a single laboratory using a single method, and orthogonal confirmation is frequently absent. In several cases the assignment rests on inference rather than on a binding measurement, as in degradation-based profiling [89], and in others the affinity reported is weak relative to the concentrations used in cellular experiments [92]. Reviews that enumerate celastrol targets without weighting them by evidence quality risk conveying a false impression of mechanistic certainty [72,74].

The second is the mismatch of biological context. Almost all target identification has been performed in colorectal, gastric, hepatic, pulmonary, prostatic, or haematological models, or in nonneoplastic systems. Breast cancer is represented by a single chemoproteomic study [87] and by the identification of HSDL2 [93], cervical cancer by one competitive profiling experiment [94], and ovarian cancer by none. The mechanistic account presented in this section is therefore a synthesis across tumour types, and the assumption that the same targets dominate in the cancers considered here has not been tested.

The third is the mismatch of concentration. Most mechanistic experiments employ celastrol at concentrations between 1 and 10 μmol/L, whereas systemic exposure achievable in vivo is constrained by the toxicity and poor oral bioavailability of the compound [16,26,27,29]. The divergent findings on HIF-1α [129,130] demonstrate that the direction of a celastrol effect can invert within this range. Extrapolation of high-concentration mechanisms to plausible therapeutic exposures is therefore a substantive rather than a formal concern, and it provides one of the strongest arguments for the delivery strategies discussed later in this review.

The fourth concerns the pathways for which no data exist. No primary study has examined the effect of celastrol on Hedgehog, Smoothened, or GLI signalling in any tumour model. No study has addressed myeloid-derived suppressor cells or the cyclic GMP-AMP synthase and stimulator of interferon genes axis for the free compound. Canonical Notch signalling has been examined only in triple-negative breast cancer, and not in ovarian or cervical cancer. These are not deficiencies of the present review but genuine gaps in the field, and they define a tractable experimental agenda.

## 5. In Vitro and In Vivo Application of Celastrol Against Breast, Ovarian, and Cervical Cancer

The anticancer potential of celastrol has been extensively investigated in both in vitro and in vivo models, which have demonstrated broad antitumour activity through multiple mechanisms, including induction of apoptosis, cell-cycle arrest, inhibition of metastasis, suppression of oncogenic signalling pathways, and targeted protein degradation.

Kannaiyan et al. (2011) [101] demonstrated in vitro that celastrol was broadly cytotoxic across a large panel of human cancer lines (myeloma including drug-resistant clones, liver, gastric, prostate, renal, head and neck, lung, melanoma, glioma, and the breast lines MCF-7 and MDA-MB-231), with IC_50_ values of approximately 0.52 to 4.7 μM and relative sparing of normal cells. The authors found that celastrol downregulated cyclin D1 and cyclin E, raised p21 and p27, suppressed XIAP, cFLIP, Bcl-2, Bcl-xL, survivin, c-Myc, and VEGF, and induced apoptosis mediated by caspase-8, caspase-9, and caspase-3. The effect required JNK activation and suppression of PI3K/Akt signalling, as the JNK inhibitor SP600125 abolished apoptosis [101].

Raja et al. (2011) [119] investigated celastrol specifically in ErbB2-overexpressing breast cancer using both in vitro and in vivo models. Celastrol proved selectively potent towards ErbB2-high lines (SK-BR-3, BT-474, 21MT-1, and trastuzumab-resistant JIMT-1; SK-BR-3 IC_50_ 0.13 μM) versus ErbB2-low MCF-7 and normal MCF-10A and showed strong synergy with trastuzumab and lapatinib. In a BT-474 xenograft in NOD-SCID mice, celastrol at 2 and 4 mg/kg retarded tumour growth, with 2 mg/kg providing efficacy at an acceptable level of toxicity. Mechanistically, it triggered ubiquitin- and lysosome-mediated degradation of ErbB2 and other Hsp90 client proteins, G1 arrest, apoptosis, and ROS generation, all of which depended on its Michael acceptor group [119].

Further evidence for the pro-apoptotic activity of celastrol was provided by Mezzanotte et al. (2014) [152], who combined in vitro and in vivo work using a triple-luciferase MDA-MB-231 breast cancer platform that simultaneously reports cell viability, NF-κB activity, and cell sorting. Caspase-3/7 activity was read out separately using the Z-DEVD-aminoluciferin substrate. Celastrol was one of five natural compounds screened (at 0.1 to 2.5 μM, alongside sulforaphane, curcumin, resveratrol, and betulinic acid); it dose-dependently inhibited TNFα-induced NF-κB activity, reduced nuclear p65, and induced caspase-dependent apoptosis at 1 μM. In a mammary fat pad model, intratumoural celastrol at 2 mg/kg inhibited NF-κB signalling and induced caspase activity in vivo [152].

In addition to the direct effects on tumour cells described above, Yang et al. (2018) [147] showed that celastrol suppressed breast cancer metastasis by blocking M2-like polarisation of macrophages rather than by direct tumour cytotoxicity. Using RAW 264.7 cells, bone marrow-derived macrophages and 4T1 cells, celastrol (non-toxic at or below 125 nM, 62.5 nM in assays) reduced IL-13-induced expression of CD206 and M2 marker genes. In an orthotopic 4T1 model in BALB/c mice, celastrol at 10 mg/kg daily for 14 days lowered the number of lung metastases without changing primary tumour weight. The effect was attributed to abrogation of STAT6 phosphorylation [147].

Using a comparative spontaneous tumour model, Ou et al. (2021) [153] further confirmed the anticancer activity of celastrol in canine mammary tumours, which closely resemble human triple-negative breast cancer. In CMT-7364 and CIPp cell lines, celastrol (0.1 to 1.6 μM) reduced cell viability, producing 24 h IC_50_ values of approximately 1.2 μM while exhibiting lower toxicity towards normal MDCK cells. The treatment inhibited colony formation, migration, and invasion, induced caspase-dependent apoptosis through increased caspase-3, caspase-9, and Bax expression together with decreased Bcl-2, NF-κB, and phospho-p65 levels. These apoptotic effects were reversed by the caspase inhibitor Z-VAD-FMK. Celastrol also induced G2/M cell-cycle arrest through increased p21 and p27 expression and decreased cyclin D1 levels [153].

More recently, efforts have focused on improving the pharmacological properties of celastrol through targeted protein degradation strategies. Gan et al. (2024) [59] developed celastrol-based PROTAC molecules (compounds **6a**–**6d**), identifying the short-linker compound **6a** as the most effective degrader against triple-negative breast cancer. In 4T1 and other breast cancer cell lines, compound **6a** displayed greater potency than celastrol, inhibiting colony formation and migration at concentrations of 100 to 500 nM and increasing apoptosis to approximately 52.6% at 1 μM. Mechanistically, compound **6a** simultaneously degraded GRP94, CDK1, and CDK4 through the ubiquitin-proteasome system, resulting in cell-cycle arrest and activation of the Bcl-2/Bax/caspase-3 apoptotic pathway. In 4T1-bearing BALB/c mice, treatment with 3 and 6 mg/kg produced greater tumour suppression than celastrol while remaining well tolerated [59].

Similarly, Gu et al. (2025) [58] reported the development of YX-112, a celastrol-derived PROTAC incorporating a rigid linker to improve solubility. In vitro and computational analyses indicated that YX-112 was particularly potent against MDA-MB-231 cells, exhibiting an IC_50_ of 0.32 μM, approximately three-fold lower than that of celastrol, together with a selectivity index of 3.50 relative to normal HK-2 cells. Quantitative proteomic analysis identified alterations in cell-cycle regulation and mTOR signalling, while mechanistic experiments showed proteasome-dependent degradation of CHEK1 and PIK3R2, which was blocked by MG132. In addition, YX-112 downregulated CDK4 and phosphorylated AKT more effectively than celastrol, supporting a mechanism involving dual protein degradation and modulation of PI3K/Akt/mTOR signalling [58].

Xu et al. (2019) [20] examined celastrol in ovarian cancer in vitro and in vivo. In A2780 and SKOV3 cells, celastrol produced IC_50_ values of 2.11 and 2.29 μM, respectively, while inducing G2/M arrest and apoptosis accompanied by intracellular ROS accumulation. Importantly, the ROS scavenger N-acetyl-cysteine abolished apoptosis, indicating that ROS generation was required for the antitumour response. Associated signalling changes involved the p38, JNK, ERK, AKT, and RAF1 pathways. In an A2780 xenograft model established in BALB/c nude mice, daily intraperitoneal administration of celastrol at 2 mg/kg inhibited tumour growth by 28.6% without apparent toxicity, leading the authors to conclude that celastrol represents a potential therapeutic candidate for ovarian cancer [20].

In contrast to these anticancer studies, Jiang et al. (2025) [154] investigated celastrol in the context of ovarian ageing rather than cancer. Using C57BL/6J mice together with porcine granulosa cells and oocytes, the authors demonstrated that celastrol administered at 3 mg/kg intraperitoneally every other day for three weeks promoted follicular development in young mice and reduced granulosa cell apoptosis in 12-month-old animals, whereas 6 mg/kg produced toxic effects. Oocyte maturation was assessed separately in porcine oocytes in vitro at 0.5 μM. Mechanistically, insulin receptor substrate 1 (IRS1) was identified as the critical mediator, as IRS1 overexpression enhanced follicular development and maturation while its knockdown inhibited these processes. Although this was not an oncology study, the findings highlight the dose-dependent biological activity of celastrol and illustrate that its molecular targets may produce beneficial effects in non-cancerous tissues under appropriate conditions [154].

In an in vitro metabolomics study, the effects of celastrol on human cervical cancer (HeLa) cells were investigated using gas chromatography–mass spectrometry (GC-MS)-based metabolic profiling. Celastrol inhibited HeLa cell proliferation in a dose- and time-dependent manner (2.5 to 10 μM), induced concentration-dependent apoptosis, caused mitochondrial membrane depolarisation, and significantly increased caspase-3 activity, confirming activation of the intrinsic apoptotic pathway. Metabolomic analysis identified 14 significantly altered metabolites associated with energy, amino acid, and nucleotide metabolism. Celastrol reduced tricarboxylic acid (TCA) cycle intermediates (isocitric acid, α-ketoglutarate, and malic acid), decreased serine, glycine, and cysteine levels, and increased fructose, lactate, inosine, guanosine, and 1,4-butanediamine, indicating impaired mitochondrial respiration, enhanced glycolysis (Warburg effect), disrupted amino acid and protein biosynthesis, and altered nucleotide metabolism. Comparison with cisplatin, doxorubicin, and paclitaxel showed that celastrol produced similar apoptosis-associated metabolic alterations while uniquely decreasing intracellular cysteine, suggesting covalent interaction with cysteine thiol groups through its quinone methide moiety. The authors concluded that GC-MS-based metabolomics provides a global view of celastrol-induced metabolic reprogramming and supports mitochondrial dysfunction, apoptosis, and disruption of cellular metabolism as key mechanisms underlying its anticancer activity in cervical cancer cells [21].

**Figure 4:**
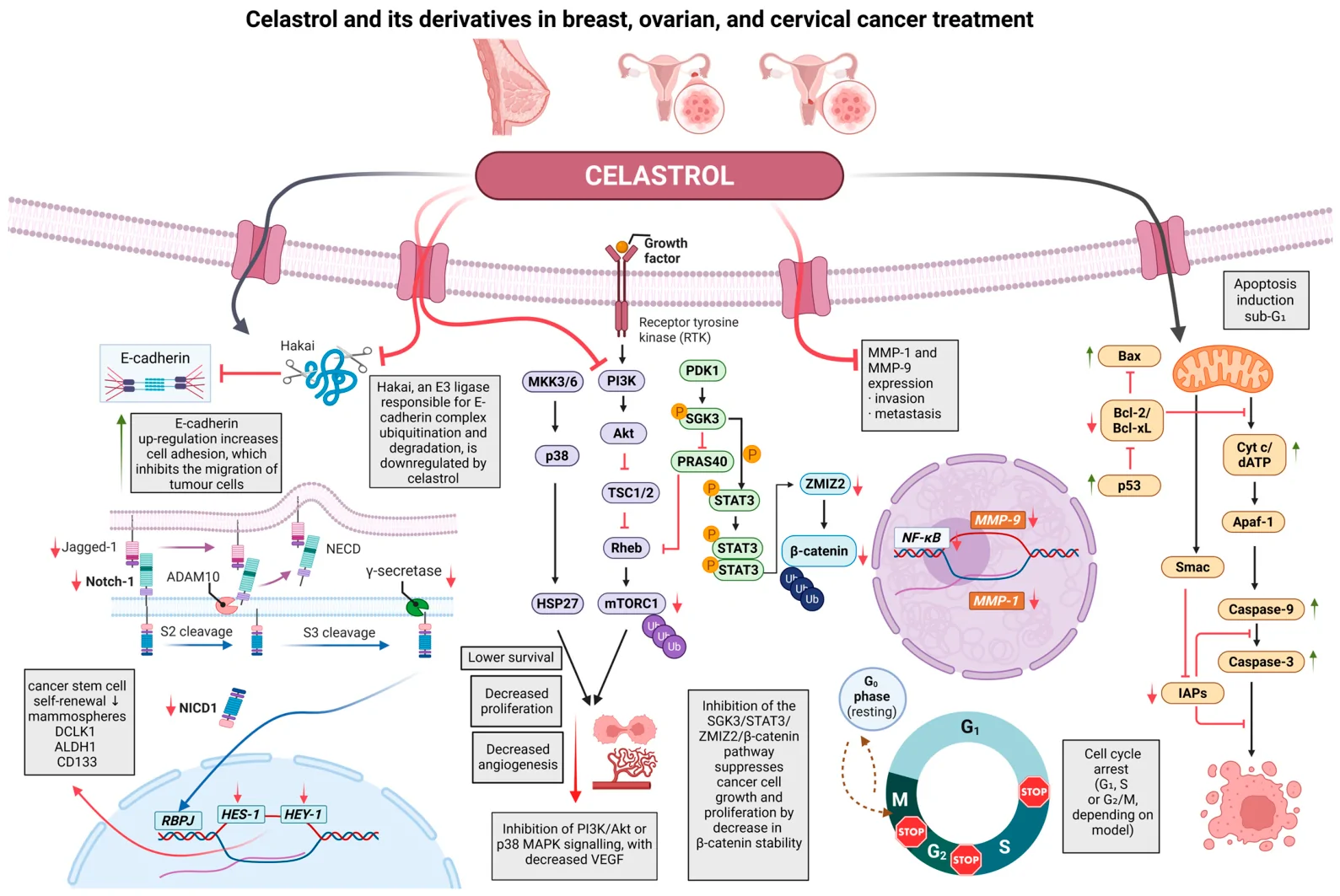
Biological effects of celastrol in breast, ovarian, and cervical cancer reported across the studies compiled in Table 3. Left: suppression of Hakai-mediated E-cadherin degradation and of Notch signalling, restoring cell adhesion and reducing cancer stem cell self-renewal. Centre: blockade of receptor tyrosine kinase signalling at phosphoinositide 3-kinase, with inhibition of the Akt–mTORC1 and p38 branches and of the SGK3/STAT3/ZMIZ2 axis, lowering β-catenin stability and MMP-1 and MMP-9 expression and arresting the cell cycle. Right: mitochondrial apoptosis proceeding through the Apaf-1, caspase-9, and caspase-3 cascade. Green arrows denote an increase and red arrows a decrease in the abundance or activity of the indicated protein; red blunt-ended connectors denote inhibition and black arrows the direction of the cascade. Created in BioRender. Kowalczyk, T. (2026) https://BioRender.com/0ihpnh8.

To complement the studies discussed above and to allow direct comparison, Table 3 compiles further in vitro and in vivo investigations of celastrol and its derivatives in breast, ovarian, and cervical cancer. Each entry is presented in a uniform format that specifies the chemical compound tested, the study model and cell line, the targeted effect, the concentration or dose applied and the IC_50_ where available, and the observed activity, mechanism, or effect, so that the pharmacological profile of celastrol can be compared consistently across the three cancer types. The principal biological effects reported across these studies are summarised in Figure 4.

**Table 3:** In vitro and in vivo effects of celastrol on breast, ovarian, and cervical cancer models. Half-maximal inhibitory concentrations are reported as given in the original publications; values for the same cell line differ between studies according to exposure time, assay readout, and celastrol source, and should not be compared directly across rows. Endpoints are listed as examined in the source. Where a mechanism is given, it is the interpretation offered by the authors of that study, and the level of evidence supporting it ranges from the observation of a change in a pathway component to independent causal testing; the same grading of evidence applies to these entries as to Table 2.

Compound Tested	Model System and Cell Line	Endpoint Examined	Concentration or Dose; IC50 Where Reported	Observed Effect and Mechanism Proposed in the Source	Ref.
Breast cancer					
Celastrol obtained from *Tripterygium regelii*	Oestrogen receptor-positive (ER+) luminal breast cancer MCF-7 cells	Caspase activation; Apoptosis management (Bax/Bcl-2 regulation); Cell growth suppression; Intrinsic apoptotic pathway induction	Tested at 1, 3, 5 and 10 μM	Celastrol inhibited MCF-7 cell growth at 3 μM and higher concentration, induced apoptosis (through caspase-7/8/9 increased activation and Bax protein expression). Administration of celastrol also caused a release of cytochrome c and AIF from the mitochondria to the cytosol in tested cells. These findings suggest a potential use of celastrol for apoptosis induction by both caspase-dependent and -independent apoptotic pathways in breast cancer treatment.	[44]
Celastrol	In vitro: ER-positive luminal breast cancer cell lines MCF-7 and T47D In vivo: MCF-7 breast cancer xenografts in BALB/c nude mice	Anti-proliferative activity: inhibition of SGK3/STAT3/ZMIZ2/β-catenin signalling; increased β-catenin polyubiquitination	Tested at 1, 2, 4, 8 and 16 μM celastrol In vitro: IC_50_: 3.124 μM (MCF-7), 2.450 μM (T47D); In vivo:0.5 or 1 mg/kg i.p. daily for 21 days in BALB/c-nude mice	Tested compound inhibited breast cancer cell viability and colony formation in a dose-dependent manner. Proliferation was decreased through inhibition of SGK3/STAT3/ZMIZ2/β-catenin signalling in both models.	[125]
Celastrol	In vitro: ER-positive MCF-7 breast cancer cells In vivo: orthotopic MCF-7 xenografts in nude mice	Apoptosis induced through activation of endoplasmic reticulum stress	Dose not stated in the source	Celastrol activated endoplasmic reticulum stress and induced apoptosis in ER-positive breast cancer cells, and reduced the volume of orthotopic MCF-7 xenografts without a significant effect on body weight.	[155]
Celastrol	Triple-negative breast cancer (TNBC) cell lines MDA-MB-231 and MDA-MB-468	Cell toxicity; Metastasis prevention, Invasion and Migration reduction; Synergistic effect with docetaxel	Concentrations -Sub-toxic: 0.5–1 μM -Growth inhibition observed at ≥1.5 μM	Tested compound was found efficient in up-regulation of E-cadherin (involved in epithelial–mesenchymal transition (EMT)) and down-regulated Hakai (E3 ligase for E-cadherin degradation). Celastrol exerted a synergistic growth inhibitory effect with docetaxel. The findings suggest a potential application of celastrol for anti-metastatic, anti-migratory and anti-invasive effect on highly aggressive cancer cells, underlining its exceptional synergism with docetaxel in treatment of TNBC.	[135]
Celastrol (additionally: Withaferin A)	Highly invasive TNBC MDA-MB-231 cells	Cell toxicity; Colony formation ability; Wound healing; Cell death induction (TNF-α-induced apoptosis-synergistic action)	Celastrol: 0.25–8 μM; TNF-α: 10 ng/mL. IC_50_ = 2.0 μM (Cel alone) IC_50_ = 0.58 μM (Cel + TNF-α)	TNF-α combined with tested celastrol induced apoptosis through caspase-3/-9 activation, proteasome activity inhibition, PARP cleavage and suppression of NF-κB nuclear translocation. The synergistic action in apoptosis induction poses a potential for breast cancer cell death induction.	[137]
Celastrol	In vitro:ER-positive MCF-7 and TNBC MDA-MB-231 breast cancer cells; In vivo: MDA-MB-231 xenograft mouse model	Cell toxicity, mTOR activity modulation, effect of celastrol on cell-derived xenograft model	In vitro: 0.5–8 μM In vivo: 3 mg/kg intragastrically	In vitro testing showed that celastrol inhibited mTOR phosphorylation at Ser2481 and induced mTOR ubiquitination, causing proteasomal degradation. This disrupted mTOR-Hsp90 signalling and downregulated PI3K/Akt/mTOR signalling. The growth-inhibitory activity was subsequently confirmed in an orthotopic MDA-MB-231 xenograft.	[102]
Celastrol	Triple-negative breast cancer (TNBC) cells: Hs578T, HCC1143, MDA-MB-231, HCC1806, MDA-MB-157	Inflammatory response attenuation; IL-1β attenuation; Cell toxicity; Colony formation ability;	Used in IL-1β expression testing: 0.25–0.5 μM; Growth inhibition observed at 2–4 μM	Suppression of MEK/ERK-dependent pathway by celastrol used in study promoted IL-1β downregulation, leading to simultaneous decrease in IL-8, MMP-1 and MMP-9 expression. This activity creates a potential of celastrol in cancer cell migration inhibition and inflammatory response attenuation in triple negative breast cancer cells.	[98]
Celastrol	ER-positive MCF-7 cells, TNBC MDA-MB-231 and BT-549 cells, and non-tumorigenic MCF-10A mammary epithelial cells	Cytotoxicity, colony formation regulation, apoptosis induction, DNA fragmentation, Cell cycle arrest	Tested concentrations: 0.5–1.5 μM; PI3K enzyme inhibition: 500 nM; Cytotoxicity: 0–25 μM	Induced ROS generation, mitochondrial membrane depolarisation, DNA fragmentation, Bax upregulation, Bcl-2 downregulation, caspase-3 activation in the caspase-3-proficient triple-negative lines and PARP cleavage, caused by celastrol on cells used in study, led to induction of apoptosis. MCF-7 cells carry a deletion of CASP3 and do not express caspase-3, so the caspase-3 readouts apply to MDA-MB-231 and BT-549 only. Tested compound was confirmed to have suppressed PI3K/Akt/mTOR, Akt/GSK3β and NF-κB survival signalling (PI3K activity inhibited by ~73%).	[18]
Celastrol derivative 11i (CEL-pyrazine derivative; contains pyrazine scaffold in A/B ring and C-20 urea moiety with (1-methylpiperidin-4-yl) piperazine substitution)	In vitro: MCF-7 breast cancer cells; In vivo: tested in zebrafish xenograft model	Antiproliferative; Autophagy induction; Cell cycle arrest; modulation of LC3 A/B, p62 and Beclin-1 expression	IC_50_ = 1.31 μM against MCF-7 cells (whereas parent celastrol, IC_50_ = 2.23 μM)	The derivative 11i was found remarkably efficient in autophagy induction in breast cancer cells, via the regulation of LC3 A/B, p62 and Beclin-1 expression. Moreover, described derivative showed relatively low toxicity against human normal cell line L02 with an IC_50_ value of 4.68 μM, allowing for a conclusion that it exerts promising selectivity for cancer treatment. The findings were supported by In vivo testing, showing that the derivative could inhibit the proliferation of the MCF-7 cells.	[62]
Celastrol	ER-positive MCF-7 breast cancer cells	Suppression of viability via AMP-activated protein kinase (AMPK)-dependent fashion; p53 and PLK-2 pathways modulation	Significant viability reduction from 300 nM; Mechanistic studies mainly performed at 1 μM	Tested celastrol led to decrease in the viability of breast cancer MCF-7 cells in an AMP-activated protein kinase (AMPK)-dependent trend. Simultaneously, it increased ROS production, polo like kinase-2 (PLK-2) expression, and p53 phosphorylation. These findings suggest anti-cancer effect of celastrol via AMPK-induced p53 and PLK-2 pathways.	[105]
Celastrol	Luminal A breast cancer cell lines MCF-7, T47D, and ZR-75-1; HER2-overexpressing MDA-MB-453 breast cancer cells; TNBC cell lines HCC1143, HCC1806, MDA-MB-231, and Hs578T	Amphiregulin expression modulation; ERK phosphorylation control, EGFR signalling pathway suppression	Tested concentration: 0.25–0.5 μM used mechanistically; Cell viability began decreasing at ≥1 μM	Tested compound suppressed AREG (amphiregulin) expression, inhibited MEK/ERK signalling, reduced ERK phosphorylation, and reduced AREG expression and the growth of TNBC cells. It was observed that celastrol also suppressed the AREG-induced EGFR signalling pathway, showing a potential for anticancer and anti-angiogenic effect of celastrol in triple negative breast cancer.	[109]
Celastrol + Lapatinib (combination therapy)	HER2-overexpressing breast cancer MDA-MB-453 cells	Combination with lapatinib in cancer treatment; Apoptosis induction; Growth inhibition; HER2 membrane protein downregulation	Evaluated at 5 μM celastrol + 5 μM lapatinib; Synergy observed across 0.234–30 μM range	Combination treatment with celastrol and lapatinib produced strong synergistic cytotoxicity and apoptosis, activated caspase-3/9, downregulated HER2, p-HER2, p-Akt, and p-ERK1/2, and reduced HER2 membrane expression more effectively than either agent alone suggesting strong synergy of both drugs in the treatment of HER2neu-overexpressing cancer cells.	[120]
Celastrol	ER-positive MCF-7 breast cancer cells (and androgen-independent PC-3 prostate cancer cells)	miR-223 expression, NF-κB inhibition and transient mTOR activation induction;	IC_50_ approximately 4 μM (MCF-7) and 2 μM (PC-3) at 24 h; these concentrations were used in most mechanistic experiments	Celastrol induced miR-223 expression, inhibited NF-κB, transiently activated mTOR, and reduced viable cell numbers. Inhibition of miR-223 further enhanced celastrol-induced cell death, suggesting upregulation of miR-223 induced by celastrol provides a novel anticancer strategy.	[156]
Celastrol (CEL) (quinone methide triterpenoid) in combination with Tamoxifen (TAM)	In vitro: TNBC MDA-MB-231 cells In vivo: MDA-MB-231 xenografts in female BALB/c-nu mice	Combination of celastrol with tamoxifen; Mitochondria mediated apoptosis pathway	In vitro: CEL 1 μM (low) and 3 μM (high); TAM 0.5 μM (low) and 1 μM (high) In vivo: CEL 5 or 10 mg/kg and TAM 50 mg/kg, intraperitoneally once daily for 14 days	Tested compounds promoted mitochondria-mediated apoptosis, alongside ROS, Bax, cleaved caspase-3, cytochrome c upregulation and Bcl-2, mitochondrial membrane potential and GSH decrease; The findings of in vivo test show a suppression in tumour volume, altogether suggesting superior antitumour effect and tamoxifen chemosensitivity in TNBC in comparison to these compounds alone.	[123]
Celastrol (additionally: triptolide)	Triple-negative breast cancer (TNBC): MDA-MB-231, BT20, and patient-derived TNBC cells (PD-TNBC); mammosphere (CSC-enriched) cultures	Mammosphere formation suppressed; Reduced DCLK1, ALDH1, CD133, HES-1 and HEY-1 expression	Celastrol: 0.5–1 μM effective; Triptolide: 5–10 nM effective; Mammosphere assays used 0–12.5 μM celastrol and 0–125 nM triptolide	Tested compounds, including celastrol, were confirmed to suppress mammosphere growth in triple negative breast cancer models. Administration of celastrol decreased CSC markers DCLK1, ALDH1, CD133; inhibited Notch1 signalling (decreased Jagged1, NICD1, HES1, HEY1); reduced presenilin-1 and nicastrin expression, indicating inhibition of γ-secretase/Notch pathway and CSC self-renewal. These findings show a promising potential of celastrol in targeting stem cells and the Notch signalling in breast cancer treatment.	[116]
Celastrol	Human TNBC cell line MDA-MB-231	Regulation of HSDL2, the pro-apoptotic protein, Bcl-2-associated X (BaX), Bcl-2 expression; Apoptosis induction; HSDL2/MAPK/ERK signalling pathway management	IC_50_ = 1.445 μM (celastrol, 24 h)	Celastrol directly bound to and downregulated HSDL2, leading to increased expression of the pro-apoptotic protein Bax and decreased expression of the anti-apoptotic protein Bcl-2. This promoted apoptosis in breast cancer cells and was associated with inhibition of the MAPK/ERK signalling pathway through reduced ERK phosphorylation	[93]
Celastrol	ER-positive breast cancer cell lines MCF-7 and T47D	Human oestrogen receptor α expression regulation (mRNA and protein levels); Cell cycle arrest (sub G1); Proliferation suppression	ERα suppression observed at 2–5 μM; commonly treated with 5 μM celastrol	Tested compound downregulated ERα at both mRNA and protein levels and reduced ERα transcriptional activity in cell lines used in study. Celastrol also suppressed expression of ERα-target genes, including cyclin D_1_, progesterone receptor (PR), and c-Myb. These regulations resulted in cell cycle arrest (sub G1) and inhibition of oestrogen receptor-positive breast cancer cell proliferation.	[121]
Celastrol (in scope of leptin activity)	MCF-7 cells	Leptin-triggered cell proliferation and migration suppression; PI3K/AKT pathway inhibition	Used concentrations: 1 μM, 10 μM, 25 μM, 50 μM, 100 μM of celastrol for 24, 48, and 72 h. IC_50_ values of celastrol after 24, 48, and 72 h were 22.68 ± 1.46 μM, 7.56 ± 0.89 μM and 0.89 ± 0.067 μM	Tested celastrol reversed leptin-induced proliferation and migration by binding to the leptin receptor (LEPR) and inhibiting leptin-mediated activation of the PI3K/AKT pathway. Celastrol also shifted the expression of genes involved in lipid metabolism and endocrine signalling, showing a promising potential in breast cancer treatment.	[103]
Celastrol	Anoikis-resistant TNBC cell variants MDA-MB-231-AR and BT-549-AR	Growth and proliferation inhibition; Colony formation reduction; Invasion/Migration/Metastasis attenuation;	IC_50_ = 1.510 μM and 1.673 μM, for MDA-MB-231-AR cells and BT-549-AR respectively	Celastrol inhibited proliferation, migration, invasion, and tumour cell colony formation. Tested compound also led to promoted anoikis. Aforementioned effects were associated with inhibition of the EGFR/MEK/ERK signalling pathway, including reduced phosphorylation of EGFR, MEK, and ERK. This prompts an auspicious opportunity of using celastrol as anti-tumour and anti-metastatic agent.	[126]
Celastrol (additionally gambogic acid)	In vitro: Breast cancer cells expressing mutant ERα Y537S (MCF-7-ERα-Y537S, MDA-MB-231-ERα-Y537S); In vivo: xenograft model	Anti-proliferative and pro-apoptotic activity against endocrine-resistant breast cancer; Colony formation regulation.	Screening at 10 μM; combination studies with 10–300 nM celastrol and 10–1000 nM abemaciclib	Celastrol was identified as a novel antagonist of mutant ERα Y537S. Along with gambogic acid, it suppressed ERα/SRC1 interaction, inhibited ERα mutant signalling, and showed synergistic antiproliferative and pro-apoptotic effects when combined with CDK4/6 inhibitor abemaciclib, showing that tested synergism could perform as an alternative breast cancer treatment option.	[122]
Celastrol + Tamoxifen	In vitro: ER-positive breast cancer MCF-7 cells; In vivo: MCF-7 xenograft mouse model	Synergistic action with tamoxifen; Apoptosis induction; Cell cycle arrest (G1 phase); Autophagy Induction; In vivo tumour volume suppression	Celastrol IC_50_ = 1.77 μM (48 h); Tamoxifen IC_50_ = 7.87 μM; combination used 0.3 μM celastrol + 1–10 μM tamoxifen.	In used MCF-7 breast cancer cells, celastrol exerted tamoxifen-supported apoptosis induction and promoted autophagy. The treatment reduced phosphorylation of Akt and mTOR which led to increasing the autophagy associated proteins LC3II and P62. In vivo studies of celastrol showed that it contributed to reduced tumour growth and decreased p-Akt and p-mTOR expression.	[124]
Celastrol	MCF-7 breast cancer cells (PMA-induced invasion model)	Matrix Metalloproteinase-9-Expression regulation; invasion/migration management; anti-metastatic effect	Non-cytotoxic concentrations ≤0.5 μM inhibited invasion; 2.5 μM completely inhibited colony formation.	Tested celastrol inhibited breast cancer cell migration and invasion by suppressing MMP-9 expression at both mRNA and protein. The study results showed that it blocked NF-κB signalling by preventing IκB degradation, inhibiting NF-κB nuclear translocation, and reducing NF-κB binding to the MMP-9 promoter, resulting in decreased metastatic activity. These findings suggest effective anti-metastatic potential of celastrol.	[22]
Celastrol	In vitro: TNBC models: Murine TNBC cell lines 4T1 and E0771; In vivo: BALB/c mouse xenograft model	Activation of LKB1/AMPK signalling; suppression of PD-L1 and MYC expression; disruption of the PD-L1/MYC regulatory axis; inhibition of proliferation, migration, invasion, immune evasion, and tumour growth	In vitro concentration gradient 0–10 μM; in vivo 2 mg/kg every 2 days	Celastrol inhibited triple-negative breast cancer cell proliferation, invasion, migration, and immune evasion. It activated LKB1 and AMPK phosphorylation while reducing PD-L1 and MYC expression. Celastrol disrupted the PD-L1/MYC regulatory axis, leading to decreased MYC stability and reduced tumour growth. In vivo, celastrol decreased tumour volume and weight and increased p-LKB1 and p-AMPK levels while suppressing PD-L1 and MYC expression.	[99]
Celastrol	Human TNBC cell lines MDA-MB-231 and MDA-MB-468	Anti-metastatic effect; Tackling Invasion/Migration	Non-toxic range 0.1–1 μM Viability affected at 2–5 μM	Celastrol suppressed migration and invasion of triple-negative breast cancer cells by IL-6 expression attenuation at both mRNA and protein levels. It inhibited NF-κB signalling, resulting in downregulated IL-6 production. IL-6 overexpression attenuated the anti-metastatic effects of celastrol, indicating that inhibition of the NF-κB/IL-6 axis contributes to its activity.	[134]
Celastrol	Breast cancer: MCF-7; Colon cancer: DLD-1, RKO	Paraptosis induction; Endoplasmic reticulum stress (dilation); Mitochondrial Ca^2+^ accumulation	Used concentrations: 0.1, 0.5, 1, 2, 5 μM for 24 h	Celastrol induced paraptosis (cell death) by promoting endoplasmic reticulum stress, proteasome inhibition, and mitochondrial Ca^2+^ accumulation. It increased mitochondrial Ca^2+^ uptake through IP3R-mediated Ca^2+^ release from the endoplasmic reticulum, leading to dilation of the endoplasmic reticulum and mitochondria, accumulation of polyubiquitinated proteins.	[145]
Celastrol	MDA-MB-231 and MCF-7 breast cancer cells	Suppression of anti-apoptotic proteins (cIAP1, cIAP2, FLIP, Bcl-2); activation of caspase-8/caspase-3/PARP apoptotic cascade; inhibition of MMP-9-mediated invasion and metastasis	Tested at: 1–10 μM celastrol Apoptosis and invasion studies at 3–10 μM	Celastrol supported TNF-α-induced apoptosis by caspase-8 activation and PARP cleavage, with caspase-3 activation in MDA-MB-231 cells; MCF-7 cells do not express caspase-3. It reduced the expression of anti-apoptotic proteins cIAP1, cIAP2, FLIP, and Bcl-2. Celastrol also inhibited breast cancer cell invasion and suppressed TNF-α-induced MMP-9 expression at both mRNA and protein levels. This shows a favourable opportunity to administer celastrol in multifactorial cancer treatment.	[133]
Ovarian cancer					
Celastrol	OVCAR3 (High grade ovarian serous adenocarcinoma) and SKOV3 (Ovarian serous cystadenocarcinoma)	In vitro: Regulation of migration and NF-κB signalling pathway; Regulation of expression and activity of MMP-9	Tested at 0.125, 0.25, 0.5, 1 and 2 μM	Celastrol inhibited proliferation of both cell lines at concentrations of 1 μM or above. At subtoxic concentrations of 0.25 and 0.5 μM it inhibited invasion of OVCAR3 cells, with inhibition rates of 39.6 ± 5.2% and 51.7 ± 3.5% respectively, and inhibited migration of OVCAR3 cells in a wound-healing assay and of SKOV3 cells in a Transwell assay. Of the matrix metalloproteinases examined, only MMP-9 was downregulated, whereas MMP-2 and MMP-7 were unchanged.	[97]
Celastrol	A2780 ovarian cancer line	In vitro: Suppression of proliferation; Influence on Apoptosis induction and cell cycle; Core targets (MYC, CDC37, and FN1) regulation	Tested at 0.1, 0.5, and 1 μM	Celastrol suppressed the proliferation of ovarian cancer cell lines in a dose-dependent manner and caused the S phase cell cycle arrest in ovarian cancer cells. Tested compound was also found efficient in inhibition of MYC, CDC37, and FN1 mRNA expression.	[157]
Celastrol	OVCAR-8 (Human ovarian carcinoma cell line)	In vitro: Regulation of death receptor 4 and 5 expression; Supplementary activity for TRAIL/Apo-2L apoptosis regulation	Tested at 2.5 μM	Celastrol induced DR4 and DR5 up-regulation in tested cell line in a time-dependent manner-celastrol (2.5 μM) treatment for 240 min caused about 3.3-fold increment of DR4. In OVCAR-8 cells, p38 MAPK phosphorylation increased moderately upon celastrol exposure, with no dependency on TRAIL/Apo-2L-induced apoptosis.	[158]
Celastrol	OVCAR3 cells	In vitro: regulation of miRNA-21 expression, caspase-3/caspase-9 activity and PI3K/Akt-NF-κB signalling pathway	Tested at 0, 0.25, 0.5, 1, 2, 4 and 6 μM for 0, 1, 2 and 3 days.	Treatment with celastrol (2 and 4 μM) for 2 days was found efficient in reducing the protein expression levels of NF-κB, PI3K and p-Akt, along with reduction in miRNA-21 expression levels, suggesting potential apoptosis-inducing activity of celastrol	[159]
Celastrol and celastrol derivatives with cinnamamide chains (6a-6h: piperazine-linked; 7a-7i: aminoethyl-linked), especially compound **7g** (3,4,5-trimethoxy cinnamamide derivative)	Human ovarian cancer cell lines SKOV3, OVCAR3, and A2780 cell lines; ovarian CSC populations (CD44+, CD133+, ALDH+)	Synthesis: celastrol C-20 cinnamamide modifications. In vitro: Induction of apoptosis and suppression of ovarian cancer stem cell properties via regulation of apoptosis-related proteins and CSC markers (CD44+, CD133+ and ALDH+); suppression of spheroid formation;	IC_50_: 0.62 μM (SKOV3), 0.68 μM (OVCAR3), 0.65 μM (A2780) for derivative 7g (celastrol with 3,4,5-trimethoxycinnamamide side chain)	Compounds used in study increased Caspase-3 and Bax and simultaneously decreased Bcl-2 expression. 7g derivative was chosen the most optimal considering IC_50_ results and selectivity index (IC_50_ values on IOSE80 cells/SKOV3 cells). This derivative decreased the percentage of CD44+, CD133+ and ALDH+ cells. Tested derivatives of celastrol were found more potent than celastrol. Derivative 7g was found promising for potential apoptosis-induction, anti-migration and anti-proliferation properties.	[63]
Semisynthetic celastrol derivatives (C-20-substituted piperazine derivatives and A/B ring-modified derivatives; compounds **3**–**5**, **6a**–**6g**, **7a**–**7g**; most active compound: 6c, an isopropylpiperazine-substituted celastrol derivative)	Human ovarian cancer cells: SKOV3, A2780, OVCAR3; ovarian cancer stem cells (CSCs);	In vitro: inhibition of STAT3/p-STAT3 signalling pathway; suppression of spheroid formation and CSC markers (CD44+, CD133+ and ALDH+); mitochondrial membrane potential disruption and apoptosis induction via cleaved caspase-3 activation	IC_50_: SKOV3 = 0.47 μM, A2780 = 0.87 μM, OVCAR3 = 0.23 μM, IOSE80 = 2.30 μM for derivative 6c (containing an isopropylpiperazine substituent)	Derivatives showed potent anti-proliferation activity and selectivity toward tested ovarian cancer cells. Compound 6c was found the strongest in reducing CD44+, CD133+ and ALDH+ populations, suppressing spheroid formation, inducing apoptosis via cleaved caspase-3 and mitochondrial membrane potential disruption, and inhibiting STAT3/p-STAT3 signalling pathway, all collectively suggesting a multifaceted anticancer property.	[64]
Cel-N-C-20 celastrol derivative synthesised via condensation of celastrol carboxylic acid with piperazine	In vitro: Human ovarian cancer cell lines SKOV3, A2780, OVCAR3; ovarian cancer stem cells (CSCs); In vivo: SKOV3 xenograft mouse model	Regulation of STAT3/p-STAT, JAK1/JAK2, survivin, and Mcl-1 expression; G2/M cell cycle arrest; regulation of ROS formation; reduction in CD44+CD24-, ALDH+ CSCs, and spheroid formation; systemic toxicity and selectivity	In vitro: IC_50_: SKOV3 = 0.21 μM, A2780 = 0.14 μM, OVCAR3 = 0.25 μM; In vivo: doses of (2 mg/kg, 5 mg/kg) for 21 days (BALB/c nude mice)	Tested celastrol derivative exerted inhibitory effect on STAT3/p-STAT3 signalling. Cel-N derivative caused an induction of G2/M arrest and apoptosis through increasing caspase-3/8 activity, along with suppression of migration/invasion and CSC markers (CD44+CD24-, ALDH+). Cytotoxicity against ovarian cancer cells was stronger in comparison to normal IOSE80 cell line, suggesting a potential selectivity in cellular toxicity. In in vivo xenograft model testing supported aforementioned findings showing significant tumour inhibition alongside with minimal systemic toxicity.	[65]
Celastrol (commercial reagent, purity above 98%; Sigma-Aldrich)	Human ovarian cancer cells: SKOV3, A2780, OVCAR3;	Apoptosis and oncogenic pathway modulation-Modulation of Bax/Bcl-2, Akt, p-STAT3, p-P38, p-JNK, and P65 and caspase-3/8 activity; Migration prevention; Cell cycle arrest; Reduction in CSC markers (CD44, Nanog, Klf4, and Oct4) and downregulation of the CD44^high^CD24^low^ cell population.	Tested at 0, 0.25, 0.5, 1, 2 and 4 μM	Celastrol was found effective in causing G2/M cell cycle arrest with no influence on the G0/G1 phase. Tested compound was proven to induce apoptosis in cell lines in study, allegedly through Caspase-3, Caspase-8, and Bax increase and Bcl-2 downregulation. A suppression of Akt, p-STAT3, p-P38, p-JNK, and P65 was detected, showing that celastrol could be efficient in targeting multiple cancer driving pathways simultaneously.	[19]
Celastrol C-20 ester derivatives (most active: compound **24**)	Human ovarian cancer SKOV3; breast cancer SK-BR-3 and MDA-MB-231; non-tumour BJ fibroblasts	Cytotoxicity, tumour-versus-normal selectivity, cell cycle, apoptosis	IC_50_ (MTT, 72 h) for compound **24**: 0.56 μM (SKOV3), 0.78 μM (SK-BR-3), 1.15 μM (MDA-MB-231), 3.29 μM (BJ); celastrol: 1.16, 0.72, 1.32 and 2.74 μM respectively	Two-fold higher potency than celastrol against SKOV3 cells with an approximately six-fold selectivity over non-tumour fibroblasts; perturbation of the cell cycle and induction of apoptosis in SKOV3 cells	[66]
Celastrol C-20 carbamate derivatives (compounds **9** and **18**)	Human ovarian cancer SKOV3; breast cancer MCF-7 and MDA-MB-231; colorectal HT-29; non-tumour BJ fibroblasts	Cytotoxicity, tumour-versus-normal selectivity, clonogenic survival	IC_50_ (MTT, 72 h) for compound **18**: 0.54 μM (SKOV3), 1.68 μM (MCF-7), 1.35 μM (MDA-MB-231), 3.68 μM (BJ); compound **9**: 0.45, 0.59, 0.52 and 0.88 μM; celastrol: 1.16, 1.35, 1.32 and 2.74 μM respectively	Compound 18 combined submicromolar activity against SKOV3 cells with an approximately seven-fold margin over non-tumour fibroblasts and suppressed colony formation at twice its IC_50_; compound **9** was more potent across the panel but lost the selectivity margin	[67]
Cervical cancer					
Celastrol (commercial reagent; quinone methide triterpenoid of *Tripterygium wilfordii*)	Human cervical cancer HeLa cells	Redox-regulatory proteins (GSTO1, PDI, GR), heat-shock proteins, and the ubiquitin-proteasome pathway targeting (through reversible covalent binding to protein thiols)	HeLa: GI_50_ = 1.9 μM; target profiling and competition assays mainly performed at 10 μM celastrol	Celastrol forms covalent and reversible bonds with protein thiols, which leads to dysregulation of redox-regulatory proteins activity; affecting redox metabolism alongside with ubiquitin-proteasome pathway contributes potential apoptotic inducive properties. These findings suggest competitive chemoproteomic targeting assisted by celastrol as potential solution for anti-proliferative application on cervical cancer.	[94]
Celastrol	Human cervical cancer HeLa cells	Apoptosis regulation: mitochondrial dysfunction; caspase-3 activation; disruption of glycolysis, TCA cycle, amino acid, and nucleotide metabolism.	2.5–10 μM for 24–72 h; metabolomic studies mainly at 5 μM for 12 h;	Celastrol inhibited the growth of the tested cancer cells in a dose- and time-dependent manner. It has also induced apoptosis with caspase-3 activation and mitochondrial membrane depolarisation. The analysis of metabolomic features showed interruption of energy metabolism, amino acid biosynthesis, protein synthesis and nucleotide metabolism.	[21]
Celastrol C-20 amino acid ester derivatives (most active: compounds **2a** and **2e**)	Human cervical cancer HeLa cells; A549 lung and HepG-2 hepatoma cells as comparators	Cytotoxicity, apoptosis	IC_50_ (MTT) against HeLa: 0.371 μM (**2a**) and 0.237 μM (**2e**) versus 0.947 μM for celastrol; against A549: 0.235 and 0.109 μM versus 1.069 μM	Esterification of the C-20 carboxyl group with aliphatic amino acids increased potency against HeLa and A549 cells and induced early and late apoptosis in a concentration-dependent manner; substitution with aromatic amino acids abolished the gain	[68]

## 6. Utilisation of Celastrol in Drug Delivery Systems for Cancer Treatment

Nanocarriers address the specific liabilities of celastrol in three ways. They increase the dissolution rate and hence the bioavailability of a compound whose aqueous solubility is very low; they provide sustained and stimuli-dependent release that limits the peak plasma concentrations responsible for systemic toxicity; and they exploit features of the tumour microenvironment such as acidic extracellular pH, redox gradients, hypoxia, and elevated enzymatic activity to concentrate the compound in tumour tissue [160,161,162,163]. Selectivity is achieved either passively, through the enhanced permeability and retention effect, or actively, by decorating the carrier with ligands for receptors overexpressed on tumour cells, and is further modulated by particle size, charge, and surface chemistry [162,163]. That these principles apply to celastrol in particular has been demonstrated outside oncology, in orally administered pH-sensitive nanoparticles that release the compound in the inflamed colon in ulcerative colitis [164]. The principal safety caveat concerns the carriers themselves, since lipid-based nanoparticles may elicit complement-mediated hypersensitivity reactions, which must be taken into account in the design of clinically applicable formulations [165].

The physicochemical profile of celastrol, characterised by very low aqueous solubility, high lipophilicity, limited oral bioavailability, and a narrow therapeutic window, together with its dose-dependent hepatotoxicity, nephrotoxicity, and reproductive toxicity, constitutes the main obstacle to its therapeutic use [26,27]. Nanotechnology-based delivery systems have therefore been developed to improve the solubility and stability of celastrol, prolong its circulation time, increase selective accumulation in tumour tissue, and reduce the systemic exposure of healthy organs [16,28,29].

A wide range of carriers has been evaluated for celastrol in breast, ovarian, and cervical cancer, including liposomes and surface-modified liposomal systems, polymeric micelles, polymeric and lipid nanoparticles, self-assembled and carrier-free nanomedicines co-loading celastrol with a second therapeutic agent, gold-based and metal–organic framework platforms, and biomimetic constructs camouflaged with cell or bacterial membranes. These formulations frequently make use of features of the tumour microenvironment, such as reduced extracellular pH, elevated reactive oxygen species, or specific receptors, to achieve stimuli-responsive and targeted drug release, and several combine celastrol with chemotherapeutics, immunomodulators, or immune-checkpoint blockade to obtain additive or synergistic antitumour effects while lowering the effective dose. The nanotechnological formulations of celastrol reported in breast, ovarian, and cervical cancer models, together with their target applications, study models, doses, and observed benefits, are summarised in Table 4.

Two qualifications should be kept in view when reading the formulation literature summarised in Table 4, because the aggregate of that table is easily read as stronger than the individual studies warrant. The first concerns the direction of the comparison. Some reports measure improvement against free celastrol and others against an untreated or vehicle control, and in at least one case the formulation was less cytotoxic in vitro than the free compound, which the authors attributed to incomplete release [166]; that behaviour is the expected consequence of sustained release and is informative rather than discrepant, but it means that the comparator must be read from each row and not assumed. The second concerns the meaning of targeting, which is frequently inferred from carrier design and from cellular uptake in vitro, whereas quantitative biodistribution data exist for only a subset of the systems described. The enhanced permeability and retention effect on which passive targeting depends is moreover heterogeneous between patients and between tumour types, and is exaggerated in the rodent models in which these formulations were tested [160,162]. Reported selectivity should therefore be read as a property demonstrated in those models rather than as an established pharmacokinetic advantage.

Liposomes are by far the most frequently used carrier for celastrol and illustrate the full range of design strategies. The simplest formulations, built from soybean phospholipid and cholesterol, already improve bioavailability and reduce toxicity [167], but most reported systems add a functional surface. Glycyrrhizic acid modification reduces cytotoxicity towards normal mammary epithelium while promoting uptake by tumour cells [168], folate conjugation of a bilayer carrying celastrol together with irinotecan in the aqueous core produces pH-sensitive release of both drugs and higher tumour accumulation [169], and a micelle-in-liposome construct coated with folate achieves sequential release of two agents and, at 2 mg/kg, depletes cancer-associated fibroblasts and thereby increases drug penetration in triple-negative disease [170]. Liposomes are also the format in which the mechanistic themes outlined above reappear. Celastrol-loaded ginsenoside Rg3 liposomes given at 1 mg/kg induced immunogenic cell death and potentiated anti-programmed death-ligand 1 therapy [171], and liposomes co-encapsulating celastrol with triptolide behind an R8 transmembrane peptide and a red blood cell membrane coat prolonged circulation and triggered apoptosis at a 1:100 molar ratio of the two compounds [172]. Two constructs add an external trigger: gold nanorod-anchored thermosensitive liposomes release their payload on irradiation [173], and a furin-responsive liposomal complex shrinks on cleavage by the tumour protease to penetrate HeLa spheroids [174]. Two lipid microemulsions co-loading tripterine with coix seed oil complete the cervical set. Transferrin modification reduced the half-maximal inhibitory concentration against HeLa cells to 0.726 μmol/L, 2.58-fold below the unmodified carrier, raised cellular uptake 2.28-fold, and allowed the label to be detected 350 μm below the surface of three-dimensional HeLa spheroids [175]; in HeLa xenografts the same construct lowered the half-maximal inhibitory concentration of tripterine to 0.31 μg/mL with a combination index of 0.66, accumulated at the tumour, and at 1.8 mg/kg tripterine given every other day slowed tumour growth while reducing circulating transforming growth factor β1, CCL2, tumour necrosis factor α, and interleukin 6 [176].

Polymeric and carrier-free systems form the second group and address solubility and loading capacity more directly. Hyaluronic acid micelles exploit CD44 for targeting and release celastrol rapidly in the tumour microenvironment [177], whereas micelles of chitosan-stearic acid modified with the mitochondriotropic cation CTPP direct the compound to mitochondria [178], the organelle in which several of the direct targets of celastrol reside. Poly(lactic-co-glycolic acid) nanoparticles [179] and polymers bearing a pH-sensitive molecular switch [180] reduce systemic exposure, and high drug-loading nanosuspensions dispense with a carrier altogether [181]. The carrier-free principle has been extended to co-assembly of celastrol with a second active agent, which raises the drug fraction of the particle to near unity: celastrol with methotrexate [182], with Taxol on a chitosan oligosaccharide scaffold, where the combination reversed Taxol resistance in MCF-7/Taxol cells [183], and with resiquimod in a self-assembled core–shell particle that accumulated in mitochondria and, at 3 mg/kg celastrol with 2 mg/kg resiquimod, matched the tumour inhibition of paclitaxel with less systemic toxicity [184]. The group is completed by a lactoferrin dual-drug nanoconjugate [185] and by PEGylated liposomes encapsulating a celastrol–thalidomide chimera, which removed the need for toxic organic solvents and lowered the half-maximal inhibitory concentration in 4T1 cells from 1.770 to 1.257 μM [186].

Inorganic, metal–organic, and biomimetic platforms make up the third group and are the ones in which ovarian cancer is represented. Gold nanoparticles decorated with folic acid improve aqueous solubility and selectivity in two-dimensional and three-dimensional breast cancer models [187], and calcium carbonate cores within a glycyrrhetinic acid-modified liposomal shell add pH-dependent dissolution [188]. In ovarian cancer, celastrol has been loaded into nanoparticles self-assembled from the amphiphilic polymer PLGA-TK-PEG-FA, in which the thioketal linker responds to the elevated reactive oxygen species of the tumour [166], and into a biotin-decorated zeolitic imidazolate framework, ZIF-8, which released the compound at acidic pH and, at 2 mg/kg, suppressed growth of SKOV3 xenografts with a fall in mitochondrial membrane potential [189]. Biomimetic camouflage appears in two forms: the erythrocyte membrane used to prolong circulation [172], and a hybrid membrane fused from *Fusobacterium nucleatum* and PD-1-overexpressing T cells, which allowed celastrol at 2 mg/kg to clear intratumoural bacteria and kill tumour cells simultaneously without disrupting the gut microbiome [190].

Two limitations of this body of work should be stated. First, the distribution across the three cancers is extremely uneven: of the formulations compiled in Table 4, twenty were developed in breast cancer models, two in ovarian cancer, and three in cervical cancer. All three cervical formulations were reported between 2018 and 2020 and none since, all three come from the same group [174,175,176], so that the tumour type with the clearest mechanistic evidence for celastrol is the one with the least recent delivery work behind it. Second, the comparators differ so widely between studies, in cell line, exposure time, and whether free celastrol or a blank carrier serves as the control, that the reported gains in potency cannot be pooled or ranked across formulations.

Celastrol can also participate in the synthesis of its own carrier. Because the quinone methide of celastrol is itself a redox-active electrophile, the compound can serve as the reducing agent for gold nanoparticles that combine chemotherapy with photothermal anticancer therapy, so that the active compound and its carrier are generated in a single step [191]. Since celastrol is obtained in low yield from a slow-growing plant, routes of this kind bear directly on the cost structure of any future celastrol nanomedicine, a point taken up again in the discussion of economic considerations.

**Table 4:** Nanotechnological structures combined with celastrol in breast, ovarian, and cervical cancer. Entries are reported as stated in each source, and three qualifications apply throughout. First, improvements in potency or efficacy are relative to the comparator used in that same study, which is free celastrol in most cases and an untreated or vehicle control in others; they are not comparable between studies, because carrier composition, cell line, exposure time, and readout all differ, and a small number of formulations were less potent than free celastrol in vitro, which is the expected consequence of sustained release rather than a failure of the carrier. Second, the word targeting denotes the design intention of the formulation together with whatever uptake or biodistribution data the source reports; it does not imply that selective delivery to tumour tissue was demonstrated quantitatively, and for several of the systems listed no biodistribution data were reported at all. Third, formulations designated tripterine in the source are celastrol formulations and are identified as such here.

Nanocarrier and Composition	Design Objective Stated by the Authors	Target Study Model	Dose or Concentration	Reported Outcome and the Comparator Against Which it Was Measured	Ref.
Breast cancer					
Folic acid-modified celastrol gold nanoparticles	Increasing water solubility and tumour selection.	2D and 3D breast cancer model	Cytotoxicity assay: 0.078–20 μM Apoptosis and MMP: 2.5 μM	Synthesized material was highly soluble in aqueous mediums. Product structure allows for effective encapsulation and rapid release. High cellular uptake efficiency was also confirmed. Substance exhibited killing activity and apoptosis-inducing ability in breast cancer models.	[187]
GA-Modified Liposomal Calcium Carbonate Nanoparticles	Increasing solubility and stability.	4T1 mouse breast cancer cells	Cytotoxicity assay and Apoptosis: 0.125 to 32 μM IC_50_ for CEL, LCC-CEL, and GA-LCC-CEL were calculated to be 0.841 μM, 1.109 μM, 2.027 μM	Material was characterised by an appropriate size and charge, higher stability and sustained release properties. Using this formula leads to rapid release of CEL, GA, and Ca2+ and also can mitigate the damage to normal cells induced by celastrol.	[188]
Folate-coated micelle-in-liposome nanosystem	Overcoming drug resistance and inhibiting cancer growth.	In Vitro: 4T1 and NIH 3T3 cells. In Vivo: BALB/c mice with 4T1/NIH 3T3 breast tumours and lung metastases.	In Vitro: 0.025–1 μg/mL. In Vivo: 2 mg/kg.	Nanosystem effectively inhibits tumour growth and metastasis in TNBC. Sequential delivery of two drugs was achieved. CAFs were removed and drug penetration was increased.	[170]
Nanomedicine Based on Celastrol and Methotrexate	Increasing efficiency of Celastrol and overcoming toxicity problems	In Vitro: A549 (human lung cancer) and 4T1 (mouse breast cancer) cells. In Vivo: BALB/c mice bearing 4T1 tumour xenografts.	In Vitro: Celastrol (0.25–3 μg/mL) and Methotrexate (0.125–1.5 μg/mL). In Vivo: 3 mg/kg Celastrol and 1.5 mg/kg Methotrexate.	Formula combining Ce and MTX has excellent tumour-targeting ability and provides anticancer effects of the two drugs. Ce- MTX NPs efficiently released the drugs in acidic tumour environments. The circulation time was prolonged and accumulation at the tumour site was promoted. NPs improved the antitumour efficacy of both Ce and MTX and shown favourable biocompatibility.	[182]
Nanoparticles based on polymers modified with pH-sensitive molecular switch and low molecular weight heparin carrying Celastrol and ferrocene	Reducing hydrophobicity and high systemic toxicity.	In vitro: 4T1 mouse breast cancer cell line. In vivo: Female BALB/c mice bearing 4T1 orthotopic breast cancer tumours and pulmonary metastases.	In vitro: Celastrol, Celastrol + Ferrocene, PP NPs, and PP/H NPs concentrations: 10 to 500 ng/mL. In vivo: dose of 2 mg/kg (based on celastrol). IC_50_ (4T1 cells at 48 h) PP/H NPs: 20.37 ± 1.55 ng/mL. PP NPs: 22.96 ± 4.30 ng/mL. Celastrol + Ferrocene: 155.47 ± 5.14 ng/mL. Celastrol: 221.87 ± 14.86 ng/mL.	PP/H NPs successfully accumulated in tumours and released drug in response to the acidic lysosomal environment. Nanoparticles achieved a significant tumour inhibition rate and effectively inhibited pulmonary metastasis in vivo.	[180]
Dual-targeting co-delivery, self-assembled core–shell nanoparticles	Enhance the antitumour efficacy and minimise systemic toxicity.	In vitro: Murine breast cancer 4T1 cell line. In vivo: Female Balb/c mice bearing a 4T1 subcutaneous breast cancer model.	In vivo: H/TCR NPs delivering celastrol 3 mg/kg and R848 2 mg/kg, intravenously every 2 days (5 doses); paclitaxel injection 8 mg/kg served as the positive control arm and is not a component of the nanoparticle. *Hemolysis assay: celastrol-equivalent concentrations of 16, 31, 62, 125, 250 and 500 μg/mL* IC*_50_* *(4T1 cells at 48 h)* *H/TCR NPs: 0.54 μg/mL. Free celastrol: 0.51 μg/mL*.	H/TCR NPs exhibited stability and enhanced targeted accumulation in mitochondria. This dual-targeting capability promoted mitochondria-mediated apoptosis and resulted in high tumour inhibition in vivo with minimal systemic side effects.	[184]
Celastrol-chitosan oligosaccharide/Taxol nanoparticles	Overcoming drug resistance.	In Vitro: MCF-7 (human breast cancer cell line) and MCF-7/Taxol (Taxol-resistant human breast cancer cell line). In Vivo: Specific Pathogen Free (SPF) grade female BALB/c nude mice bearing MCF-7/Taxol subcutaneous xenograft tumours.	In Vitro: cells exposed to free Taxol, Cel-CSO, Taxol/Cel-CSO Mix and Cel-CSO/Taxol NPs for 24 h (concentration range not stated in the source). In Vivo*: 2 mg/kg Taxol and 4 mg/kg celastrol, intravenously every 2 days for 2 weeks.* *IC*_50_ *(MCF-7/Taxol cells at 24 h)* *Free Taxol: 252.40 ± 15.70 μg/mL. Cel-CSO: 3.25 ± 0.22 μg/mL. Taxol/Cel-CSO Mix: 1.56 ± 0.07 μg/mL. Cel-CSO/Taxol NPs: 1.08 ± 0.05 μg/mL.*	Cel-CSO/Taxol NPs exhibited stability, enhanced cellular accumulation and retention and improved drug release. They amplified intracellular reactive oxygen species and activated apoptosis pathway. Therapy inhibited tumour growth and reversed Taxol resistance and minimised systemic toxicity.	[183]
Modified celastrol-loaded polylactic acid glycolic acid nanoparticles	Increasing bioavailability and decreasing systemic toxicity.	In Vitro: 4T1 murine breast cancer cells. In Vivo: BALB/c mice bearing a subcutaneous 4T1 breast cancer model.	In Vitro: Concentrations up to 0.5 μg/mL. In Vivo: Dose of 2 mg/kg based on celastrol	Nanoparticles successfully facilitated targeted uptake by tumour cells. Celastrol was rapidly released in the alkaline mitochondrial environment. They achieved high tumour inhibition rate in vivo with excellent biocompatibility and reduced systemic toxicity	[179]
Hyaluronic acid-based polymeric micelles loaded with celastrol	Enhancing water solubility, bioavailability and decreasing systemic toxicity.	In Vitro: MCF-7 and 4T1 breast cancer cell lines. In Vivo: Female BALB/c mice bearing 4T1 subcutaneous breast tumours.	In Vitro: Celastrol at concentrations from 0.01 to 10 μg/mL. In Vivo: dose of 2 mg/kg based on celastrol	Structure allowed the micelles to target breast cancer cells and enabled rapid drug release in the tumour microenvironment. Celastrol circulation time was prolonged and bioavailability was increased. The formulation achieved high tumour inhibition rate, decreased tumour biomarkers and protected normal organs from damage.	[177]
Celastrol micelles composed of CTPP-modified chitosan-stearic acid	Improving delivery system and providing selective drug release.	In Vitro: MCF-7 (human breast adenocarcinoma), A549 (human non-small cell lung cancer), and L-02 (human normal liver) cell lines. In Vivo: Nude mice (6–8 weeks old) bearing MCF-7 tumour xenografts.	In Vitro: Concentration of celastrol ranged from 0.5 to 3 μg/mL In Vivo: Dose of 2 mg/kg IC_50_ (48 h) MCF-7 Cells: 1.81 ± 0.14 μg/mL for CTPP-CSOSA/Cela micelles. A549 Cells: 0.76 ± 0.03 μg/mL for CTPP-CSOSA/Cela micelles.	Micelles accumulated in tumour tissues. Rapid release in response to the mitochondrial matrix environment was achieved., This amplified reactive oxygen species, depolarized the mitochondrial membrane and induced apoptosis with low systemic toxicity.	[178]
High drug-loading celastrol nanosuspensions	Overcoming poor solubility, low bioavailability, and systemic toxicity.	In Vitro: Murine-derived breast cancer (4T1) cell line. In Vivo: Female BALB/c mice bearing 4T1 subcutaneous breast tumour xenografts.	In Vivo: Dose of 3 mg/kg continuously for 12 days IC_50_ (4T1 cells at 48 h) Free Celastrol (CSL): 4.11 μg/mL. CSL-NSps: 2.82 μg/mL	CSL-NSps achieved high encapsulation efficiency and drug loading content. The nanosuspensions demonstrated high stability, which makes them suitable for both oral and intravenous delivery routes. In vivo CSL-NSps achieved high tumour inhibition exhibiting better therapeutic efficacy compared to the standard treatment and demonstrated minimal systemic toxicity.	[181]
Liposomes co-encapsulating celastrol and triptolide, decorated with the R8 transmembrane peptide, fused with a red blood cell membrane shell	Improving delivery of the drugs.	In Vitro: HepG2 (human liver cancer) and MCF-7 (human breast cancer) cell lines, as well as RAW 264.7 macrophages. In Vivo: Male ICR mice bearing H22 subcutaneous liver tumours and female BALB/c nude mice bearing 4T1 subcutaneous breast tumours.	In Vitro: Celastrol at a 1:100 molar ratio (specifically, 0.01 μM TP and 1 μM Cel). In Vivo: Dose of 0.024 mg/kg for TP and 3 mg/kg for Cel. IC_50_ HepG2 cells: 1028 ± 8.50 nM for the TP/Cel mixture, compared to 1223 ± 7.49 nM for free Cel. MCF-7 cells: 1695 ± 7.50 nM for the TP/Cel mixture, compared to 2099 ± 7.08 nM for free Cel.	RBC membrane camouflage successfully prolonged blood circulation. High accumulation at the tumour site and efficient endocytosis of the liposomes by the tumour cells were achieved. This drug delivery system induced apoptosis, triggered autophagy and inhibited angiogenesis, resulting in inhibition of tumour growth in both liver and breast cancer models with low systemic toxicity.	[172]
Lactoferrin-dual drug nanoconjugate	Overcoming low drug loading, poor solubility and improving drug release.	In Vitro: MCF-7 human breast cancer cell line. In Vivo: Female BALB/C mice bearing Ehrlich Ascites Tumour (EAT) in the mammary fat pad.	In Vitro: Concentrations from 0 to 16 μM In Vivo: Dosage was 2 mg/kg of docetaxel and 2.5 mg/kg of celastrol.	Nanoconjugate achieved high drug loading without reducing stability. Cellular uptake was improved and premature drug leakage in the bloodstream was inhibited. Formula suppressed tumour growth and prolonged the survival rate.	[185]
PEGylated liposomes encapsulating a Celastrol–thalidomide chimera	Overcoming poor water solubility, low targeting efficiency, and systemic toxicity.	In Vitro: 4T1 murine triple-negative breast cancer (TNBC) cell line. In Vivo: Female BALB/c mice bearing 4T1 subcutaneous breast tumour xenografts.	In Vitro: Concentrations from 0.5 to 3 μM of the drug formulations. In Vivo: Dose of 6 mg/kg. IC_50_ (4T1 cells at 24 h) Celastrol-PROTACs solution: 1.770 μM. Lip-Celastrol-PROTACs: 1.257 μM.	Encapsulation of Celastrol-PROTACs eliminated the problem of using toxic organic solvents and enabled rapid cellular uptake. In vivo, prolonged circulation and enhanced tumour accumulation were achieved. Lip-Celastrol-PROTACs demonstrated higher tumour growth inhibition and reduced liver and kidney damage.	[186]
Celastrol-loaded ginsenoside Rg3 liposomes	Optimising pharmacokinetics and reducing systemic toxicity.	In Vitro: 4T1Fluc (luciferase-expressing mouse breast cancer) cells and MDA-MB-231 (human triple-negative breast cancer) cells. In Vivo: Female BALB/c mice bearing an orthotopic (in situ) 4T1Fluc breast cancer tumour model.	In Vitro: Immunogenic cell death markers assays: concentrations of 0.5 μM. In apoptosis assays: concentrations from 0 to 2.0 μM. In Vivo: Dosage of 1 mg/kg and a ginsenoside Rg3 dosage of 2 mg/kg Anti-programmed death-ligand 1 antibody dose: 5 mg/kg IC_50_ (4T1 cells at 48 h) Rg3-Lp (Blank Vector): 307.1 μM. Cel-Lp (Cholesterol Liposomes): 0.91 μM. Cel-Rg3-Lp (Rg3 Liposomes): 0.70 μM.	Cel-Rg3-Lp nanoparticles demonstrated tumour-targeting capability. The targeted delivery of celastrol induced immunogenic cell death successfully. Using this formula enhanced the therapeutic efficacy of aPD-L1 checkpoint immunotherapy without any noticeable biosafety hazards.	[171]
Glycyrrhizic acid-modified celastrol liposomes	Improving solubility and reducing systemic toxicity.	In Vitro: BT-549 (human breast cancer) and MCF-10A (human normal mammary epithelial) cell lines.	In Vitro: concentrations from 0.125 to 32 μM IC_50_ (BT-549 cells at 24 h) Free-CEL: 3.20 μM. LIP-CEL: 5.35 μM. GA/LIP-CEL: 12.85 μM.	Liposomes were stable and exhibited high encapsulation efficiency and a sustained drug release. The GA modification lowered cytotoxicity of celastrol toward normal cells and promoted cellular uptake breast cancer cells. The formulation demonstrated significant antitumour effects in vitro and activated inner apoptosis pathways.	[168]
Dual–drug-loaded liposomes containing celastrol in the lipid bilayer and irinotecan in the aqueous core	Improving solubility and drug delivery.	In Vitro: Folate receptor-positive human breast cancer cell lines (MCF-7 and MDA-MB-231) and a folate receptor-negative human lung cancer cell line (A549). In Vivo: Female BALB/c nude mice bearing MDA-MB-231 subcutaneous breast tumour xenografts.	In Vitro: Drug concentrations from 0.01 to 100 μg/mL	Liposomes demonstrated a rapid, pH-sensitive release of both drugs. Folic acid conjugation enabled better targeting and higher cellular uptake and apoptosis. Liposomes showed higher tumour accumulation and effectively inhibited tumour growth and significantly mitigated the systemic toxicity of free drugs.	[169]
Celastrol-loaded liposomes formulated using soybean phospholipid, cholesterol, and celastrol	Improving bioavailability and reducing toxicity.	In Vitro: 4T1 (murine triple-negative breast cancer) cells and 3T3-L1 In Vivo: Female BALB/c mice bearing an orthotopic 4T1 breast tumour model	In Vitro: Concentrations from 0.1 to 6.4 μmol/L In Vivo: Dose of 1 mg/kg (based on celastrol) Anti-PD-L1: dose of 5 mg/kg. IC_50_ (at 24 h) 4T1 tumour cells: 0.795 μmol/L for Cel/Lip (compared to 1.718 μmol/L for free celastrol). 3T3-L1 adipocytes: 0.816 μmol/L for Cel/Lip (compared to 1.047 μmol/L for free celastrol).	Cel/Lip nanoparticles showed excellent stability and a pH-sensitive release. In vivo, Cel/Lip amplified the antitumour response and achieved high tumour inhibition rate. Formula demonstrated high biosafety profile.	[167]
Gold nanorod-anchored thermo-sensitive liposomes co-loaded with STS and celastrol	Improving selective delivery.	In Vitro: MCF-7 human breast cancer cells. In Vivo: BALB/c nude mice bearing MCF-7 subcutaneous breast tumour xenografts.	In Vitro: Varying concentrations of celastrol, up to 20 μM In Vivo: Dose of 5 mg/kg for sodium tanshinone IIA sulfonate and 2 mg/kg for celastrol.	Gold nanorod-anchored liposomes converted near-infrared light into mild heat, which disrupted the liposomal membrane and triggered the rapid release of STS from the core. Celastrol was released more slowly in the next stage. STS remodeled the tumour microenvironment. This resulted in tumour shrinkage and apoptosis without damage associated with free celastrol.	[173]
Celastrol-loaded PLGA nanoparticles with a hybrid membrane fused from *Fusobacterium nucleatum* bacteria and PD-1-overexpressing CTLL-2 T cells	Extending targeting and eradicating tumour-promoting bacteria.	In Vitro: 4T1 (triple-negative breast cancer) cells, CTLL-2 (PD-1-overexpressing T cells) and *Fusobacterium nucleatum* bacteria. In Vivo: Female BALB/c mice bearing orthotopic 4T1 tumours implanted in the mammary fat pad, with intratumoural inoculation of *F. nucleatum* (10 μL, 10^6 CFU/mL) on day 7.	In Vitro: 0.25 to 128 μg/mL in the antibacterial assay against *F. nucleatum.* In Vivo: 2 mg/kg celastrol, intravenously, every 4 days (4 doses) Minimum inhibitory concentration of celastrol against *F. nucleatum:* 32 μg/mL. Concentration used for the antitumour effect: 1.25 μg/mL.	Celastrol cleared the intratumoural bacteria and showed direct cytotoxicity against the tumour cells. In vivo formula achieved high tumour suppression rate and demonstrated biosafety without disrupting the healthy gut microbiome.	[190]
Ovarian cancer					
Celastrol-loaded nanoparticles assembled from the amphiphilic polymer PLGA-TK-PEG-FA	Improve water solubility and tumour targeting.	In Vitro: SKOV3 human ovarian cancer cell line.	In Vitro: free celastrol and celastrol-loaded nanoparticles at 0, 0.1, 0.3, 1, 3 and 10 μg/mL (celastrol equivalent). IC_50_ (SKOV3 cells at 24 h) Free celastrol: approximately 1.4 μg/mL. No IC_50_ was calculated for the nanoparticles; at the highest tested concentration of 10 μg/mL SKOV3 viability was 48.3%, so the nanoparticles were slightly less cytotoxic than free celastrol at 24 h, which the authors attributed to incomplete release.	Nanoparticles targeted tumour cells and rapidly released celastrol in response to tumour environment. Celastrol amplified cellular oxidative stress, causing tumour cell apoptosis, and inhibited the migration of ovarian cancer cells.	[166]
Biotin decorated celastrol-loaded ZIF-8 nano-drug delivery system	Improving water solubility and reducing systemic side effects.	In Vitro: SKOV3 and ES-2 human epithelial ovarian cancer cell lines, and IOSE80 normal human ovarian epithelial cells. In Vivo: Female BALB/c nude mice bearing SKOV3 subcutaneous xenograft tumours.	In Vitro: Tested concentrations of 0.3125, 0.625, 1.25, and 2.5 μg/mL. In Vivo: Doses: cisplatin (DDP) at 2 mg/kg, celastrol (CEL) at 2 mg/kg, and CEL@ZIF-8@PEG-BIO at 2 mg/kg.	Nanoparticles improved water solubility, achieved pH-responsive drug release, and provided targeted endocytosis. Mitochondrial membrane potential declined and apoptosis pathways were activated. Formulation inhibited tumour growth and eliminated severe toxicity.	[189]
Cervical cancer					
Furin-responsive triterpenine-based liposomal complex	Optimising particle size to achieve tumour accumulation and deep tumour penetration.	In Vitro: HeLa (human cervical cancer) cell lines and HeLa three-dimensional (3D) tumour spheroids. In Vivo: BALB/c nude mice bearing HeLa subcutaneous xenograft tumours.	In Vitro: 0.625 to 20 μg/mL for 2D cytotoxicity assays (24 h); the concentration range used for the 3D spheroid assay is not stated in the source. In Vivo: 1.5 mg/kg (tripterine equivalent) intraperitoneally every 2 days, followed 6 h later by local hyperthermia in a 42 °C water bath for 1 h. IC_50_ HeLa cells (2D, 24 h): 0.9601 ± 0.02 μg/mL for PEG-cleavable Tf-CTM/L at 42 °C. HeLa 3D tumour spheroids: 150.6 ± 1.1 μg/mL for PEG-cleavable Tf-CTM/L at 42 °C.	Complex achieved high tumour accumulation and tumour penetration. This delivery strategy achieved high tumour inhibition rate in vivo and minimised systemic toxicity.	[174]
Transferrin-modified tripterine (celastrol)-loaded coix seed oil microemulsion	Improving tumour-specific accumulation and intratumoural penetration.	Human cervical cancer HeLa cells; three-dimensional HeLa tumour spheroids	IC_50_ 0.726 μM against HeLa cells	Particle size 40.02 ± 0.21 nm and zeta potential −13.63 ± 1.31 mV; 2.58-fold lower IC_50_ and 2.28-fold higher cellular uptake than the unmodified microemulsion; label detected 350 μm below the surface of the spheroid; 1.73-fold higher apoptotic rate than the unmodified carrier, downregulation of antiapoptotic proteins and arrest in G2/M.	[175]
Transferrin-functionalised microemulsion co-loading coix seed oil and tripterine (celastrol)	Deep tumour penetration and combined delivery of two agents.	HeLa cells; HeLa xenograft-bearing nude mice	IC_50_ 0.31 ± 0.06 μg/mL for tripterine and 7.65 ± 1.55 μg/mL for coix seed oil; in vivo tripterine 1.8 mg/kg with coix seed oil 44.4 mg/kg every other day	Combination index 0.66, indicating synergy; tumour accumulation and deep penetration into tumour tissue; slowed tumour growth, inhibited angiogenesis and induced apoptosis through bax/bcl-2 and caspase-3; serum transforming growth factor β1, CCL2, tumour necrosis factor α and interleukin 6 reduced by 27.7, 26.9, 61.2 and 42.5%; little toxicity in vital organs.	[176]

## 7. Economic Considerations and Translational Value

The three women’s cancers considered in this review impose a substantial and rising economic burden that provides part of the rationale for developing cost-effective, multi-targeted therapeutics such as celastrol. For breast cancer, the most frequently diagnosed malignancy in women, the global value of welfare lost to the disease was estimated at approximately 2539 billion United States dollars in 2021, corresponding to about 1.65% of global gross domestic product, with a marked regional redistribution of this burden projected by 2050 [192]. For cervical cancer, the global cumulative direct medical cost between 1991 and 2021 was estimated at 9.26 billion United States dollars without discounting and at 7.21 billion United States dollars when discounted to 2021, a burden concentrated in less developed regions [193]. In ovarian cancer, a multinational analysis across eleven countries estimated socioeconomic losses of approximately 70 billion United States dollars in 2023, of which about 91% reflected the value of lives lost and about 7% direct health expenditure, with treatment costs in the first two years after diagnosis representing many multiples of per-capita health spending, especially in lower-income settings [194]. These figures underline the value of therapies that are both effective and economically sustainable.

Natural products remain a valuable source of lead compounds for anticancer drug development, since 63.1% of all small-molecule drugs approved between 1981 and 2019 can be traced back to natural origins, although their structural complexity and pharmacokinetic behaviour complicate large-scale production. Celastrol has been highlighted as a representative example of the transformation of a traditional herbal constituent into a modern drug candidate, and emerging target-discovery strategies have progressively clarified its multi-target pharmacology and broadened its developmental value [195]. Bibliometric analysis of the celastrol literature published between 1997 and 2023 confirms sustained and accelerating growth in the annual volume of research, reflecting expanding scientific interest in this scaffold and the resources devoted to it [196]. A single molecule that engages several oncogenic pathways may, in principle, reduce reliance on multi-drug combinations, although this potential must be confirmed in controlled pharmacoeconomic evaluations.

A specific economic constraint concerns the supply of celastrol itself. The compound is obtained mainly by extraction from the roots and root bark of *T. wilfordii* in low yield, and its availability is limited by the slow growth and restricted distribution of the source plants. A biotechnological route has been established in which the celastrol biosynthetic pathway was elucidated and reconstructed by combining yeast-based metabolic engineering with chemical synthesis, enabling the scalable production of celastrol from sucrose [197]. Together with the use of celastrol itself as the reducing agent in nanoparticle synthesis, this addresses the raw-material and manufacturing costs that would otherwise limit the economic feasibility of celastrol-based products.

The economics of nanomedicine temper these opportunities. Nanocarriers can improve the therapeutic index of celastrol and reduce the dose and the toxicity-related costs of treatment, and earlier nanoparticle drugs have reached the market, with fifteen nanomedicines approved and 2018 sales of 252 million United States dollars for Doxil and 950 million for Abraxane, although Doxil improved safety rather than efficacy [160]. At the same time, the clinical translation of nanomedicines is more complex and more expensive than that of conventional formulations, and difficulties in reproducible large-scale manufacturing and quality control, together with effectiveness that sometimes falls below expectations, are repeatedly identified as major barriers to commercialisation [160]. The translation of celastrol nanoformulations from preclinical results into affordable clinical products will therefore depend on the combination of sustainable and scalable production of the active compound, robust and economically viable manufacturing of the carrier, and formal pharmacoeconomic assessment, none of which has yet been established for this agent.

## 8. Conclusions, Limitations and Future Perspectives

Celastrol emerges from the accumulated preclinical evidence as a potent, multi-targeted natural compound with consistent anticancer activity across breast, ovarian, and cervical cancer models. Its effects are mediated by the induction of apoptosis and cell-cycle arrest, the generation of reactive oxygen species and mitochondrial dysfunction, the suppression of migration, invasion, and metastasis, and the simultaneous modulation of interconnected signalling networks, including NF-κB, PI3K/Akt/mTOR, JAK/STAT3, MAPK/ERK, and Wnt/β-catenin, as well as targeted degradation of oncogenic client proteins. Structural modification of the C-20 carboxyl group and the A and B ring system has yielded derivatives, including cinnamamide and piperazine analogues and proteolysis-targeting chimeras, with improved potency and selectivity, while a diverse array of nanocarriers has been shown to overcome the poor solubility, low bioavailability, and systemic toxicity that limit the parent compound.

Several limitations must be acknowledged. The current evidence is almost entirely preclinical and derives from heterogeneous cell lines, animal models, doses, and treatment schedules, which complicate direct comparison and quantitative synthesis. Celastrol retains a narrow therapeutic window and dose-dependent hepatotoxicity, nephrotoxicity, and reproductive toxicity, and clinical pharmacokinetic and safety data are lacking. For the nanotechnological systems, questions of long-term safety, reproducible large-scale manufacturing, standardisation, and cost-effectiveness remain unresolved, and the potential toxicity and immunogenicity of the carriers themselves require further study. This review is narrative in scope, although the literature search on which it rests was systematic. Two features of that design limit what the synthesis can support. Records whose methodological reporting was too limited to yield the descriptors used in the tables were excluded, and because negative findings tend to be reported more briefly, this filter is unlikely to have operated symmetrically with respect to the direction of results. And the catalogue of molecular targets was assembled almost entirely from models outside breast, ovarian, and cervical cancer, so its application to these three tumours is an extrapolation rather than a finding of this review.

Future work should focus on the rational design of celastrol derivatives with a wider therapeutic window, on targeted and stimuli-responsive nanocarriers that maximise tumour selectivity, and on combinations of celastrol with conventional chemotherapy, targeted agents, or immunotherapy. Rigorous pharmacokinetic, toxicological, and, ultimately, clinical studies are needed to confirm the preclinical findings, and these should be accompanied by sustainable and scalable production of the compound and by formal pharmacoeconomic evaluation. Addressing these priorities in an integrated manner would allow the multi-targeted potential of celastrol to be translated into safe, effective, and economically viable therapies for women’s cancers.

## Data Availability

No new data were created or analyzed in this study. Data sharing is not applicable to this article.

## References

[1] Asma S.T., Acaroz U., Imre K., Morar A., Shah S.R.A., Hussain S.Z., Arslan-Acaroz D., Demirbas H., Hajrulai-Musliu Z., Istanbullugil F.R. (2022). Natural products/bioactive compounds as a source of anticancer drugs. Cancers.

[2] Newman D.J., Cragg G.M. (2020). Natural Products as Sources of New Drugs over the Nearly Four Decades from 01/1981 to 09/2019. J. Nat. Prod..

[3] Dhyani P., Quispe C., Sharma E., Bahukhandi A., Sati P., Attri D.C., Szopa A., Sharifi-Rad J., Docea A.O., Mardare I. (2022). Anticancer potential of alkaloids: A key emphasis to colchicine, vinblastine, vincristine, vindesine, vinorelbine and vincamine. Cancer Cell Int..

[4] Sofias A.M., Dunne M., Storm G., Allen C. (2017). The battle of “nano” paclitaxel. Adv. Drug Deliv. Rev..

[5] Bailly C. (2019). Irinotecan: 25 years of cancer treatment. Pharmacol. Res..

[6] Wang C., Dai S., Zhao X., Zhang Y., Gong L., Fu K., Ma C., Peng C., Li Y. (2023). Celastrol as an emerging anticancer agent: Current status, challenges and therapeutic strategies. Biomed. Pharmacother..

[7] Yang J., Liu J., Li J., Jing M., Zhang L., Sun M., Wang Q., Sun H., Hou G., Wang C. (2022). Celastrol inhibits rheumatoid arthritis by inducing autophagy via inhibition of the PI3K/AKT/mTOR signaling pathway. Int. Immunopharmacol..

[8] Astry B., Venkatesha S.H., Laurence A., Christensen-Quick A., Garzino-Demo A., Frieman M.B., O’Shea J.J., Moudgil K.D. (2015). Celastrol, a Chinese herbal compound, controls autoimmune inflammation by altering the balance of pathogenic and regulatory T cells in the target organ. Clin. Immunol..

[9] Cascão R., Fonseca J.E., Moita L.F. (2017). Celastrol: A Spectrum of Treatment Opportunities in Chronic Diseases. Front. Med..

[10] Lu Y., Liu Y., Zhou J., Li D., Gao W. (2021). Biosynthesis, total synthesis, structural modifications, bioactivity, and mechanism of action of the quinone-methide triterpenoid celastrol. Med. Res. Rev..

[11] Hong Z., Cao J., Liu D., Liu M., Chen M., Zeng F., Qin Z., Wang J., Tao T. (2023). Celastrol targeting Nedd4 reduces Nrf2-mediated oxidative stress in astrocytes after ischemic stroke. J. Pharm. Anal..

[12] Meng Z., Liu Q., Chen H., She C., Huang Y. (2025). Therapeutic potential of celastrol in bacterial infections: Current research advancements and future perspectives. Pharmacol. Res..

[13] Narayan V., Ravindra K.C., Chiaro C., Cary D., Aggarwal B.B., Henderson A.J., Prabhu K.S. (2011). Celastrol Inhibits Tat-Mediated Human Immunodeficiency Virus (HIV) Transcription and Replication. J. Mol. Biol..

[14] Tseng C.-K., Hsu S.-P., Lin C.-K., Wu Y.-H., Lee J.-C., Young K.-C. (2017). Celastrol inhibits hepatitis C virus replication by upregulating heme oxygenase-1 via the JNK MAPK/Nrf2 pathway in human hepatoma cells. Antiviral Res..

[15] Liu D., Zhang Q., Luo P., Gu L., Shen S., Tang H., Zhang Y., Lyu M., Shi Q., Yang C. (2022). Neuroprotective Effects of Celastrol in Neurodegenerative Diseases-Unscramble Its Major Mechanisms of Action and Targets. Aging Dis..

[16] Shi J., Li J., Xu Z., Chen L., Luo R., Zhang C., Gao F., Zhang J., Fu C. (2020). Celastrol: A Review of Useful Strategies Overcoming its Limitation in Anticancer Application. Front. Pharmacol..

[17] Zhu Y., Meng Y., Zhang J., Liu R., Shen S., Gu L., Wong Y.-K., Ma A., Chai X., Zhang Y. (2024). Recent Trends in anti-tumor mechanisms and molecular targets of celastrol. Int. J. Biol. Sci..

[18] Shrivastava S., Jeengar M.K., Reddy V.S., Reddy G.B., Naidu V.G.M. (2015). Anticancer effect of celastrol on human triple negative breast cancer: Possible involvement of oxidative stress, mitochondrial dysfunction, apoptosis and PI3K/Akt pathways. Exp. Mol. Pathol..

[19] Li X., Wang H., Ding J., Nie S., Wang L., Zhang L., Ren S. (2019). Celastrol strongly inhibits proliferation, migration and cancer stem cell properties through suppression of Pin1 in ovarian cancer cells. Eur. J. Pharmacol..

[20] Xu L.-N., Zhao N., Chen J.-Y., Ye P.-P., Nan X.-W., Zhou H.-H., Jiang Q.-W., Yang Y., Huang J.-R., Yuan M.-L. (2019). Celastrol Inhibits the Growth of Ovarian Cancer Cells in vitro *and* in vivo. Front. Oncol..

[21] Hu Y., Qi Y., Liu H., Fan G., Chai Y. (2013). Effects of celastrol on human cervical cancer cells as revealed by ion-trap gas chromatography–mass spectrometry based metabolic profiling. Biochim. Biophys. Acta Gen. Subj..

[22] Kim Y., Kang H., Jang S.-W., Ko J. (2011). Celastrol Inhibits Breast Cancer Cell Invasion via Suppression of NF-κB-mediated Matrix Metalloproteinase-9 Expression. Cell. Physiol. Biochem..

[23] Kaushal J.B., Shukla V., Sankhwar P., Jha R.K., Dwivedi A. (2021). Targeted inhibition of TAK1 abrogates TGFβ1 non-canonical signaling axis, NFκB/Smad7 inhibiting human endometriotic cells proliferation and inducing cell death involving autophagy. Cytokine.

[24] Alhakamy N.A., Alamoudi A.J., Abdulaal W.H., Choudhry H., Asfour H.Z., Al-Rabia M.W., Ashour O.M., Abdel-Naim A.B., Hofni A., Aboubakr E.M. (2025). Celastrol protects against cisplatin-induced ovarian toxicity via modulation of PPAR-γ and AMPK. Naunyn Schmiedeberg’s Arch. Pharmacol..

[25] Xiao Y., Li X., Mao J., Zheng H., Ji R., Wang Z., Guo M., Yuan H., Anwar A., Chen C. (2022). Reverse anti-breast cancer drug resistance effects by a novel two-step assembled nano-celastrol medicine. Nanoscale.

[26] He P., Sun J., Tan J., Shi Y.-N., Zhang C.-J., Zhu N., Yang Q., Qin L. (2025). From Physicochemical Constraints to Clinical Prospects of Celastrol: Challenges and Nano Delivery Strategies. Int. J. Nanomed..

[27] Jin C., Wu Z., Wang L., Kanai Y., He X. (2019). CYP450s-Activity Relations of Celastrol to Interact with Triptolide Reveal the Reasons of Hepatotoxicity of *Tripterygium wilfordii*. Molecules.

[28] Sun Y., Wang C., Li X., Lu J., Wang M. (2024). Recent advances in drug delivery of celastrol for enhancing efficiency and reducing the toxicity. Front. Pharmacol..

[29] Xu K., Wang Y., Wang H., Sun X., Yang Z. (2026). Overcoming the Druggability Hurdles of Celastrol: A Critical Review of Advanced Drug Delivery Strategies. Biomolecules.

[30] Fang G., Tang B. (2020). Current advances in the nano-delivery of celastrol for treating inflammation-associated diseases. J. Mater. Chem. B.

[31] Zhao Y., Miettinen K., Kampranis S.C. (2024). Celastrol: A century-long journey from the isolation to the biotechnological production and the development of an antiobesity drug. Curr. Opin. Plant Biol..

[32] Ma J.-S., Funston A.M., Wu Z.-Y., Raven P.H., Hong D.-Y. (2008). *Tripterygium wilfordii* J. D. Hooker. Flora of China.

[33] Shan Y., Zhao J., Wei K., Jiang P., Xu L., Chang C., Xu L., Shi Y., Zheng Y., Bian Y. (2023). A comprehensive review of *Tripterygium wilfordii* hook f in the treatment of rheumatic and autoimmune diseases: Bioactive compounds, mechanisms of action, and future directions. Front. Pharmacol..

[34] Tao X., Lipsky P.E. (2000). The Chinese anti-inflammatory and immunosuppressive herbal remedy *Tripterygium wilfordii* Hook F. Rheum. Dis. Clin. N. Am..

[35] Chen S.-R., Dai Y., Zhao J., Lin L., Wang Y., Wang Y. (2018). A Mechanistic Overview of Triptolide and Celastrol, Natural Products from *Tripterygium wilfordii* Hook F. Front. Pharmacol..

[36] Zhang Y., Mao X., Li W., Chen W., Wang X., Ma Z., Lin N. (2021). *Tripterygium wilfordii*: An inspiring resource for rheumatoid arthritis treatment. Med. Res. Rev..

[37] Jin H.Z., Hwang B.Y., Kim H.S., Lee J.H., Kim Y.H., Lee J.J. (2002). Antiinflammatory Constituents of *Celastrus orbiculatus* Inhibit the NF-κB Activation and NO Production. J. Nat. Prod..

[38] Wu J., Zhou Y., Wang L., Zuo J., Zhao W. (2012). Terpenoids from root bark of *Celastrus orbiculatus*. Phytochemistry.

[39] Luo D.-Q., Wang H., Tian X., Shao H.-J., Liu J.-K. (2005). Antifungal properties of pristimerin and celastrol isolated from *Celastrus hypoleucus*. Pest Manag. Sci..

[40] Morita H., Hirasawa Y., Muto A., Yoshida T., Sekita S., Shirota O. (2008). Antimitotic quinoid triterpenes from *Maytenus chuchuhuasca*. Bioorg. Med. Chem. Lett..

[41] Shirota O., Morita H., Takeya K., Itokawa H., Iitaka Y. (1994). Cytotoxic aromatic triterpenes from *Maytenus ilicifolia* and *Maytenus chuchuhuasca*. J. Nat. Prod..

[42] Filho W.B., Corsino J., Bolzani V.D.S., Furlan M., Pereira A.M.S., França S.C. (2002). Quantitative determination of cytotoxic Friedo-nor-oleanane derivatives from five morphological types of *Maytenus ilicifolia* (celastraceae) by reverse-phase high-performance liquid chromatography. Phytochem. Anal..

[43] Malaník M., Treml J., Rjašková V., Tížková K., Kaucká P., Kokoška L., Kubatka P., Šmejkal K. (2019). *Maytenus macrocarpa* (Ruiz & Pav) Briq: Phytochemistry and Pharmacological Activity. Molecules.

[44] Yang H.-S., Kim J.-Y., Lee J.-H., Lee B.-W., Park K.-H., Shim K.-H., Lee M.-K., Seo K.-I. (2011). Celastrol isolated from *Tripterygium regelii* induces apoptosis through both caspase-dependent and -independent pathways in human breast cancer cells. Food Chem. Toxicol..

[45] Moola A.K., Senthil Kumar T., Ranjitha Kumari B.D. (2022). Enhancement of Celastrol compound by silver nanoparticles and acetosyringone in *Celastrus paniculatus* Willd through adventitious and hairy root culture. J. Plant Biochem. Biotechnol..

[46] Venkatesha S.H., Yu H., Rajaiah R., Tong L., Moudgil K.D. (2011). *Celastrus*-derived Celastrol Suppresses Autoimmune Arthritis by Modulating Antigen-induced Cellular and Humoral Effector Responses. J. Biol. Chem..

[47] Niero R., de Andrade S.F., Cechinel Filho V. (2011). A Review of the Ethnopharmacology, Phytochemistry and Pharmacology of Plants of the *Maytenus* Genus. Curr. Pharm. Des..

[48] Ramadwa T.E., Meddows-Taylor S. (2025). A Review of the Phytochemistry, Molecular Docking, Pharmacology, Toxicology, Ethnopharmacology, Botany, and Clinical Studies of *Maytenus senegalensis* (Lam.) Excell. Biomolecules.

[49] POWO Plants of the World Online. Facilitated by the Royal Botanic Gardens, Kew. https://powo.science.kew.org.

[50] International Plant Names Index (IPNI). The Royal Botanic Gardens Kew, Harvard University Herbaria & Libraries and Australian National Herbarium. http://www.ipni.org.

[51] Mu X.-Y., Zhao L.-C., Zhang Z.-X. (2017). Molecular Analysis of Chinese *Celastrus* and *Tripterygium* and Implications in Medicinal and Pharmacological Studies. PLoS ONE.

[52] Chen X.-C., Lu Y., Liu Y., Zhou J.-W., Zhang Y.-F., Gao H.-Y., Li D., Gao W. (2022). Identification of a cytochrome P450 from *Tripterygium hypoglaucum* (Levl) Hutch that catalyzes polpunonic acid formation in celastrol biosynthesis. Chin. J. Nat. Med..

[53] Salminen A., Lehtonen M., Paimela T., Kaarniranta K. (2010). Celastrol: Molecular targets of Thunder God Vine. Biochem. Biophys. Res. Commun..

[54] Venkatesha S.H., Moudgil K.D. (2016). Celastrol and Its Role in Controlling Chronic Diseases. Adv. Exp. Med. Biol..

[55] National Center for Biotechnology Information PubChem Compound Summary for CID 122724, Celastrol. https://pubchem.ncbi.nlm.nih.gov/compound/122724.

[56] Jin T., Wu D., Liu X.-M., Xu J.-T., Ma B.-J., Ji Y., Jin Y.-Y., Wu S.-Y., Wu T., Ma K. (2020). Intra-articular delivery of celastrol by hollow mesoporous silica nanoparticles for pH-sensitive anti-inflammatory therapy against knee osteoarthritis. J. Nanobiotechnol..

[57] Sethi G., Ahn K.S., Pandey M.K., Aggarwal B.B. (2007). Celastrol, a novel triterpene, potentiates TNF-induced apoptosis and suppresses invasion of tumor cells by inhibiting NF-κB–regulated gene products and TAK1-mediated NF-κB activation. Blood.

[58] Gu Y., Yang M., Wang W., Li L., Ma Y., Liu W., Zhao Q. (2025). YX-112, a novel celastrol-derived PROTAC, inhibits the development of triple-negative breast cancer by targeting the degradation of multiple proteins. Front. Pharmacol..

[59] Gan X., Wang F., Luo J., Zhao Y., Wang Y., Yu C., Chen J. (2024). Proteolysis Targeting Chimeras (PROTACs) based on celastrol induce multiple protein degradation for triple-negative breast cancer treatment. Eur. J. Pharm. Sci..

[60] Hou W., Liu B., Xu H. (2020). Celastrol: Progresses in structure-modifications, structure-activity relationships, pharmacology and toxicology. Eur. J. Med. Chem..

[61] Zhang H.-J., Zhang G.-R., Piao H.-R., Quan Z.-S. (2018). Synthesis and characterisation of celastrol derivatives as potential anticancer agents. J. Enzyme Inhib. Med. Chem..

[62] Feng Y., Zhang B., Lv J., Zhang P., Mao Q., Lin F., Zhao J., Fu X., Yang Y., Li Z. (2022). Scaffold hopping of celastrol provides derivatives containing pepper ring, pyrazine and oxazole substructures as potent autophagy inducers against breast cancer cell line MCF-7. Eur. J. Med. Chem..

[63] Li X., Ding J., Li N., Liu W., Ding F., Zheng H., Ning Y., Wang H., Liu R., Ren S. (2019). Synthesis and biological evaluation of celastrol derivatives as anti-ovarian cancer stem cell agents. Eur. J. Med. Chem..

[64] Li N., Li C., Zhang J., Jiang Q., Wang Z., Nie S., Gao Z., Li G., Fang H., Ren S. (2022). Discovery of semisynthetic celastrol derivatives exhibiting potent anti-ovarian cancer stem cell activity and STAT3 inhibition. Chem. Biol. Interact..

[65] Liu M., Li N., Wang Z., Wang S., Ren S., Li X. (2024). Synthesis of a celastrol derivative as a cancer stem cell inhibitor through regulation of the STAT3 pathway for treatment of ovarian cancer. RSC Med. Chem..

[66] Figueiredo S.A.C., Salvador J.A.R., Cortés R., Cascante M. (2017). Novel celastrol derivatives with improved selectivity and enhanced antitumour activity: Design, synthesis and biological evaluation. Eur. J. Med. Chem..

[67] Figueiredo S.A.C., Salvador J.A.R., Cortés R., Cascante M. (2017). Design, synthesis and biological evaluation of novel C-29 carbamate celastrol derivatives as potent and selective cytotoxic compounds. Eur. J. Med. Chem..

[68] Pang C., Luo J., Liu C., Wu X., Wang D. (2018). Synthesis and biological evaluation of a series of novel celastrol derivatives with amino acid chain. Chem. Biodivers..

[69] Klaić L., Morimoto R.I., Silverman R.B. (2012). Celastrol analogues as inducers of the heat shock response. Design and synthesis of affinity probes for the identification of protein targets. ACS Chem. Biol..

[70] Boridy S., Le P.U., Petrecca K., Maysinger D. (2014). Celastrol targets proteostasis and acts synergistically with a heat-shock protein 90 inhibitor to kill human glioblastoma cells. Cell Death Dis..

[71] Zhang D., Chen Z., Hu C., Yan S., Li Z., Lian B., Xu Y., Ding R., Zeng Z., Zhang X.-K. (2018). Celastrol binds to its target protein via specific noncovalent interactions and reversible covalent bonds. Chem. Commun..

[72] Chan C.L., Sun Y., Mao X., Chen H., Zhao J., Rong J. (2024). Druggability, molecular targets, and nanocarrier delivery of natural triterpenoid celastrol against chronic diseases. Acta Mater. Med..

[73] Song J., He G.-N., Dai L. (2023). A comprehensive review on celastrol, triptolide and triptonide: Insights on their pharmacological activity, toxicity, combination therapy, new dosage form and novel drug delivery routes. Biomed. Pharmacother..

[74] Jiang Q., Liu Z., Cheng J., Geng Z., Gu Y., Wei Y., Yu M., Bi Y. (2026). The antitumor potential of celastrol: Research progress on antitumor mechanisms and strategies for toxicity reduction with efficacy enhancement. Biomolecules.

[75] Yang H., Chen D., Cui Q.C., Yuan X., Dou Q.P. (2006). Celastrol, a triterpene extracted from the Chinese “Thunder of God Vine,” is a potent proteasome inhibitor and suppresses human prostate cancer growth in nude mice. Cancer Res..

[76] Feng L., Zhang D., Fan C., Ma C., Yang W., Meng Y., Wu W., Guan S., Jiang B., Yang M. (2013). ER stress-mediated apoptosis induced by celastrol in cancer cells and important role of glycogen synthase kinase-3β in the signal network. Cell Death Dis..

[77] Lee J.-H., Koo T.H., Yoon H., Jung H.S., Jin H.Z., Lee K., Hong Y.-S., Lee J.J. (2006). Inhibition of NF-κB activation through targeting IκB kinase by celastrol, a quinone methide triterpenoid. Biochem. Pharmacol..

[78] Zhang T., Li Y., Yu Y., Zou P., Jiang Y., Sun D. (2009). Characterization of celastrol to inhibit Hsp90 and Cdc37 interaction. J. Biol. Chem..

[79] Sreeramulu S., Gande S.L., Göbel M., Schwalbe H. (2009). Molecular mechanism of inhibition of the human protein complex Hsp90–Cdc37, a kinome chaperone-cochaperone, by triterpene celastrol. Angew. Chem. Int. Ed..

[80] Peng B., Gu Y.-J., Wang Y., Cao F.-F., Zhang X., Zhang D.-H., Hou J. (2016). Mutations Y493G and K546D in human HSP90 disrupt binding of celastrol and reduce interaction with Cdc37. FEBS Open Bio.

[81] Westerheide S.D., Bosman J.D., Mbadugha B.N.A., Kawahara T.L.A., Matsumoto G., Kim S., Gu W., Devlin J.P., Silverman R.B., Morimoto R.I. (2004). Celastrols as inducers of the heat shock response and cytoprotection. J. Biol. Chem..

[82] Xu H., Zhao H., Ding C., Jiang D., Zhao Z., Li Y., Ding X., Gao J., Zhou H., Luo C. (2023). Celastrol suppresses colorectal cancer via covalent targeting peroxiredoxin 1. Signal Transduct. Target. Ther..

[83] Chen X., Zhao Y., Luo W., Chen S., Lin F., Zhang X., Fan S., Shen X., Wang Y., Liang G. (2020). Celastrol induces ROS-mediated apoptosis via directly targeting peroxiredoxin-2 in gastric cancer cells. Theranostics.

[84] Luo P., Liu D., Zhang Q., Yang F., Wong Y.-K., Xia F., Zhang J., Chen J., Tian Y., Yang C. (2022). Celastrol induces ferroptosis in activated HSCs to ameliorate hepatic fibrosis via targeting peroxiredoxins and HO-1. Acta Pharm. Sin. B.

[85] Lin T., Yang S.-L., Chen C.-J., Liu P.-F. (2025). Unveiling the potential binding targets of celastrol in colorectal cancer: A proteomic profiling approach integrating cellular thermal shift assay and pulse proteolysis. J. Proteome Res..

[86] Luo P., Zhang Q., Zhong T.-Y., Chen J.-Y., Zhang J.-Z., Tian Y., Zheng L.-H., Yang F. (2022). Celastrol mitigates inflammation in sepsis by inhibiting the PKM2-dependent Warburg effect. Mil. Med. Res..

[87] Shi Q.-L., Feng C.-R., Li H.-Y., Wang P.-L., Chen P., Wei X., Kuang W.-H., Li G.-J., Qin S.-J., Liu R. (2025). Celastrol inhibits the DPYSL2-JAK/STAT pathway by targeting mito-IDHs mediated mitochondrial metabolism to exhaust breast cancer. Acta Pharmacol. Sin..

[88] Yang F., Zhou H., Luo P., Jia L., Hou M., Huang J., Gao L., Zhang Q., Guan Y., Bao H. (2024). Celastrol induces DNA damage and cell death in BCR-ABL T315I-mutant CML by targeting YY1 and HMCES. Phytomedicine.

[89] Ni Z., Shi Y., Liu Q., Wang L., Sun X., Rao Y. (2024). Degradation-based protein profiling: A case study of celastrol. Adv. Sci..

[90] Wang H., Teriete P., Hu A., Raveendra-Panickar D., Pendelton K., Lazo J.S., Eiseman J., Holien T., Misund K., Oliynyk G. (2015). Direct inhibition of c-Myc-Max heterodimers by celastrol and celastrol-inspired triterpenoids. Oncotarget.

[91] Shirai T., Nakai A., Ando E., Fujimoto J., Leach S., Arimori T., Higo D., van Eerden F.J., Tulyeu J., Liu Y.-C. (2023). Celastrol suppresses humoral immune responses and autoimmunity by targeting the COMMD3/8 complex. Sci. Immunol..

[92] Kong Q., Liu S., He S., Luo Z., Lei R., Wang R., Liu X., Wu J. (2025). Celastrol enhanced CD8+ T cell immunity in melanoma by targeting SHP2 and upregulating MHC-I. Phytomedicine.

[93] Liu L., Liu Y., Zhang S., Zhang J., Meng Y., Liu D., Gu L., Zhang Y., Xu L., Zhang Z. (2024). Celastrol promotes apoptosis of breast cancer MDA-MB-231 cells by targeting HSDL2. Acupunct. Herb. Med..

[94] Zhou Y., Li W., Wang M., Zhang X., Zhang H., Tong X., Xiao Y. (2017). Competitive profiling of celastrol targets in human cervical cancer HeLa cells via quantitative chemical proteomics. Mol. Biosyst..

[95] Cho O., Lee J.-W., Jeong Y.-J., Kim L.K., Jung B.-K., Heo T.-H. (2024). Celastrol, which targets IL-2/CD25 binding inhibition, induces T cell-mediated antitumor activity in melanoma. Eur. J. Pharmacol..

[96] Yang J., He B., Dang L., Liu J., Liu G., Zhao Y., Yu P., Wang Q., Wang L., Xin W. (2025). Celastrol regulates the Hsp90-NLRP3 interaction to alleviate rheumatoid arthritis. Inflammation.

[97] Wang Z., Zhai Z., Du X. (2017). Celastrol inhibits migration and invasion through blocking the NF-κB pathway in ovarian cancer cells. Exp. Ther. Med..

[98] You D., Jeong Y., Yoon S., Kim S.A., Kim S., Nam S., Lee J., Kim S. (2021). Celastrol attenuates the inflammatory response by inhibiting IL-1β expression in triple-negative breast cancer cells. Oncol. Rep..

[99] Shi M., Li Y., Jin W., Xiong Y. (2026). Celastrol inhibits the immune escape and proliferation of triple-negative breast cancer (TNBC) through the LKB1–AMPK–PD-L1/MYC signaling pathway. Int. Immunopharmacol..

[100] Wang X.N., Wu Q., Yang X., Zhang L.S., Wu Y.P., Lu C. (2010). Effects of celastrol on growth inhibition of U937 leukemia cells through the regulation of the Notch1/NF-κB signaling pathway in vitro. Chin. J. Cancer.

[101] Kannaiyan R., Manu K.A., Chen L., Li F., Rajendran P., Subramaniam A., Lam P., Kumar A.P., Sethi G. (2011). Celastrol inhibits tumor cell proliferation and promotes apoptosis through the activation of c-Jun N-terminal kinase and suppression of PI3K/Akt signaling pathways. Apoptosis.

[102] Li X., Zhu G., Yao X., Wang N., Hu R., Kong Q., Zhou D., Long L., Cai J., Zhou W. (2018). Celastrol induces ubiquitin-dependent degradation of mTOR in breast cancer cells. Onco Targets Ther..

[103] Chen P., Wang B., Li M., Cui C., Liu F., Gao Y. (2022). Celastrol inhibits the proliferation and migration of MCF-7 cells through the leptin-triggered PI3K/AKT pathway. Comput. Struct. Biotechnol. J..

[104] Omer A.B., Afzal M., Saleem S., Khan M.M.U., Rekha A., Sharma R. (2026). Celastrol at the obesity–cancer interface: Critical appraisal of molecular mechanisms, preclinical evidence, and translational barriers. Mol. Biol. Rep..

[105] Kim J.H., Lee J.O., Lee S.K., Kim N., You G.Y., Moon J.W., Sha J., Kim S.J., Park S.H., Kim H.S. (2013). Celastrol suppresses breast cancer MCF-7 cell viability via the AMP-activated protein kinase (AMPK)-induced p53–polo like kinase 2 (PLK-2) pathway. Cell. Signal..

[106] Wang S., Ma K., Zhou C., Wang Y., Hu G., Chen L., Li Z., Hu C. (2019). LKB1 and YAP phosphorylation play important roles in celastrol-induced β-catenin degradation in colorectal cancer. Ther. Adv. Med. Oncol..

[107] Li G., Zhou L., Deng H., Huang C., Wang N., Yue L., Zhang P., Zhou Y. (2023). Targeting OPA1-mediated mitochondrial fusion contributed to celastrol’s anti-tumor angiogenesis effect. Pharmaceutics.

[108] Li Z., Xi R., Han X., Zhao P. (2026). Research on the molecular mechanism of celastrol targeting CTNNB1/STAT3 to inhibit uveal melanoma based on network pharmacology and multi-omics analysis. Sci. Rep..

[109] Kim S., You D., Jeong Y., Yoon S.Y., Lo E., Lee J.E. (2022). Celastrol attenuates amphiregulin expression by inhibiting MAPK signaling pathway in triple-negative breast cancer cells. Phytomedicine Plus.

[110] Wang W.-B., Feng L.-X., Yue Q.-X., Wu W.-Y., Guan S.-H., Jiang B.-H., Yang M., Liu X., Guo D.-A. (2012). Paraptosis accompanied by autophagy and apoptosis was induced by celastrol, a natural compound with influence on proteasome, ER stress and Hsp90. J. Cell. Physiol..

[111] Li H.-F., Zhu N., Wu J.-J., Shi Y.-N., Gu J., Qin L. (2024). Celastrol elicits antitumor effects through inducing immunogenic cell death and downregulating PD-L1 in ccRCC. Curr. Pharm. Des..

[112] Wang S.R., Hu G.H., Chen L.C., Ma K., Hu C.F., Zhu H.X., Xu N.Z., Zhou C.C., Liu M. (2023). Celastrol acts as a new histone deacetylase inhibitor to inhibit colorectal cancer cell growth via regulating macrophage polarity. Cell Biol. Int..

[113] Nouri K., Azad T., Ling M., Janse van Rensburg H.J., Pipchuk A., Shen H., Hao Y., Zhang J., Yang X. (2019). Identification of celastrol as a novel YAP-TEAD inhibitor for cancer therapy by high throughput screening with ultrasensitive YAP/TAZ–TEAD biosensors. Cancers.

[114] Wang S., Ma K., Chen L., Zhu H., Liang S., Liu M., Xu N. (2016). TAZ promotes cell growth and inhibits celastrol-induced cell apoptosis. Biosci. Rep..

[115] Salama M.M., Zaghloul R.A., Khalil R.M., El-Shishtawy M.M. (2025). Anti-neoplastic activity of celastrol in experimentally-induced mammary adenocarcinoma in mice: Targeting Wnt/β-catenin signaling pathway. Naunyn Schmiedeberg’s Arch. Pharmacol..

[116] Ramamoorthy P., Dandawate P., Jensen R.A., Anant S. (2021). Celastrol and Triptolide Suppress Stemness in Triple Negative Breast Cancer: Notch as a Therapeutic Target for Stem Cells. Biomedicines.

[117] Pei L., Zhu Y., Li W., Xu B., Sun J. (2025). Celastrol promotes DNA damage and apoptosis in uterine corpus endometrial carcinoma via promotion of KAT2B-mediated RBPJ acetylation and repression of MCM4 transcription. Mol. Med..

[118] Li D., Chen J., Ye C., Lin B., Zhang T., Chen Q., Yu C., Wan X. (2025). Celastrol ameliorates fibrosis in Western diet/tetrachloromethane-induced nonalcoholic steatohepatitis by suppressing Notch/osteopontin signaling. Phytomedicine.

[119] Raja S.M., Clubb R.J., Ortega-Cava C., Williams S.H., Bailey T.A., Duan L., Zhao X., Reddi A.L., Nyong A.M., Natarajan A. (2011). Anticancer activity of Celastrol in combination with ErbB2-targeted therapeutics for treatment of ErbB2-overexpressing breast cancers. Cancer Biol. Ther..

[120] Yan Y.-Y., Bi H., Zhang W., Wen Q., Liu H., Li J.-X., Zhang H.-Z., Zhang Y.-X., Li J.-S. (2017). Downregulation and subcellular distribution of HER2 involved in MDA-MB-453 breast cancer cell apoptosis induced by lapatinib/celastrol combination. J. BUON.

[121] Jang S.Y., Jang S.-W., Ko J. (2011). Celastrol inhibits the growth of estrogen positive human breast cancer cells through modulation of estrogen receptor α. Cancer Lett..

[122] Liu X., Hu Q., Wang W., Ma H., Pu J., Cui J., Gong T., Wu Y., Lu W., Huang J. (2021). A protein-fragment complementation assay reveals that celastrol and gambogic acid suppress ERα mutants in breast cancer. Biochem. Pharmacol..

[123] Qi B., Yan Q., Zhang P., Ren Y., Liu H., Liu T., Zhu L., Chen Y. (2023). Celastrol enhances tamoxifen sensitivity in the treatment of triple negative breast cancer via mitochondria mediated apoptosis pathway. Am. J. Transl. Res..

[124] Wang L., Tang L., Yao C., Liu C., Shu Y. (2021). The Synergistic Effects of Celastrol in combination with Tamoxifen on Apoptosis and Autophagy in MCF-7 Cells. J. Immunol. Res..

[125] Yuan L., Cai Y., Wang G., Liu X., Chen B., Zhou D., Wu Y., Qu N., Li X., Zhou W. (2025). SGK3 promotes estrogen receptor-positive breast cancer proliferation by activating STAT3/ZMIZ2 pathway to stabilise β-catenin. Br. J. Pharmacol..

[126] Yang J., Wang X., Bai X., Deng R., Ye W., Liu Q., Xing Y., Yao Y., Lyu A., Lu C. (2026). Celastrol increases anoikis sensitivity to suppress triple-negative breast cancer via EGFR pathway and p-EMT state regulation. Front. Pharmacol..

[127] Pang X., Yi Z., Zhang J., Lu B., Sung B., Qu W., Aggarwal B.B., Liu M. (2010). Celastrol suppresses angiogenesis-mediated tumor growth through inhibition of AKT/mammalian target of rapamycin pathway. Cancer Res..

[128] Zhu Y., Liu X., Zhao P., Zhao H., Gao W., Wang L. (2020). Celastrol suppresses glioma vasculogenic mimicry formation and angiogenesis by blocking the PI3K/Akt/mTOR signaling pathway. Front. Pharmacol..

[129] Ma J., Han L.Z., Liang H., Mi C., Shi H., Lee J.J., Jin X. (2014). Celastrol inhibits the HIF-1α pathway by inhibition of mTOR/p70S6K/eIF4E and ERK1/2 phosphorylation in human hepatoma cells. Oncol. Rep..

[130] Han X., Sun S., Zhao M., Cheng X., Chen G., Lin S., Guan Y., Yu X. (2014). Celastrol stimulates hypoxia-inducible factor-1 activity in tumor cells by initiating the ROS/Akt/p70S6K signaling pathway and enhancing hypoxia-inducible factor-1α protein synthesis. PLoS ONE.

[131] Jia L., Zhu S., Zhu M., Nie R., Huang L., Xu S., Luo Y., Su H., Huang S., Tan Q. (2024). Celastrol inhibits angiogenesis and the biological processes of MDA-MB-231 cells via the DEGS1/S1P signaling pathway. Biol. Chem..

[132] Zhang T., Zhao Q., Xiao X., Yang R., Hu D., Zhu X., Gonzalez F.J., Li F. (2019). Modulation of lipid metabolism by celastrol. J. Proteome Res..

[133] Mi C., Shi H., Ma J., Han L.Z., Lee J.J., Jin X. (2014). Celastrol induces the apoptosis of breast cancer cells and inhibits their invasion via downregulation of MMP-9. Oncol. Rep..

[134] Yan F., Wu Z., Li Z., Liu L. (2020). Celastrol Inhibits Migration and Invasion of Triple-Negative Breast Cancer Cells by Suppressing Interleukin-6 via Downregulating Nuclear Factor-κB (NF-κB). Med. Sci. Monit..

[135] Liu Z., Fan M., Xuan X., Xia C., Huang G., Ma L. (2022). Celastrol inhibits the migration and invasion and enhances the anti-cancer effects of docetaxel in human triple-negative breast cancer cells. Med. Oncol..

[136] Ren B., Liu H., Gao H., Liu S., Zhang Z., Fribley A.M., Callaghan M.U., Xu Z., Zeng Q., Li Y. (2017). Celastrol induces apoptosis in hepatocellular carcinoma cells via targeting ER-stress/UPR. Oncotarget.

[137] Lu L., Shi W., Deshmukh R.R., Long J., Cheng X., Ji W., Zeng G., Chen X., Zhang Y., Dou Q.P. (2014). Tumor Necrosis Factor-α Sensitizes Breast Cancer Cells to Natural Products with Proteasome-Inhibitory Activity Leading to Apoptosis. PLoS ONE.

[138] Ebrahimnezhad M., Valizadeh A., Yousefi B., Shanehbandi D. (2026). Celastrol overcomes 5-fluorouracil resistance in osteosarcoma cells through p53-mediated apoptotic pathway modulation and P-glycoprotein inhibition: A comprehensive mechanistic study. BioImpacts.

[139] Guo J., Cui D., Tang Y., Wang S., Ma C., Ma W. (2024). Celastrol activates caspase-3/GSDME-dependent pyroptosis in tumor cells by inducing endoplasmic reticulum stress. J. Tradit. Chin. Med. Sci..

[140] Guo D., Zhang W., Yang H., Bi J., Xie Y., Cheng B., Wang Y., Chen S. (2019). Celastrol induces necroptosis and ameliorates inflammation via targeting biglycan in human gastric carcinoma. Int. J. Mol. Sci..

[141] Zhang X., Qi M., Huo K., Cai B., Zhang J., Tian Y., Zhang D. (2024). Celastrol induces ferroptosis by suppressing RRM2 in hepatocellular carcinoma. Heliyon.

[142] Liu M., Fan Y., Li D., Han B., Meng Y., Chen F., Liu T., Song Z., Han Y., Huang L. (2021). Ferroptosis inducer erastin sensitizes NSCLC cells to celastrol through activation of the ROS–mitochondrial fission–mitophagy axis. Mol. Oncol..

[143] Xue R., Qin C., Li L., Huang L., Tang K., Chen J., Liang H., Xu H., Qin X., Yang C. (2025). SRF/SLC31A1 signaling promotes cuproptosis induced by celastrol in NSCLC. Int. Immunopharmacol..

[144] Liu Y., Zhang J., Lai C., Wang W., Dai X., Qian X., Chen D., Zhao P. (2025). Injectable celastrol-loading emulsion hydrogel for immunotherapy of low-immunogenic cancer. J. Nanobiotechnol..

[145] Yoon M.J., Lee A.R., Jeong S.A., Kim Y.-S., Kim J.Y., Kwon Y.-J., Choi K.S. (2014). Release of Ca2+ from the endoplasmic reticulum and its subsequent influx into mitochondria trigger celastrol-induced paraptosis in cancer cells. Oncotarget.

[146] Park Y.J., Cho M.-Y., Kim I.Y., Lee D.M., Yoon G., Choi K.S. (2025). mTORC2/Akt axis promotes proteotoxic stress and mitochondrial Ca2+ overload during celastrol-induced paraptosis. Biochem. Biophys. Res. Commun..

[147] Yang Y., Cheng S., Liang G., Honggang L., Wu H. (2018). Celastrol inhibits cancer metastasis by suppressing M2-like polarization of macrophages. Biochem. Biophys. Res. Commun..

[148] Yu X., Zhao Q., Zhang X., Zhang H., Liu Y., Wu X., Li M., Li X. (2017). Celastrol ameliorates inflammation through inhibition of NLRP3 inflammasome activation. Oncotarget.

[149] Sang X., Chen Y., Chen W., Xie J., Meng G., Zhong J., Li T., Lu A. (2018). Celastrol specifically inhibits the activation of NLRP3 inflammasome. Sci. China Life Sci..

[150] Lee H.E., Lee J.Y., Yang G., Kang H.C., Cho Y.-Y., Lee H.S., Lee J.Y. (2019). Inhibition of NLRP3 inflammasome in tumor microenvironment leads to suppression of metastatic potential of cancer cells. Sci. Rep..

[151] Saber S., Ghanim A.M.H., El-Ahwany E. (2020). Novel complementary antitumor effects of celastrol and metformin by targeting IκBκB, apoptosis and NLRP3 inflammasome activation in diethylnitrosamine-induced murine hepatocarcinogenesis. Cancer Chemother. Pharmacol..

[152] Mezzanotte L., An N., Mol I.M., Löwik C.W.G.M., Kaijzel E.L. (2014). A New Multicolor Bioluminescence Imaging Platform to Investigate NF-κB Activity and Apoptosis in Human Breast Cancer Cells. PLoS ONE.

[153] Ou G., Jiang X., Gao A., Li X., Lin Z., Pei S. (2021). Celastrol Inhibits Canine Mammary Tumor Cells by Inducing Apoptosis via the Caspase Pathway. Front. Vet. Sci..

[154] Jiang Y., Shi Y., Lv M., Wang T., Wang P., Yuan X., Gao F., Ma B. (2025). Celastrol modulates IRS1 expression to alleviate ovarian aging and to enhance follicular development. Cell Biol. Toxicol..

[155] Yuan L., Zhou W., Zhou D., Liu X., Li X. (2023). Celastrol exerts anti-ER+ breast cancer by activating endoplasmic reticulum stress to induce apoptosis. J. Army Med. Univ..

[156] Cao L., Zhang X., Cao F., Wang Y., Shen Y., Yang C., Uzan G., Peng B., Zhang D. (2015). Inhibiting inducible miR-223 further reduces viable cells in human cancer cell lines MCF-7 and PC3 treated by celastrol. BMC Cancer.

[157] Wang X., Liu Q., Wu S., Xu N., Li H., Feng A. (2022). Identifying the Effect of Celastrol Against Ovarian Cancer with Network Pharmacology and In Vitro Experiments. Front. Pharmacol..

[158] Zhu H., Liu X.-W., Ding W.-J., Xu D.-Q., Zhao Y.-C., Lu W., He Q.-J., Yang B. (2010). Up-regulation of death receptor 4 and 5 by celastrol enhances the anti-cancer activity of TRAIL/Apo-2L. Cancer Lett..

[159] Zhang H., Li J., Li G., Wang S. (2016). Effects of celastrol on enhancing apoptosis of ovarian cancer cells via the downregulation of microRNA-21 and the suppression of the PI3K/Akt-NF-κB signaling pathway in an in vitro model of ovarian carcinoma. Mol. Med. Rep..

[160] Salvioni L., Rizzuto M.A., Bertolini J.A., Pandolfi L., Colombo M., Prosperi D. (2019). Thirty Years of Cancer Nanomedicine: Success, Frustration, and Hope. Cancers.

[161] Labhasetwar V. (2005). Nanotechnology for drug and gene therapy: The importance of understanding molecular mechanisms of delivery. Curr. Opin. Biotechnol..

[162] Singh D., Kumar A. (2025). Next-generation nanocarriers for precision antitumor therapy: From passive targeting to intelligent response. Explor. Target. Antitumor Ther..

[163] Spencer D.S., Puranik A.S., Peppas N.A. (2015). Intelligent nanoparticles for advanced drug delivery in cancer treatment. Curr. Opin. Chem. Eng..

[164] Zhao Y., Yao Y., Fan S., Shen X., Chai X., Li Z., Zeng J., Pi J., Zhou Z., Huang G. (2024). Oral delivery of pH-sensitive nanoparticles loaded Celastrol targeting the inflammatory colons to treat ulcerative colitis. J. Tissue Eng..

[165] Szebeni J., Alving C.R., Rosivall L., Bünger R., Baranyi L., Bedöcs P., Tóth M., Barenholz Y. (2007). Animal Models of Complement-Mediated Hypersensitivity Reactions to Liposomes and Other Lipid-Based Nanoparticles. J. Liposome Res..

[166] Niu W., Wang J., Wang Q., Shen J. (2020). Celastrol Loaded Nanoparticles with ROS-Response and ROS-Inducer for the Treatment of Ovarian Cancer. Front. Chem..

[167] Li Y., Zhang H., Wei J., Jin W., Tong Q., Ye Y., Xie Q., Li Q., Cheng G., Xiong Y. (2025). Celastrol liposomes enhance anti-PD-L1 in triple-negative breast cancer by reprogramming immunosuppressive microenvironment driven by cancer-associated adipocytes. Phytomedicine.

[168] Zhang W., Li J., Gao X., Liu L., Liang H., Yue L. (2025). Engineered celastrol liposomes with glycyrrhizic acid augment synergistic antitumor efficacy in breast cancer. Front. Oncol..

[169] Soe Z.C., Thapa R.K., Ou W., Gautam M., Nguyen H.T., Jin S.G., Ku S.K., Oh K.T., Choi H.-G., Yong C.S. (2018). Folate receptor-mediated celastrol and irinotecan combination delivery using liposomes for effective chemotherapy. Colloids Surf. B Biointerfaces.

[170] Li C., Wang Z., Zhang Y., Zhu Y., Xu M., Lei H., Zhang D. (2024). Efficient Sequential Co-Delivery Nanosystem for Inhibition of Tumor and Tumor-Associated Fibroblast-Induced Resistance and Metastasis. Int. J. Nanomed..

[171] Huang J., Shi J., Ma N., Li Y., Jin W., Zhang H., Zhang X., Luo N., Ding Y., Xie Q. (2025). Celastrol-loaded ginsenoside Rg3 liposomes enhance anti-programmed death ligand 1 immunotherapy by inducing immunogenic cell death in triple-negative breast cancer. Phytomedicine.

[172] Cheng Q., Zhong X., Deng L., He X., Luo M., Wang R., Zhang J. (2025). RBC Membrane-Camouflaged Nanosystem-Mediated Synergistic Drug Combination for Enhanced Anti-Tumor Therapy. Adv. Healthc. Mater..

[173] Qin Y., Liu T., Guo M., Liu Y., Liu C., Chen Y., Qu D. (2020). Mild-heat-inducible sequentially released liposomal complex remodels the tumor microenvironment and reinforces anti-breast-cancer therapy. Biomater. Sci..

[174] Chen Y., Guo M., Qu D., Liu Y., Guo J., Chen Y. (2020). Furin-responsive triterpenine-based liposomal complex enhances anticervical cancer therapy through size modulation. Drug Deliv..

[175] Chen Y., Qu D., Fu R., Guo M., Qin Y., Guo J., Chen Y. (2018). A Tf-modified tripterine-loaded coix seed oil microemulsion enhances anti-cervical cancer treatment. Int. J. Nanomed..

[176] Guo M., Qu D., Qin Y., Chen Y., Liu Y., Huang M., Chen Y. (2019). Transferrin-functionalized microemulsions coloaded with coix seed oil and tripterine deeply penetrate to improve cervical cancer therapy. Mol. Pharm..

[177] Wang X., Zhang H., Li C., Cai L., Lin L., Lin Y., Xu W. (2025). Tumor-targeting and redox dual-responsive hyaluronic acid-based polymeric micelles loaded with celastrol for breast cancer treatment. Int. J. Biol. Macromol..

[178] Tan Y., Zhu Y., Zhao Y., Wen L., Meng T., Liu X., Yang X., Dai S., Yuan H., Hu F. (2018). Mitochondrial alkaline pH-responsive drug release mediated by Celastrol loaded glycolipid-like micelles for cancer therapy. Biomaterials.

[179] Qin Y., Wang Z., Wang X., Zhang T., Hu Y., Wang D., Sun H., Zhang L., Zhu Y. (2023). Therapeutic effect of multifunctional celastrol nanoparticles with mitochondrial alkaline drug release in breast cancer. Mater. Today Adv..

[180] Qian Y., Zhang J., Xu R., Li Q., Shen Q., Zhu G. (2021). Nanoparticles based on polymers modified with pH-sensitive molecular switch and low molecular weight heparin carrying Celastrol and ferrocene for breast cancer treatment. Int. J. Biol. Macromol..

[181] Huang T., Wang Y., Shen Y., Ao H., Guo Y., Han M., Wang X. (2020). Preparation of high drug-loading celastrol nanosuspensions and their anti-breast cancer activities in vitro *and* in vivo. Sci. Rep..

[182] Li X., Zhang M., Wang X., Ma P., Song Y. (2025). Carrier-Free Nanomedicine Based on Celastrol and Methotrexate for Synergistic Treatment of Breast Cancer via Folate Targeting. Int. J. Nanomed..

[183] Zeng H., Zeng X., Wang C., Wang G., Tian Q., Zhao J., Zhao L., Li R., Luo Y., Peng H. (2025). Combination therapy using Cel-CSO/Taxol NPs for reversing drug resistance in breast cancer through inhibiting PI3K/AKT/NF-κB/HIF-1α pathway. Drug Deliv. Transl. Res..

[184] Guo Y., Ding L., Lang X., You J., Wu D., Wang X., Zhang Y., Liu W., Dong Z. (2025). Dual-targeting chemoimmunotherapy co-delivery system based on self-assembly and core-shell structure. Biomed. Pharmacother..

[185] Abdelmoneem M.A., Abd Elwakil M.M., Khattab S.N., Helmy M.W., Bekhit A.A., Abdulkader M.A., Zaky A., Teleb M., Elkhodairy K.A., Albericio F. (2021). Lactoferrin-dual drug nanoconjugate: Synergistic anti-tumor efficacy of docetaxel and the NF-κB inhibitor celastrol. Mater. Sci. Eng. C.

[186] Li X., Yu C., Zheng G., Li Y., Cao W., Wang F. (2025). Development and Evaluation of Liposomal Celastrol-PROTACs for Treating Triple-Negative Breast Cancer. Pharmaceuticals.

[187] Law S., Leung A.W., Xu C. (2020). Folic acid-modified celastrol nanoparticles: Synthesis, characterization, anticancer activity in 2D and 3D breast cancer models. Artif. Cells Nanomed. Biotechnol..

[188] Zhang W., Li J., Yue L., Ji C. (2024). Targeted Delivery of Celastrol by GA-Modified Liposomal Calcium Carbonate Nanoparticles to Enhance Antitumor Efficacy Against Breast Cancer. Pharmaceutics.

[189] Zhou R., You Y., Zha Z., Chen J., Li Y., Chen X., Chen X., Jiang X., Chen J., Kwan H.Y. (2023). Biotin decorated celastrol-loaded ZIF-8 nano-drug delivery system targeted epithelial ovarian cancer therapy. Biomed. Pharmacother..

[190] Yang L., Zhang Y., Chen T., Han J., Wang H., Zhang M., Bao Q., Diao Z., Zhang T., Jiang Y. (2025). Dual Targeting Biomimetic Nanoplatform Eradicates Oncogenic Bacteria While Reinvigorating Anti-Tumor Immunity in Breast Cancer. ACS Appl. Mater. Interfaces.

[191] Bo Y., Wang Y., Liu Y., Liu J., Shen H., Wang S. (2024). Green Synthesis of Gold Nanoparticles by Celastrol for Chemotherapy and Photothermal Anticancer Therapy. Mater. Open.

[192] Mao Y., Chu X., Xie F., Fu L., Ding Z., Zhang W., Zhang Q., Tang C., Zhu S., Cao W. (2025). Estimates and projections of the global economic cost of breast cancers from 2021 to 2050. Front. Endocrinol..

[193] Ma Y., Lai X., Fang H. (2025). Global, regional, and national disease burden and economic costs of cervical cancer (1991–2021): A multidimensional data synthesis analysis. Front. Public Health.

[194] Hutchinson B., Euripides M., Reid F., Allman G., Morrell L., Spencer G., Ilbawi A., Meheus F., Gaafar H., Casolino R. (2025). Socioeconomic Burden of Ovarian Cancer in 11 Countries. JCO Glob. Oncol..

[195] Tu Y., Dai G., Chen Y., Tan L., Liu H., Chen M. (2025). Emerging Target Discovery Strategies Drive the Decoding of Therapeutic Power of Natural Products and Further Drug Development: A Case Study of Celastrol. Exploration.

[196] Ye H., Wang Y., Zhang X., Yang L., Cai B., Zhang D., Peng B. (2025). Characterization of global research trends and prospects on celastrol, a principal bioactive ingredient of *Tripterygium wilfordii* Hook F: Bibliometric analysis. Pharm. Biol..

[197] Zhao Y., Hansen N.L., Duan Y.-T., Prasad M., Motawia M.S., Møller B.L., Pateraki I., Staerk D., Bak S., Miettinen K. (2023). Biosynthesis and biotechnological production of the anti-obesity agent celastrol. Nat. Chem..

